# Unraveling the increasing burden of allergies: Spatiotemporal dynamics of pollen allergy risk in China

**DOI:** 10.1016/j.eehl.2026.100267

**Published:** 2026-07-30

**Authors:** Bo Cao, Chengke Bai, Kunyi Wu, Ting La, Nan Xu, Lianjin Liu, Xiaofang Zhou, Chong Chen, Tiantian Bin, Shan Feng, Yali Zhang, Guishuang Li

**Affiliations:** a Department of Otolaryngology, The Second Affiliated Hospital of Xi'an Jiaotong University, Xi'an, 710004, China; b Core Research Laboratory, The Second Affiliated Hospital of Xi'an Jiaotong University, Xi'an, 710004, China; c College of Life Sciences, Shaanxi Normal University, Xi'an, 710119, China; d National-Local Joint Engineering Research Center of Biodiagnosis & Biotherapy, The Second Affiliated Hospital of Xi'an Jiaotong University, Xi'an, 710004, China; e Department of Clinical Laboratory, The Second Affiliated Hospital of Xi'an Jiaotong University, Xi'an, 710004, China

**Keywords:** Pollen allergy, Allergenic plants, Risk distribution, Climate change, Global warming

## Abstract

Pollen allergies have increased significantly worldwide in recent years. However, its mechanism from an ecological perspective has not been fully elucidated. Here, we report, for the first time, the spatial distribution patterns of artificially planted allergenic plants (APAPs) and naturally distributed allergenic plants (NDAPs) in China. Field investigations (2007–2023) and ecological niche modeling efforts revealed that APAPs are concentrated in economically developed urban regions, while NDAPs exhibit distinct ecological niches across climatic gradients. Further risk analysis indicates that pollen allergy risk correlates significantly with socio-economic and environmental factors, demonstrating that regions southeast of the Heihe-Tengchong Line are particularly vulnerable. Future climate scenarios show a substantial increase in NDAP distributions and associated allergy risks by 2100, highlighting a critical expansion of high-suitability areas for allergenic plants. Further monitoring across diverse climatic regions elucidates the ecological mechanisms driving increased pollen allergies, with warmer temperatures leading to prolonged flowering periods and higher pollen counts. These findings highlight the urgent need for adaptive public health strategies to mitigate the impact of pollen allergies in a rapidly changing environment, offering critical insights for the development of effective intervention programs to address global climate challenges.

## Highlights

• APAPs are concentrated in economically developed urban regions.
• NDAPs have distinct ecological niches across climatic gradients.
• Allergic risks increase under future climate change.
• Increased flowering period and pollen counts lead to growing allergies.

## Graphical abstract

**Figure:**
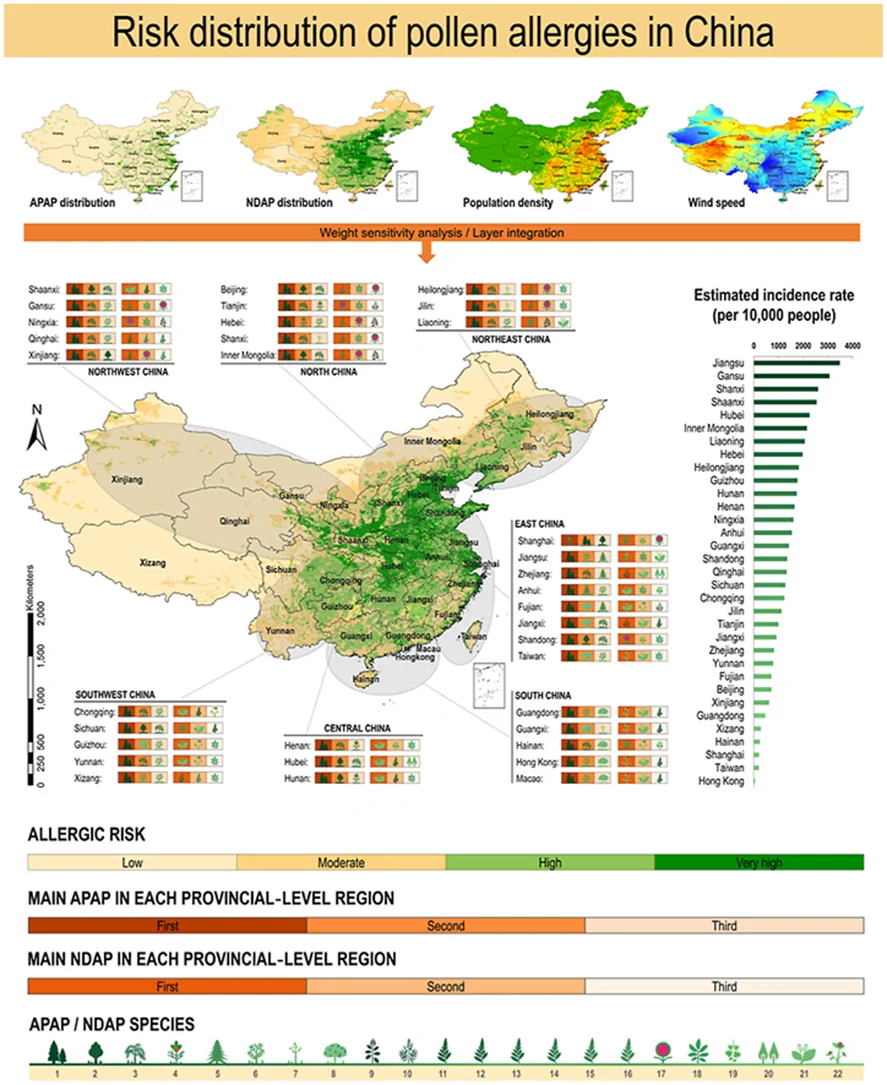

## 1 Introduction

The rising prevalence of pollen allergies represents a significant global public health concern [1]. According to recent estimates, around 10% to 20% of the global population is affected by allergic rhinitis, with pollen allergies being one of the most common triggers [2]. In China, the number of individuals diagnosed with pollen allergies has surged from approximately 25 million in 2000 to over 50 million in 2023, representing a staggering increase of 100% in just over two decades [3,4]. This trend reflects broader patterns of environmental change, urbanization, and lifestyle shifts. Beyond the immediate health impacts, pollen allergies impose a significant socio-economic burden—estimates suggest that they cost the Chinese healthcare system approximately CNY 20 billion (about USD 3 billion) annually in direct medical expenses and lost productivity [5]. In addition, the global increasing prevalence of pollen allergies poses significant challenges to ecological conservation and urban planning [6,7]. The rising incidence of pollen allergies not only affects personal well-being but also places a strain on public health systems, necessitating urgent attention and effective management strategies to mitigate their effects.

Understanding the dynamic changes in pollen allergy risk and the underlying factors contributing to its increase poses a significant challenge in epidemiological studies. The increase in pollen allergies is intricately linked to allergenic plants, which can be divided into two main groups: artificially planted allergenic plants (APAPs) and naturally distributed allergenic plants (NDAPs). Specifically, the classification of APAPs is based on planting purpose and anthropogenic management practices, rather than the native status of the species. For example, species within the genera *Pinus*, *Populus*, and *Salix* are native to China, but they are categorized as APAPs when intentionally cultivated in urban green spaces (e.g., parks, roadside verges, and residential landscapes) for ornamental or ecological landscaping functions [4,7]. APAPs include a variety of ornamental and landscape plants, such as pine (*Pinus* spp.), poplar (*Populus* spp.), and willow (*Salix* spp.), which are commonly found in parks, gardens, and along city streets. On the other hand, NDAPs encompass wild plants that thrive in specific ecosystems, including native grasses, trees, and shrubs. For instance, species like *Artemisia* (wormwood) and different types of flowering weeds are prevalent in rural and semi-urban regions, contributing significantly to pollen counts during flowering seasons [8]. Furthermore, as urbanization continues to expand (e.g., China’s urban population is expected to reach 1 billion by 2030), the relationship between plant distribution and pollen-related allergies becomes even more complex [9,10]. Therefore, understanding the spatial distribution of both APAPs and NDAPs is crucial for predicting and preventing pollen allergies, especially in the context of accelerated urbanization and global climate change.

Despite the known association between pollen allergies and specific plant species, the spatial distribution and future trends of these plants in China remain inadequately understood. The dynamics of pollen-producing plants are influenced by a multitude of factors, including climate change, urbanization, and population density [6,11]. For instance, it is projected that under high-emission scenario, mean temperatures across China will rise by approximately 5 °C for the period 2081–2100 (the end of the 21st century) relative to the baseline period 1986–2005 [12]. Such drastic warming is likely to alter plant growth patterns and prolong flowering periods with elevated pollen production. Concurrent shifts in urban ecological conditions may facilitate the colonization of non-native plant species and induce local extinctions of native taxa [13]. Additionally, demographic factors such as population density (e.g., exceeding 1000 people per km^2^) and economic growth (e.g., China’s GDP increasing over tenfold from 2003 to 2023) would significantly influence pollen exposure levels and allergy prevalence [14]. Revealing the mechanisms behind these interactions will provide valuable insights into how environmental changes contribute to the rise of pollen allergies, thus facilitating the development of more effective prevention and management strategies.

Previous studies have revealed significant patterns in the distribution and drivers of allergenic plants across China. Geographically, *Artemisia* species dominate northern and northwestern regions, while *Morus* species show higher prevalence in eastern urban centers, correlating with regional climate characteristics and urbanization gradients [15]. Climate change plays a critical role in the distribution of allergenic plants. Rising temperatures are expanding the potential habitat of the thermophilic species *Ambrosia* (ragweed) northward in Heilongjiang Province, the northernmost province in China [16]. Meanwhile, socio-economic factors demonstrate complex interactions. With rapid urbanization, the urban population in China sharply increased from 690.79 million in 2011 to 914.25 million in 2021. During the same period, the per capita green area rose from 11.8 m^2^ to 14.8 m^2^, increasing the risk of pollen exposure for urban residents [[17], [18], [19]]. Critically, these drivers exhibit synergistic effects. Climate-driven vegetation shifts coincide with urban expansion patterns, creating novel pollen exposure hotspots where warming climates and intensive landscaping intersect, particularly in emerging megacity clusters like the “Beijing-Tianjin-Hebei” region [20]. However, existing studies predominantly focus on individual factors, leaving critical gaps in understanding their compound impacts on pollen allergy epidemiology.

In this study, we employed a multifaceted approach that included field investigation, model prediction, and clinical validation to comprehensively investigate the spatial distribution and future changes of major pollen-producing plants in China. Utilizing research approaches from multiple dimensions, this study aims to generate a detailed spatiotemporal variation map of the main allergenic plants and to uncover ecological and environmental mechanisms driving pollen allergy prevalence. By integrating environmental factors (e.g., wind speed), socio-economic factors (e.g., population density), and clinical data of allergy incidence, we further mapped the risk distribution of pollen allergies in China and estimated the incidence of allergies in different regions. Collectively, the results of this study will provide valuable references for the prevention and control of pollen allergies in China. Moreover, these results will offer important insights into the global implications of pollen allergies within the broader context of environmental change.

## 2 Materials and methods

### 2.1 Methodological framework

**Fig. 1:**
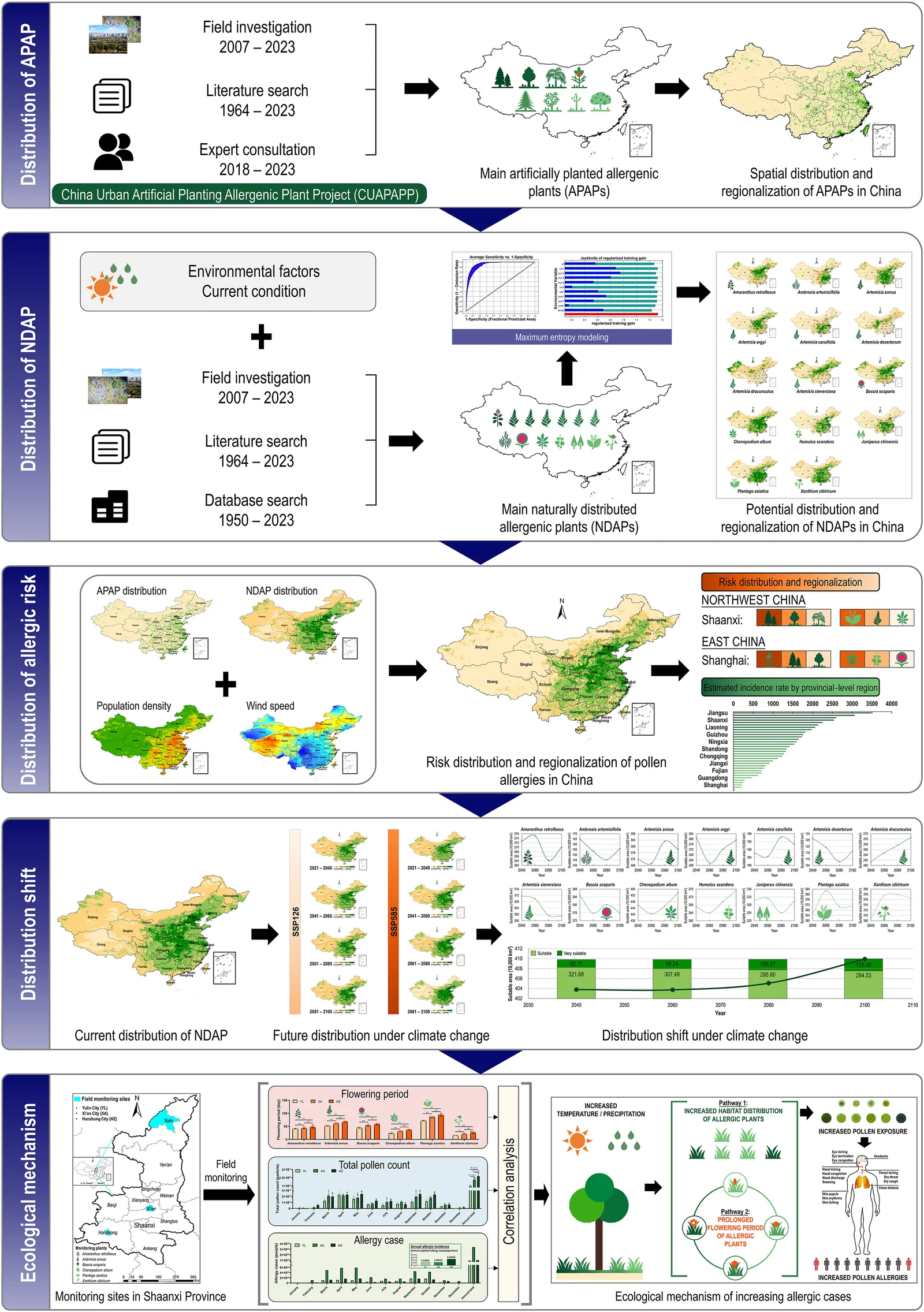
Experimental design for investigating spatiotemporal dynamics of pollen allergy risk in China. The methodological framework of this study includes five parts as follows: (1) Distribution of APAPs; (2) Distribution of NDAPs; (3) Distribution of allergy risk; (4) Distribution shift; and (5) Ecological mechanism. APAPs, artificially planted allergenic plants; NDAPs, naturally distributed allergenic plants.

The methodological framework of this study includes five parts as follows (Fig. 1):

Distribution of APAPs. The distribution of APAPs was investigated within the framework of the China Urban Artificial Planting Allergenic Plant Project (CUAPAPP) initiated by our group. We identified the primary artificial planting species across eight genera (*Pinus*, *Populus*, *Salix*, *Platanus*, *Metasequoia*, *Cupressus*, *Ulmus*, and *Morus*) and developed a comprehensive APAP distribution map based on the green space distribution in China. This analysis draws on extensive field surveys conducted from 2007 to 2023, image analysis from 2015 to 2023, a review of literature spanning from 1964 to 2023, and consultations with experts in the field. Utilizing these resources, we created a detailed spatial distribution and zoning map for APAPs across 34 provincial-level regions in China.

Distribution of NDAPs. The distribution of NDAPs was primarily predicted using the species distribution model (SDM) - maximum entropy (Maxent) modeling. This study focused on 14 main NDAP species (*Amaranthus retroflexus*, *Ambrosia artemisiifolia*, *Artemisia annua*, *Artemisia argyi*, *Artemisia caruifolia*, *Artemisia desertorum*, *Artemisia dracunculus*, *Artemisia sieversiana*, *Bassia scoparia*, *Chenopodium album*, *Humulus scandens*, *Juniperus chinensis*, *Plantago asiatica*, and *Xanthium sibiricum*) in China, for which species occurrence data were collected through field surveys conducted between 2007 and 2023, supplemented by literature reviews and database searches from 1964 to 2023. The environmental factors determining species distribution were obtained from the WorldClim database. Utilizing Maxent modeling, we generated detailed spatial distribution and zoning maps for the main NDAP species in China.

Distribution of allergy risk. The allergy risk distribution was derived by integrating the APAP/NDAP distribution maps with critical determinants of allergy risk, including population density and wind speed. This analysis enabled us to create spatial distribution and zoning maps of allergy risk across the 34 provincial-level regions in China (see Section 2.8). Furthermore, we estimated the allergy incidence rate for each provincial-level region based on available clinical data regarding allergy prevalence.

Distribution shift. The distribution shift of NDAPs was projected by combining NDAP occurrence data with future environmental factors under two representative shared socio-economic pathways (SSPs): SSP126 (low-emission scenario) and SSP585 (high-emission scenario). Our models predicted the distribution of the 14 NDAP species at future time points (2040, 2060, 2080, 2100) and depicted the total distribution pattern by overlaying distribution maps. Further statistical analysis revealed trends and shifts in NDAP distribution over time.

Ecological mechanism. To elucidate the ecological mechanism contributing to the rising incidence of allergy cases in clinical practice, we conducted a field investigation of six key NDAPs (*A. retroflexus*, *A. annua*, *B. scoparia*, *C. album*, *P. asiatica*, and *X. sibiricum*) in Shaanxi Province, China. We selected three distinct regions representing northern, central, and southern Shaanxi [21]. In each of these regions, we monitored flowering periods, total pollen counts, and clinical allergy cases associated with these species. By assessing the correlations between NDAP flowering and allergic incidence under different environmental conditions, we proposed the ecological mechanism behind the continuous rise of allergy cases (see Section 2.11).

### 2.2 Species distribution patterns of APAPs

In this study, we conducted an in-depth investigation of the distribution pattern of APAPs from eight genera through a multifaceted approach involving field surveys, comprehensive literature reviews, and consultations with subject matter experts. The detailed methods were as follows (Fig. S1):

Study area stratification. Based on China’s administrative divisions, we initially selected 293 city-level divisions across 34 provincial-level regions. These city-level divisions represent China's diverse climatic zones, urbanization levels, and economic development stages, making them suitable for analyzing urban-scale allergenic plant distribution and pollen allergy risk. Using the urban green space distribution map as the stratification criterion, we identified 53 key city-level divisions from 25 provincial-level regions within core green space concentration areas (green coverage >30%). This 30% threshold was established based on the provincial-level average green coverage rate of urban built-up areas in China, as officially reported by the National Bureau of Statistics (2021) [18]. The remaining 240 city-level divisions were classified as supplementary survey areas with lower green coverage (≤30%) (Table S1). The eight APAP genera targeted in this study (*Pinus*, *Populus*, *Salix*, *Platanus*, *Metasequoia*, *Cupressus*, *Ulmus*, *Morus*) were selected based on two core criteria: first, these genera represent the most widely used and dominant landscaping plants in urban green spaces across China; second, numerous clinical and aerobiological studies have confirmed that these genera produce high quantities of allergenic pollen grains, which are major triggers of pollen-induced allergic rhinitis in Chinese urban populations [15].

Data collection. For field surveys (2007–2023), we conducted systematic surveys using 1 km × 1 km grid sampling units in the 53 key city-level divisions. Each city-level division contained 10–15 randomly distributed sampling grids, with survey timing standardized to peak pollen seasons (March to October). For each grid, we recorded: 1) APAP species presence/absence; 2) species density estimates using quadrant counting (10 m × 10 m subplots; five replicate quadrants were randomly placed); and 3) GPS coordinates of APAP locations. For image analysis (2015–2023), we implemented a dual-verification system combining Baidu Street View analysis (https://map.baidu.com) and satellite imagery interpretation in the 240 supplementary city-level divisions. Using the online image database, we systematically captured 100 street view images at 500 m intervals along urban roads in each city-level division. APAP species were identified by trained researchers using leaf/trunk characteristics (response variable: APAP species presence/absence). For literature reviews (1964–2023), we compiled 796 peer-reviewed papers through systematic searches in CNKI, Web of Science, and PubMed using keywords: (“allergenic plants” OR “pollen allergy”) AND (“China” OR “Chinese”) (Table S2). Records were included if they met the following criteria: 1) field-collected or clinically diagnosed records; 2) clear geographic coordinates. Duplicate records were excluded. Finally, the collected data were further verified by botany taxonomists and clinical allergists.

Distribution mapping. The final APAP distribution pattern map was generated with the main APAP species at the provincial level based on OSM and UGS-1m urban green space distribution maps (Open Street Map, https://www.openstreetmap.org) [22]. For further layer integration with NDAP distribution, a 1 km × 1 km resolution raster layer was created by converting all validated presence points to kernel density surfaces (search radius = 50 km, output cell size = 1 km^2^). Four distribution levels were re-classified based on percentiles: low (0–25%), medium (26%–50%), high (51%–75%), and very high (76%–100%). Ultimately, we produced a comprehensive distribution and zoning map of APAPs across China.

### 2.3 Species occurrence records of NDAPs

The 14 NDAP species were rigorously selected following two essential criteria to ensure their ecological and clinical relevance: (1) They exhibit broad geographic distributions across multiple climatic zones in China, thus representing the dominant wild allergenic plant taxa in diverse ecosystems; (2) Clinical data from the Pollen Allergy Monitoring Alliance Hospitals (2007–2023) identified these species as the primary contributors to outpatient pollen allergy cases nationwide [4,23]. In this study, we collected species occurrence records for 14 main allergenic plant species using a comprehensive approach that included field investigation, literature review, and database search (Table S3). We obtained occurrence records with clearly defined taxonomic identities from sources including the National Plant Specimen Resource Center (NPSRC; https://www.cvh.ac.cn) and the Global Biodiversity Information Facility (GBIF; http://www.gbif.org; Table S4). To address the issue of spatial autocorrelation, which can lead to an overestimation of environmental biases and inflate model performance artificially, we utilized the “Spatially Rarefy Occurrence Data Tool” in the SDM Toolbox. This tool effectively reduces spatial clustering and environmental biases in NDAP occurrence records by consolidating occurrences into a single point within a specified Euclidean distance [24,25]. In total, we compiled 16,131 occurrence records of the 14 main NDAP species for subsequent Maxent modeling (Fig. S2).

### 2.4 Environmental factors

To predict the suitable habitat distribution of NDAPs, we adopted a total of 104 environmental variables under current climate conditions, with a spatial resolution of 2.5 arc minutes (approximately 5 km). This dataset covered three categories of variables: topographic factor (Elevation), 19 bioclimatic variables (Bio1–Bio19), as well as monthly climatic indicators including maximum temperature (Tmax1–Tmax12), minimum temperature (Tmin1–Tmin12), mean temperature (Tavg1–Tavg12), precipitation (Prec1–Prec12), solar radiation (Srad1–Srad12), wind speed (Wind1–Wind12), and water vapor pressure (Vapr1–Vapr12). All the above data were retrieved from the WorldClim database (http://www.worldclim.org; Version 2.1) for preliminary analysis. For multicollinearity assessment among these environmental variables, the Pearson correlation coefficient (*r*) was used. For variable pairs with strong correlation (|*r*| ≥ 0.8), only one variable was preserved for subsequent modeling, in accordance with its influence on NDAP growth and development processes (Eqs. 1 and 2).

(1) $${Cov}_{ij}=\frac{\sum_{k=1}^{N}\left(Z_{ik}-\mu_{i}\right)\left(Z_{jk}-\mu_{j}\right)}{N-1}$$

(2) $${Corr}_{ij}=\frac{{Cov}_{ij}}{\delta_{i}\delta_{j}}$$

Where, *Z* denotes the grid value of environmental layers; *i* and *j* represent different environmental layers; *μ* stands for the mean value of environmental layers; *N* is the total number of grids; *k* refers to the grid serial number of environmental layers; *Cov* denotes covariance between two environmental variables; *Corr* represents Pearson correlation coefficient of paired environmental factors.

To project changes in suitable habitats for main NDAPs in the future, future climate scenario data (2.5 arc minutes, about 5 km) were acquired from the WorldClim database. The data involved two SSP scenarios, and four time periods in China (2021–2040, 2041–2060, 2061–2080, 2081–2100) (see Section 2.1). These future environmental variables were simulated by global climate models (GCMs) incorporated in the Coupled Model Intercomparison Project Phase 6 (CMIP6) Assessment Report. In this study, the BCC-CSM2-MR model was selected, considering its excellent performance in simulating future greenhouse gas emission scenarios for China.

### 2.5 Socio-economic factors

Socio-economic factors significantly influence the incidence of pollen allergies. In this study, population density was included as a key social-economic variable in the pollen allergy risk assessment system. Specifically, population density was adopted as a proxy variable rather than a direct causal factor of pollen allergy prevalence. It integrates and represents a suite of underlying social-economic determinants that drive spatial variations in allergy incidence, including regional healthcare accessibility, urban green space configuration, per capita income level, residential greening intensity, and public health awareness. Human population density data for China (30 arc seconds, approximately 1 km resolution; 2020) were obtained from the WorldPop database (https://www.worldpop.org) and processed for analytical purposes. To effectively project the risk of pollen allergies with the NDAP map, the population density layer was reclassified into ten distinct categories and then utilized to evaluate the risks associated with pollen allergies.

### 2.6 Distribution modeling of NDAPs

The suitability distribution of 14 NDAPs was modeled using Maxent version 3.3.3k. For each NDAP species, 100 replicated models were generated to bolster the robustness of the Maxent modeling process. The area under the curve (AUC) was employed as a metric to evaluate model performance. Furthermore, Jackknife analysis was conducted to quantify the contributions of various environmental factors to the suitability distributions of NDAPs. A null-model approach was also implemented to calibrate the modeling outcomes. In this approach, 100 sets of randomly selected occurrence records were generated from the complete dataset of known localities. For each set, a null Maxent model was executed using identical parameter settings as those applied to the actual occurrence records.

### 2.7 Evaluation of modeling results

To evaluate the reliability of the modeling outcomes, we conducted threshold-independent receiver operating characteristic (ROC) analysis. High AUC values obtained from the ROC analysis signify robust results that are statistically distinguishable from random predictions (*P* < 0.05). In this study, the AUC values for all Maxent models corresponding to the 14 NDAP species were subjected to statistical comparison against the expected AUC values derived from a null model at a 95% confidence interval (CI) [26]. Based on these analyses, the original outputs from the Maxent modeling were subsequently processed in ArcGIS (version 10.1) for visual interpretation.

### 2.8 Calculation and classification of NDAP suitability

The habitat suitability (*P*_s_) for each NDAP species was output as a continuous probability surface from the Maxent model, with theoretical values ranging from 0 to 1. Considering that these continuous probability values exhibit a skewed distribution, with the majority of grid cells concentrated in the low-probability range across the study extent, we further divided the calculated *P*_s_ into four distinct classes following previous protocols: (1) low suitability (*P*_s_ < 0.1); (2) moderate suitability (0.1 ≤ *P*_s_ < 0.3); (3) high suitability (0.3 ≤ *P*_s_ < 0.5); and (4) very high suitability (*P*_s_ ≥ 0.5) [24,25,27,28].

### 2.9 Calculation and classification of pollen allergy risk

To eliminate conceptual ambiguity, two distinct pollen allergy-related risks were clearly defined before risk index calculation: (1) Risk of developing allergies: the probability of susceptible individuals becoming sensitized to pollen allergens under real-world environmental exposure; (2) Risk of severe symptoms: the probability of sensitized individuals presenting moderate-to-severe allergic responses (e.g., severe allergic rhinitis, asthma attacks). The index developed in this study represents the risk of developing allergies, driven by pollen exposure intensity and baseline population susceptibility, and does not evaluate the risk of severe symptom exacerbation among already sensitized populations. Following this, the pollen allergy risk (*P*_*r*_) was calculated by overlaying the APAP distribution map, the NDAP suitability map, the human population density map, and the wind speed map (Eq. 3). It should be clarified that wind speed was selected as the key environmental variable for allergy risk spatiotemporal mapping primarily because it directly modulates the long-distance dispersal of airborne pollen and further regulates human exposure to allergenic pollen grains [29,30]. Specifically, this does not imply that wind speed is the only environmental variable involved in the entire study; multiple critical environmental variables (e.g., annual mean temperature, precipitation seasonality, solar radiation) have been comprehensively incorporated into the Maxent modeling of NDAP habitat suitability (see Section 2.4 for detailed methods). To objectively determine the relative contribution of each input layer to *P*_r_, a gradient-based weight-sensitivity analysis was conducted in which the sum of all weights was constrained to 1. Considering the central role of APAPs and NDAPs in driving pollen exposure, the APAP distribution layer and NDAP suitability layer were each assigned a fixed weight of 0.3. Population density and wind speed layers were then adjusted in increments of 0.05 while maintaining the unity constraint. For each combination, the area classified as high-risk and very high-risk was calculated (Table S5). The resulting weight-risk area response curve (Fig. S3) revealed an asymptotic plateau beyond a population density weight of 0.30, indicating diminishing marginal returns. Consequently, the optimal weighting scheme was established as APAP = 0.30, NDAP = 0.30, population density = 0.30, and wind speed = 0.10, balancing ecological relevance with model parsimony. To conduct further statistical analysis, the calculated *P*_r_ was classified into four categories: (1) low risk (*P*_r_ < 0.1); (2) moderate risk (0.1 ≤ *P*_r_ < 0.3); (3) high risk (0.3 ≤ *P*_r_ < 0.5); and (4) very high risk (*P*_r_ ≥ 0.5).

(3) $$P_{r}=S_{A}W_{A}+S_{N}W_{N}+DW_{D}+VW_{wind}$$

Where, *P*_*r*_ is pollen allergy risk; *S*_*A*_ is the suitability index of APAP; *S*_*A*_ is the suitability index of NDAP; *D* represents human population density; *V* stands for wind speed; *W*_*A*_, *W*_*N*_, *W*_*D*_ and *W*_*wind*_ are the weight coefficients for the four input layers, respectively. Based on gradient-based weight-sensitivity analysis, the optimal weighting values are determined as *W*_*A*_ = 0.30, *W*_*N*_ = 0.30, *W*_*D*_ = 0.30, and *W*_*wind*_ = 0.10.

### 2.10 Calculation of estimated incidence rate

The provincial-level incidence rate (*I*_*r*_) of pollen allergy was calculated based on the spatial distribution of risk classes and published national-level prevalence data [31]. Specifically, for each provincial-level region, the number of grid cells classified as high-risk and very high-risk was first counted. A normalized weighted sum of these risk grid cells was then computed as: (number of very high-risk grid cells) + 0.5 × (number of high-risk grid cells). This aggregate value for each provincial-level region was divided by the national total of the same weighted sum to determine the provincial-level region’s proportional share of national high- and very high-risk areas. This proportion was multiplied by the reported total number of individuals at risk of pollen allergy in China to estimate the absolute number of at-risk individuals in the provincial-level region. Finally, the provincial-level incidence rate per 10,000 people was obtained by dividing this estimated number of at-risk individuals by the total population of the provincial-level region and multiplying the result by 10,000. The formula is provided as Eq. 4.

(4) $$I_{r}=\frac{{RasCount}_{prov}}{{RasCount}_{total}}\times \frac{{Pop}_{tr}}{{Pop}_{prov}}\times 10000$$

Where, *I*_*r*_ is the provincial-level incidence rate per 10,000 population; *RasCount*_*prov*_ is the raster count of high and very high classes in each provincial-level region, calculated as [very high + 0.5 × high]; *RasCount*_*total*_ is the raster count of high and very high classes in China, calculated as [very high + 0.5 × high]; *Pop*_*tr*_ is the 2022 nationwide reported population suffering from pollen allergy, the latest dataset available at the time of modeling, with an estimated size of approximately 200 million individuals; *Pop*_*prov*_ is the total population in each provincial-level region in 2023.

### 2.11 Field and clinical validation of the ecological mechanism

To elucidate the ecological mechanism driving the rising incidence of pollen allergies, we conducted a field investigation focusing on six key NDAP species (*A. retroflexus*, *A. annua*, *B. scoparia*, *C. album*, *P. asiatica*, and *X. sibiricum*) across the differential climatic gradient of Shaanxi Province, China, from January to December 2023. Three distinct regions were selected: Yulin (northern; mid-temperate zone), Xi'an (central; warm-temperate zone), and Hanzhong (southern; north subtropical zone). Within each region, three representative sampling points were established in typical habitats of the target species (e.g., open fields, roadsides). At each point, ten individual plants per species were tagged for monitoring. The flowering period was meticulously recorded for each species throughout the pollen season (March to October), defined as the duration (days) from the first observed flowering to the cessation of flowering within the monitored population. Total pollen count was monitored using the gravitational sedimentation method. Due to technical and manpower constraints, we did not distinguish and quantify the pollen amounts of individual allergenic plant species during statistical analysis; instead, we counted the total airborne allergenic pollen quantity in each study area, which included pollen from the six key NDAP species and other allergenic pollen plants in the region. Standard Durham pollen traps were placed at a height of 1.5 m above ground level at each sampling point. Pollen grains were collected every third day at each monitoring site. The collected pollen slides were stained using a glycerol-gelatin mounting medium containing phenol and basic fuchsin (a staining solution that can effectively enhance the contrast of pollen grain walls and internal morphological structures) and identified and quantified under a light microscope at 400× magnification by trained technicians. Clinical allergy case data and geographical areas were obtained from the electronic medical record systems of the Pollen Allergy Monitoring Alliance Hospitals in Shaanxi Province. Cases were included based on a primary diagnosis of allergic rhinitis (ICD-10 code J30.1) coupled with either a positive pollen allergen-specific IgE test result or a physician’s diagnosis explicitly linked to the pollen season. The correlations between annual mean temperature, annual precipitation, flowering period, total pollen count, and population-adjusted allergy incidence rates across the three regions were analyzed using Pearson’s correlation coefficient. Differences in these parameters among the three climatic regions were assessed using one-way analysis of variance (ANOVA). This integrated analysis of ecological and clinical data allowed us to propose a data-driven ecological mechanism for the observed increase in allergy cases.

### 2.12 Base map source

The base map for spatial mapping in this study was sourced from the National Platform for Common Geospatial Information Services of China (http://bzdt.ch.mnr.gov.cn/index.html), with the official map approval number Shen Tu Hao: GS(2020)4619. Strict compliance with national mapping regulations was ensured throughout the process: the base map was used without any alteration, distortion, or modification of administrative boundaries, coastlines, or territorial divisions.

### 2.13 Statistical analysis

The data of NDAP occurrence records, areas of suitable and risk regions, and environmental factors in this study were processed using Microsoft Excel 2016 and the R package (version 4.0.1). The validation of AUCs as well as correlations between suitability shifts and environmental changes were analyzed and plotted using GraphPad Prism (version 9.0). The T-test was employed to compare two groups, while ANOVA was utilized to assess differences among multiple groups. Pearson’s correlation coefficient (*r*) was computed to evaluate the linear relationships among annual mean temperature, annual precipitation, flowering period, total pollen count, and population-adjusted allergy incidence rate. *P* < 0.05 was considered statistically significant.

## 3 Results

### 3.1 Spatial distribution pattern of habitat suitability of pollen allergenic plants in China

The potential habitat distributions of major APAPs and NDAPs across China’s diverse biogeographical regions were systematically investigated and predicted. A total of eight preselected APAP genera were found to be the dominant taxa in urban green spaces, namely *Pinus*, *Populus*, *Salix*, *Platanus*, *Metasequoia*, *Cupressus*, *Ulmus*, and *Morus*. These APAPs were concentrated in urban ecosystems, with their distribution and abundance showing a clear positive correlation with regional economic development levels (Fig. S4, Table S6). The average number of APAP genera was significantly higher in economically developed provinces and municipalities (Beijing, Shanghai, Guangdong), whereas economically underdeveloped provincial-level regions (Qinghai, Xizang) showed a markedly lower average number of APAP genera. Furthermore, distinct regional differences in APAP genera composition were observed: *Pinus*, *Populus*, and *Salix* were the predominant taxa in Beijing, a representative municipality in the North China temperate zone; *Pinus*, *Cupressus*, and *Morus* dominated the APAP community in Guangdong, a typical province in the South China subtropical zone. Overall, APAPs were predominantly distributed in East and South China, accounting for approximately 68% of the total APAP distribution area in China, and these regions were characterized by high economic development and favorable hydrothermal conditions.

The potential distribution of 14 key NDAP species was predicted using the Maxent species distribution model. Model validation was performed via threshold-independent ROC analysis, with AUC values ranging from 0.836 to 0.950 (Figs. S5–S6). All AUC values were significantly higher than the expected values derived from the null model at the 95% CI (*P* < 0.05), indicating robust predictive performance of the model. NDAPs were primarily distributed in natural habitats (e.g., grasslands, wastelands, and rural roadside verges) across China, with distinct spatial patterns corresponding to climatic gradients (Fig. S7, Table S7). Specific distribution characteristics of NDAP species were as follows: *A. desertorum*, *A. dracunculus*, and *A. sieversiana* were concentrated in northern climatic zones, which featured low annual precipitation (100–400 mm) and large annual temperature fluctuations (≥30 °C); *A. artemisiifolia*, *A. argyi*, and *X. sibiricum* were mainly distributed in southern regions with high relative humidity (≥60%) and high annual mean temperatures (14–24 °C). Interspecific variations in the extent of suitable habitats for NDAPs were significant. The area of suitable habitats ranged from 926,198 km^2^ (for *A. desertorum*) to 4,148,646 km^2^ (for *C. album*). The suitable habitat area of *C. album* was approximately 4.5 times that of *A. desertorum*, reflecting the broad ecological amplitude of the former species. Intrageneric distribution differences were also evident in the genus *Artemisia*: *A. desertorum*, *A. dracunculus*, and *A. sieversiana* were confined to northern arid and semi-arid regions, mostly distributed west of the Heihe-Tengchong Line; *A. annua* and *A. argyi* had a broader distribution range, covering the eastern and southern humid zones of China, with their suitable habitats spanning 3,363,385 km^2^ and 2,222,066 km^2^, respectively (Table S7).

### 3.2 Spatial distribution pattern of allergy risk associated with pollen allergenic plants in China

**Fig. 2:**
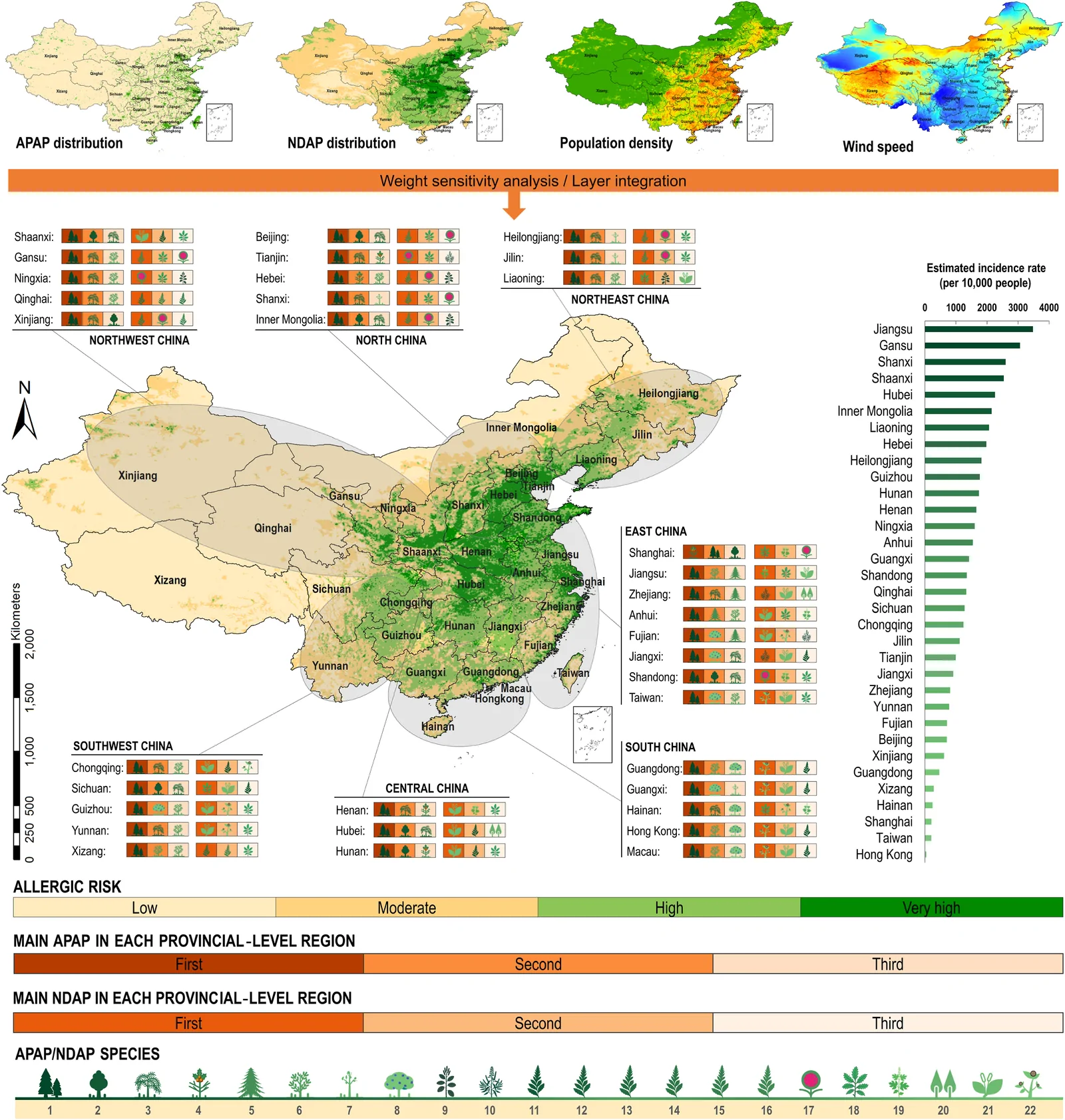
Potential distribution of allergy risks under current conditions in China. The risk distribution was calculated based on APAP/NDAP distribution, population density, and wind speed maps using weight sensitivity analysis and layer integration. The allergy risks were divided into four classes (Class 1: Low risk; Class 2: Moderate risk; Class 3: High risk; Class 4: Very high risk). The colors in the regional legend represent the dominant APAP/NDAP species in each provincial-level region, with darker shades indicating a higher relative importance and contribution of that plant type to overall pollen allergy risk. The estimated incidence rate of allergies in each provincial-level region in China was calculated based on allergy risk and clinical allergic prevalence. APAPs and NDAPs numbered in the figure are: 1. *Pinus*; 2. *Populus*; 3. *Salix*; 4. *Platanus*; 5. *Metasequoia*; 6. *Cupressus*; 7. *Ulmus*; 8. *Morus*; 9. *Amaranthus retroflexus*; 10. *Ambrosia artemisiifolia*; 11. *Artemisia annua*; 12. *Artemisia argyi*; 13. *Artemisia caruifolia*; 14. *Artemisia desertorum*; 15. *Artemisia dracunculus*; 16. *Artemisia sieversiana*; 17. *Bassia scoparia*; 18. *Chenopodium album*; 19. *Humulus scandens*; 20. *Juniperus chinensis*; 21. *Plantago asiatica*; 22. *Xanthium sibiricum*.

We further conducted a comprehensive assessment on the spatial distribution of pollen-induced allergy risk by integrating APAP/NDAP distribution, key socio-economic determinants (human population density), and important environmental variables (wind speed). Our findings elucidate that pollen allergy risk is concentrated in regions southeast of the Heihe-Tengchong Line in China (Fig. 2, Table 1). This encompasses an array of provincial-level regions, including Sichuan (high-risk region: 162,264 km^2^; all subsequent values represent high-risk area), Hunan (159,191 km^2^), Henan (154,574 km^2^), Hebei (150,043 km^2^), Shandong (149,852 km^2^), Hubei (141,085 km^2^), Jiangxi (121,659 km^2^), Anhui (120,826 km^2^), Liaoning (120,427 km^2^) and Guangxi (118,817 km^2^). We further divided the risk distribution into seven distinct regions, observing that the main NDAPs exhibit significant variations across each region. This heterogeneity indicates a significant climate-related distribution pattern that critically influences the risk and prevalence of pollen allergies. Meanwhile, we found that the spatial distribution of pollen allergy risk showed both similarities and differences compared to the suitability distribution of allergenic plants. These differences primarily arise from socio-economic factors, including population density, levels of economic development, urban planning, and other related variables. For example, in the eastern region of Gansu Province, the suitability of NDAP is high; however, the associated allergy risk is low due to the sparse human population density and limited exposure to allergenic pollen during peak dispersal periods. To quantify the epidemiological impact of pollen allergies on the population, we estimated incidence rates based on the distribution of pollen allergy risk, taking into account the prevalence of affected individuals and the spatial distribution of population density. Specifically, Jiangsu had the highest estimated incidence rate of 3479.85 per 10,000 people, followed by Gansu (3059.78), Shanxi (2597.84), Shaanxi (2538.24), Hubei (2254.56), Inner Mongolia Autonomous Region (2146.92), Liaoning (2063.25), Hebei (1974.25), Heilongjiang (1817.88) and Guizhou (1763.49). These provincial-level regions have been identified as the primary high-risk areas, showing significantly elevated estimated incidence rates of allergy cases, which necessitate targeted public health interventions.

**Table 1:** Area of pollen allergy risk and estimated incidence rate by provincial-level regions in China.

Provincial-level region	High (10,000 km^2^)	Very high (10,000 km^2^)	Total [high + very high] (10,000 km^2^)	Estimated incidence rate (per 10,000 people)
Anhui	8.2391	3.8435	12.0826	1540.10
Beijing	0.5051	1.0486	1.5537	704.96
Chongqing	5.2340	0.7170	5.9510	1236.88
Fujian	3.6421	0.6892	4.3313	710.43
Gansu	6.7148	3.0137	9.7285	3059.78
Guangdong	8.2217	0.8472	9.0689	461.95
Guangxi	11.6086	0.2101	11.8187	1416.36
Guizhou	10.9733	0.2708	11.2441	1763.49
Hainan	0.4149	0.0087	0.4236	245.32
Hebei	5.3504	9.6539	15.0043	1974.25
Heilongjiang	7.2443	1.0798	8.3241	1817.88
Henan	3.5779	11.8795	15.4574	1648.61
Hong Kong	0.0538	0	0.0538	42.47
Hubei	5.9805	8.1280	14.1085	2254.56
Hunan	12.5860	3.3331	15.9191	1735.04
Inner Mongolia	7.6488	0.5208	8.1696	2146.92
Jiangsu	4.3105	4.7202	9.0307	3479.85
Jiangxi	11.1816	0.9843	12.1659	912.95
Jilin	7.1402	0.7066	7.8468	1121.34
Liaoning	9.5081	2.5346	12.0427	2063.25
Ningxia	1.5936	0.1892	1.7828	1601.26
Qinghai	0.7239	0.3038	1.0277	1326.85
Shaanxi	5.3434	5.8017	11.1451	2538.24
Shandong	6.9874	7.9978	14.9852	1344.00
Shanghai	0.0451	0.4184	0.4635	209.89
Shanxi	6.4544	4.3765	10.8309	2597.84
Sichuan	14.4522	1.7742	16.2264	1273.29
Taiwan	0.6302	0.0885	0.7187	204.02
Tianjin	0.1302	1.0815	1.2117	995.18
Xizang	0.1580	0.0087	0.1667	284.34
Xinjiang	2.3853	0.1614	2.5467	617.02
Yunnan	5.9232	0.1146	6.0378	779.31
Zhejiang	3.8053	2.6457	6.4510	812.50

### 3.3 Distribution shifts of NDAP suitability and allergy risk under future climate change

**Fig. 3:**
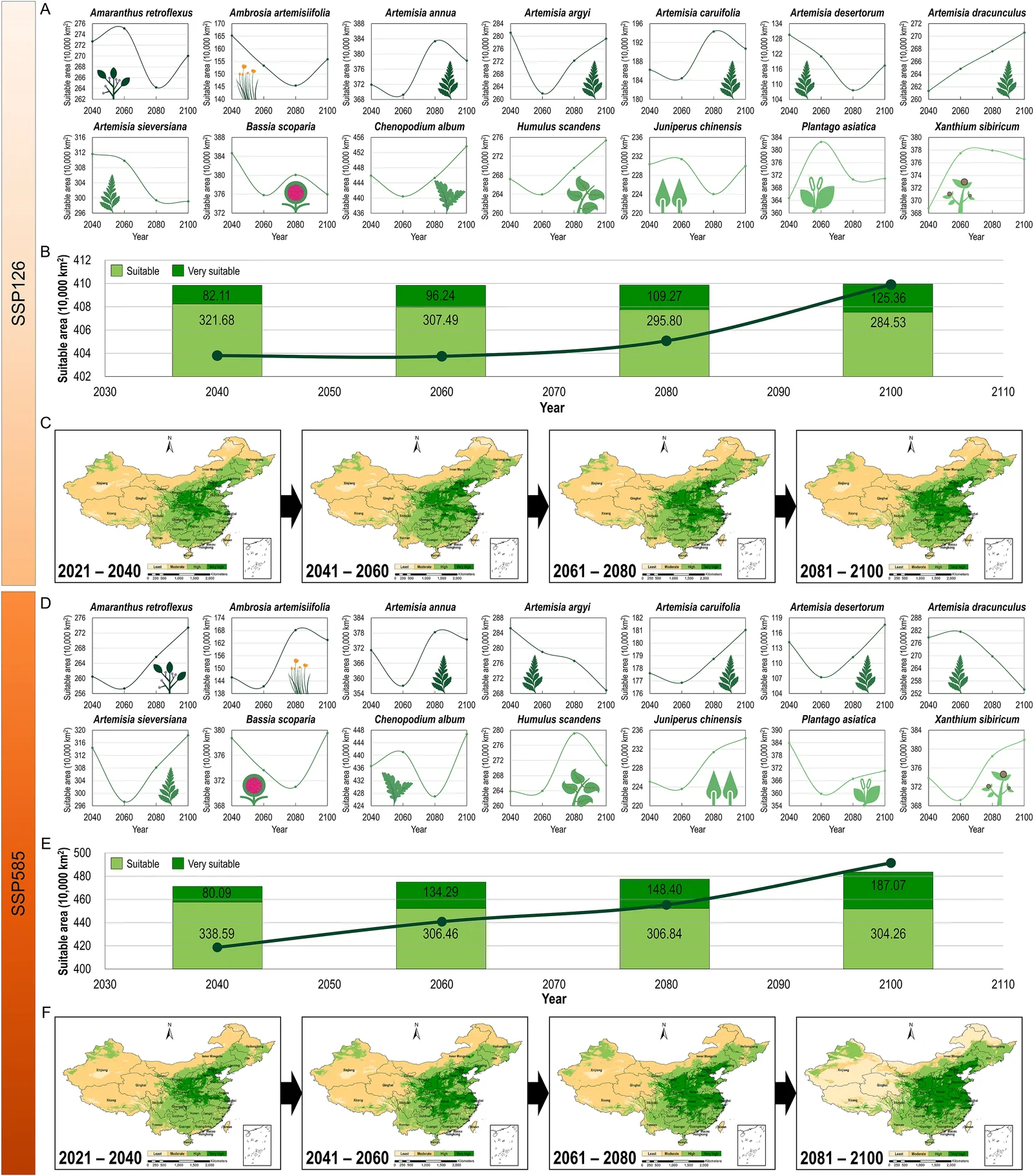
Distribution shifts of main NDAPs under future scenarios. (A−C) Distribution shifts of main NDAPs under low-emission scenario SSP126 from 2040 to 2100. (A) Area of habitat suitability (high + very high) for each NDAP; (B) Area of total habitat suitability (high + very high) for main NDAPs; (C) Spatial distribution shifts of main NDAPs. (D−F) Distribution shifts of main NDAPs under high-emission scenario SSP585 from 2040 to 2100. (D) Area of habitat suitability (high + very high) for each NDAP; (E) Area of total habitat suitability (suitable + very suitable) for main NDAPs; (F) Spatial distribution shifts of main NDAPs. SSPs, shared socio-economic pathways.

We utilized a range of climate change trajectories in predictive scenarios to evaluate potential shifts in allergenic plant distributions and the associated risks of allergens. Two representative SSPs (the lowest: SSP126; the highest: SSP585) under future climate scenarios were used. Given that the future shift of APAP distribution is predominantly influenced by anthropogenic factors (e.g., urban planning), which introduce considerable uncertainty, we selected NDAP species for this predictive analysis. Our findings indicate that, under both scenarios, the cumulative allergy risk associated with NDAP is anticipated to increase significantly by the year 2100 (Fig. 3, Table S8). Under the low-emission scenario, seven species (out of 14) exhibited a discernible upward trend, culminating in a moderate increase of highly suitable regions from 4,037,934 km^2^ (2040) to 4,098,872 km^2^ (2100). Conversely, under a high-emission scenario, eleven species (out of 14) demonstrated a continuous growth trend, with a cumulative increase of highly suitable regions from 4,186,788 km^2^ (2040) to 4,913,333 km^2^ (2100). Significantly, both scenarios suggest a pronounced escalation of distribution shifts in the very high class (SSP126: from 821,181 km^2^ to 1,253,577 km^2^; SSP585: from 800,937 km^2^ to 1,870,694 km^2^; by the year 2100). Taken together, these results indicate a considerable expansion of core distribution areas for allergenic plants (specifically NDAPs), which has critical implications for the prevention and administration of pollen allergic diseases.

### 3.4 Ecological mechanism for growing pollen allergy cases

**Fig. 4:**
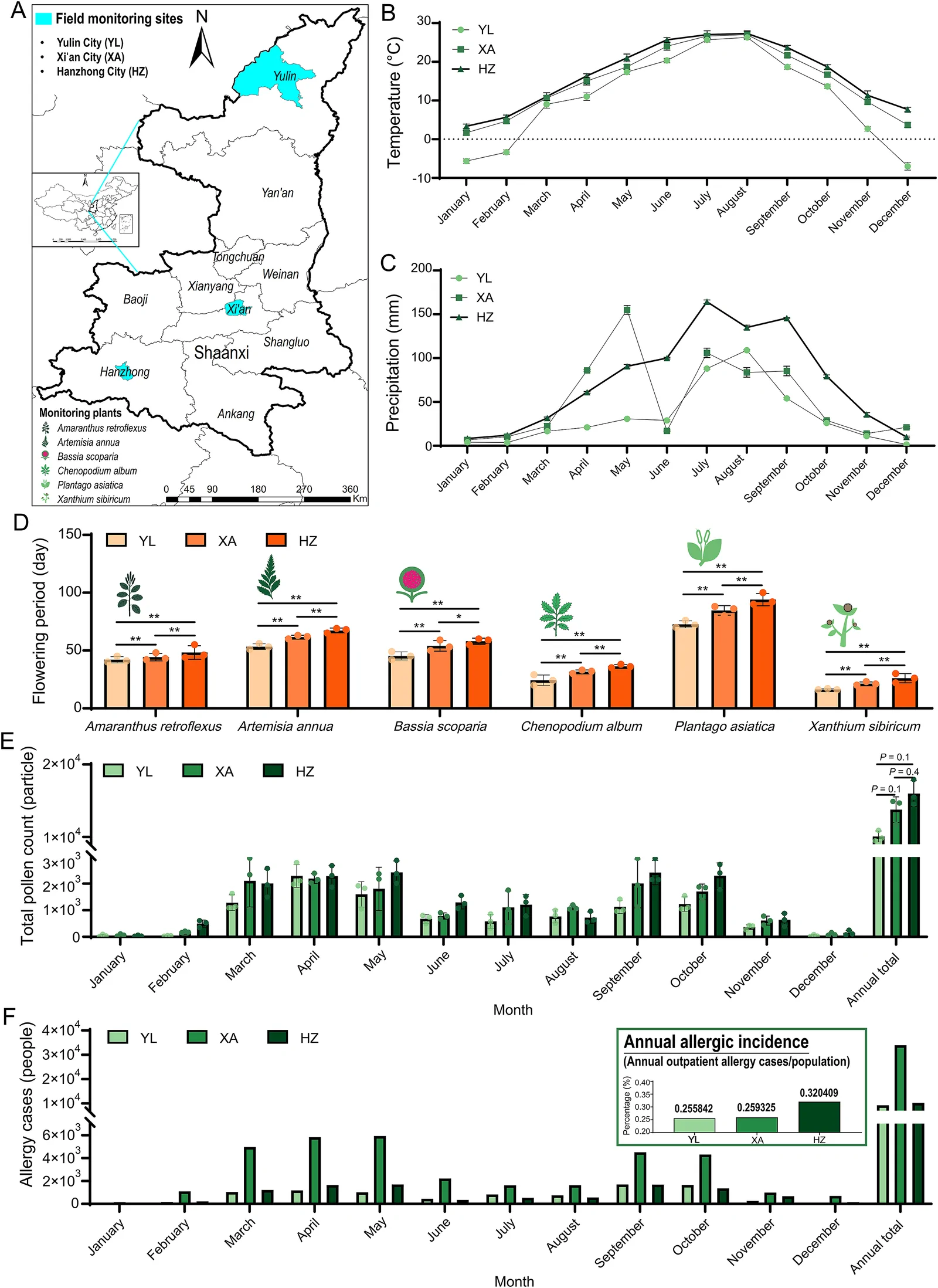
Investigation of the flowering period and total pollen count of key NDAPs and clinical allergy cases in Shaanxi Province, China. (A) Field monitoring sites. For each site, six representative NDAPs were monitored. (B) Monthly and annual mean temperatures. The annual mean temperatures for Yulin (YL), Xi'an (XA) and Hanzhong (HZ) are 10.72 °C, 15.00 °C and 16.56 °C, respectively. (C) Monthly and annual precipitation of monitoring sites. The annual mean precipitation for Yulin (YL), Xi'an (XA) and Hanzhong (HZ) is 394 mm, 636 mm and 874 mm, respectively. (D) Flowering period of monitored NDAPs. (E) Total pollen count of monitoring sites. (F) Outpatient allergy cases in monitoring sites.

**Fig. 5:**
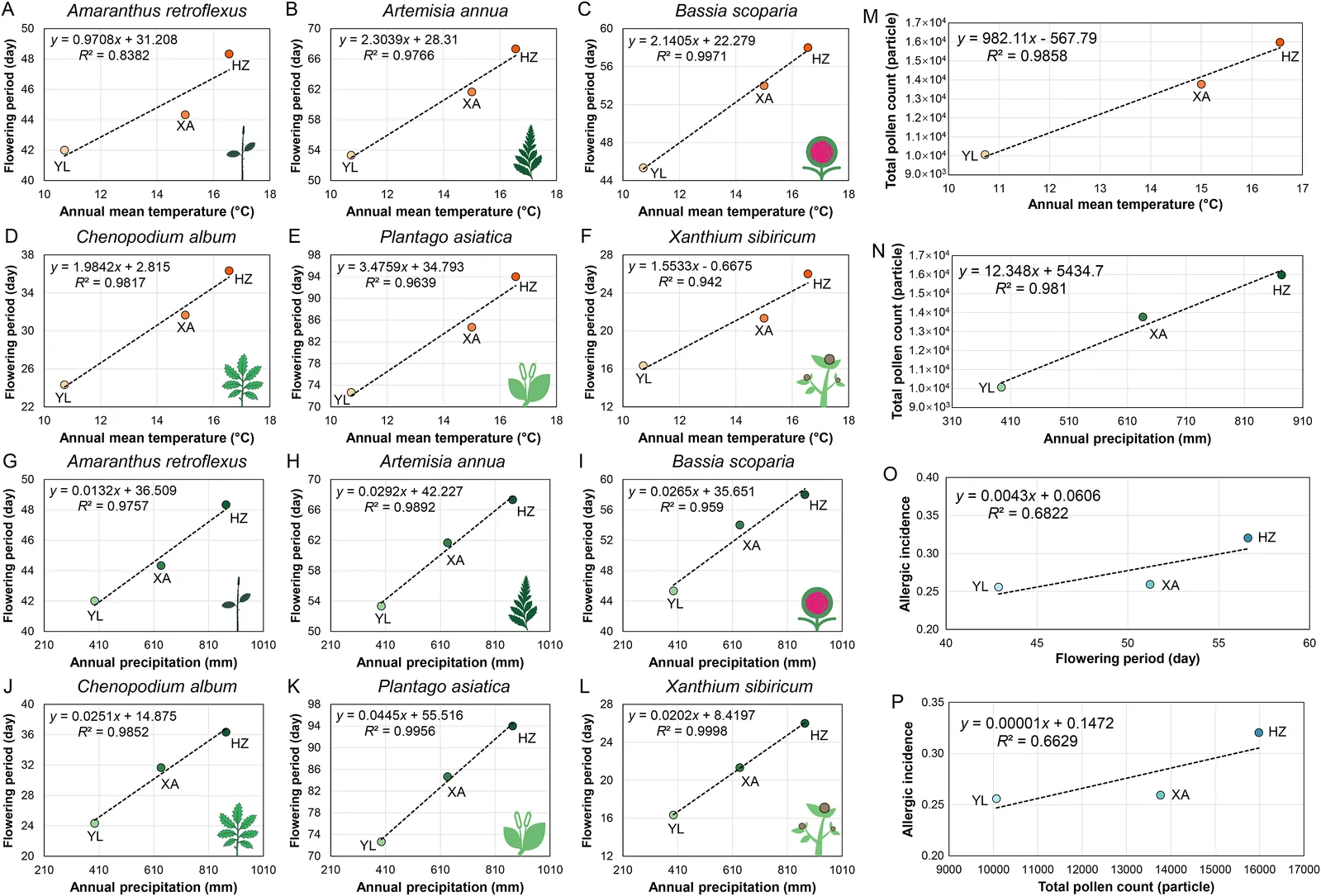
Correlation analysis among annual mean temperature, annual precipitation, flowering period, total pollen count, and clinical allergy cases of key NDAPs in Shaanxi Province, China. (A−F) Annual mean temperature vs. flowering period; (G−L) annual precipitation vs. flowering period; (M) annual mean temperature vs. total pollen count; (N) annual precipitation vs. total pollen count; (O) flowering period vs. adjusted allergic incidence; (P) total pollen count vs. adjusted allergic incidence.

To elucidate the complex ecological and environmental mechanisms responsible for the observed changes in allergenic pollen production, we conducted targeted monitoring in diverse habitats across Shaanxi Province. We selected three representative regions that exemplify diverse climatic conditions: Yulin (mid-temperate zone; temperate continental monsoon climate), Xi'an (warm-temperate zone; warm-temperate semi-arid and semi-humid climate), and Hanzhong (north subtropics; subtropical monsoon climate) (Fig. 4A). Our on-site monitoring results showed significant differences in both monthly and annual average temperatures across these three regions (Fig. 4B and C). Based on this, six key NDAP species (*A. retroflexus*, *A. annua*, *B. scoparia*, *C. album*, *P. asiatica*, *X. sibiricum*) that have large distributions in these regions were selected and subjected to detailed analysis, focusing on their phenological patterns. Specifically, we investigated the flowering period, total pollen count, and concurrent allergy incidence across the three regions. Significant differences in flowering periods were observed among these regions. Overall, higher temperatures and increased precipitation levels were positively correlated with extended flowering durations. For example, Hanzhong, which experiences the highest temperature and precipitation, exhibited the longest flowering period for all six NDAPs (Fig. 4D). Similarly, total pollen production displayed a comparable trend, suggesting implications for allergenic potential. The annual total pollen count in Hanzhong was higher than that in Xi'an and Yulin (*P* = 0.1) (Fig. 4E). Comparatively, the allergy cases in the three monitoring regions revealed that Xi'an had the highest total number of cases, followed by Hanzhong, with Yulin exhibiting the lowest incidence (Fig. 4F). This inconsistency is linked to the population density in the three regions. Upon adjusting for the total population, the calculated incidence rates demonstrated trends that were consistent with those observed in the flowering period and total pollen count (Fig. 4F). Further correlation analysis revealed that both annual mean temperature and annual precipitation exhibited a strong positive correlation (*R*^2^ ≥ 0.8) with the flowering period of the six key NDAPs across the three regions (Fig. 5). Similarly, annual mean temperature and annual precipitation were strongly correlated with total pollen content (*R*^2^ ≥ 0.9). Moreover, strong positive correlations were observed between both the flowering period and the total pollen count and the incidence rate of clinically confirmed allergies (population-adjusted) across the three regions. Collectively, these results indicate that temperature and precipitation influence both the flowering period and pollen count of key NDAPs, thereby contributing to increased pollen-related allergy cases. Based on these findings, we further propose that the increase in allergy cases is attributable to prolonged flowering periods and higher pollen counts, supporting ecological insights into the rising prevalence of pollen allergies in recent years (Fig. S8).

## 4 Discussion

### 4.1 Overview of key findings and research novelty

Our study represents a pioneering effort in generating a comprehensive distribution map of both artificial (APAPs) and wild (NDAPs) allergenic plants across China. Compared with previous studies that primarily focused on qualitative analyses, this study presents the first comprehensive quantification of allergenic plant distributions, particularly the NDAPs that were not reported previously. Building upon species distributions, we integrated multi-dimensional environmental and socio-economic determinants to predict the distribution map of allergy risks and further validated the risk quantification with clinical case reports of pollen allergy. In general, our findings revealed that the spatial distribution of allergenic plants in China exhibited obvious regional heterogeneity, with a high diversity of APAP species in southern and eastern urban agglomerations due to the well-developed urban green space system and diverse landscaping plant species. In northern China, the dominant allergenic plant taxa are represented by herbaceous species, including *Artemisia* and *Ambrosia* species, which are widely distributed in natural and semi-natural habitats and constitute the main sources of airborne allergenic pollen in northern regions, forming a distinct regional characteristic of allergenic plant distribution compared with southern China. These results are consistent with previous studies conducted in other geographic contexts, which similarly found that urbanization and climate conditions significantly influence allergenic plant distributions [6,7].

### 4.2 Distribution patterns and driving factors of APAPs and NDAPs

Specifically, the ecological distribution characteristics of APAPs and NDAPs differ significantly across China. The distribution of APAP species is heavily influenced by socio-economic dynamics; as local economies mature, there is a corresponding increase in green spaces, which facilitates the diversity and abundance of planting species. This increase in green areas correlates with an elevated risk of allergenic reactions among urban populations, a phenomenon observed in previous studies [32,33]. Furthermore, climatic conditions play a key role, as evidenced by the significant differences in both APAP and NDAP between northern and southern regions, with distinct species occupying their respective ecological niches. For APAP distribution, our findings indicated differential responses of specific genera to environmental gradients. For instance, *Ulmus* plants exhibited a predominant distribution in northern China (latitudinal range: 34°N–50°N), correlating with regions characterized by continental climates (annual mean temperature: 5–12 °C; annual precipitation: 400–600 mm). In contrast, *Morus* plants demonstrated higher richness in southern China (latitudinal range: 20°N–34°N), showing a strong association with subtropical monsoon climates (annual mean temperature: 14–24 °C; annual precipitation: 1000–1800 mm). The significant spatial differentiation of APAPs in the two genera along the north-south climatic gradient suggests divergent ecological adaptations to thermal and hydric environmental regimes. Intriguingly, our observations reveal that pine trees are the dominant APAP in nearly all urban centers, aligning with previous studies that highlight the prevalence of *Pinus* in urban landscapes [34]. Regarding other APAPs, the southern and eastern regions are predominantly home to *Populus*, whereas the northern and western regions are primarily characterized by *Cupressus*.

Comparatively, the distribution of NDAP species is largely influenced by climatic factors, with each species occupying a distinct ecological niche, a pattern clearly exemplified by the *Artemisia* genus, a dominant NDAP taxon in northern China that exhibits an overall climatic preference for drier, semi-arid environmental conditions (typically annual mean temperatures of 5–12 °C and annual precipitation of 200–500 mm) [35]. Within this genus, however, significant interspecific differentiation in distribution patterns and climatic adaptability is observed, with distinct species segregating across China in accordance with their specific microclimatic requirements, and this spatial segregation is strongly correlated with the Heihe-Tengchong Line, a key biogeographical demarcation in China. Specifically, *A. desertorum*, *A. dracunculus*, and *A. sieversiana* are primarily distributed to the west of the Heihe-Tengchong Line, where they are adapted to the continental arid and semi-arid climates of this region (annual mean temperature: 3–10 °C; annual precipitation: 100–400 mm), consistent with the genus’s overall preference for dry conditions. In contrast, *A. annua* and *A. caruifolia* are widely distributed to the east of the Heihe-Tengchong Line, having adapted to the warmer and more humid climatic conditions of this area (annual mean temperature: 10–18 °C; annual precipitation: 500–1000 mm). As a notable exception to the genus’s northern distribution trend, *A. argyi* is predominantly confined to southern China, where it thrives under the subtropical monsoon climate (annual mean temperature: 14–20 °C; annual precipitation: 1000–1500 mm). This clear interspecific spatial differentiation within the *Artemisia* genus fully demonstrates that congeneric NDAP species can develop distinct ecological requirements despite shared taxonomic classification, and further highlights the differential responses of allergenic plant species to regional environmental and climatic gradients. Furthermore, other NDAPs such as *X. sibiricum* and *C. album* also exhibit clear climate-driven distribution patterns. *X. sibiricum* shows a preference for temperate regions with moderate rainfall (400–800 mm), while *C. album*, being highly adaptable, spans a wider range of climatic conditions, from arid northwest to humid southeast China, reflecting its broader ecological amplitude. These findings highlight the importance of climate variables in shaping the geographic patterning of NDAPs and their associated allergy risks.

### 4.3 Spatial pattern of pollen allergy risk and methodological advancements

The overall allergy risk distribution map reveals a pattern that parallels, yet diverges from, that of allergenic plants. This divergence arises because our study incorporates socio-economic factors (e.g., human population density) and key environmental determinants (e.g., wind speed), which exert a significant influence on the risk landscape of pollen allergies [9,36,37]. Previous studies have similarly emphasized the role of socio-economic factors in shaping allergy risk profiles. For instance, Murrison et al. demonstrated that urbanization and increased population density correlate with heightened allergy prevalence due to the concentration of allergenic species in urban environments [38]. An additional study by Udalova et al. revealed that socio-economic disparities influence susceptibility to allergies, as lower-income populations often lack access to healthcare and allergen management resources, thus increasing their risk [39]. Our analyses indicate that the regions with the highest risk of pollen allergies include provinces of Gansu, Henan, Hubei, Hebei, Shandong, Zhejiang, Jiangsu, and Guangdong in China, with primary allergenic species being wild taxa like *Artemisia*, *J. chinensis*, and *X. sibiricum*, alongside artificially cultivated species such as *Pinus* and *Salix*. This aligns with findings from Zhang et al., who reported similar distributions of allergenic plants in relation to urban centers [40]. Furthermore, innovations in our methodology, particularly the application of allergy case correction, enabled us to derive a more accurate estimate of the incidence rate of allergies, specifically in regions like Shandong, which has relatively low NDAP distribution suitability but high population density. It is noteworthy that these adjusted results differ from the pollen risk assessments; by accounting for clinical allergy cases, the derived data more closely reflect the actual incidence rates of allergies. This methodological advancement is supported by findings from Ma et al., who emphasized the need for refined models that incorporate demographic variables to enhance the accuracy of allergy risk predictions [41]. Moreover, the inclusion of key environmental determinants like wind speed not only correlates with pollen dispersal patterns but also highlights the complexities of allergy risk in relation to climatic variations [29,30]. By incorporating these multifaceted variables into our analysis, our study provides a more comprehensive understanding of allergy risk profiles, consistent with the broader literature that advocates for integrative approaches in epidemiological investigations of pollen allergies.

### 4.4 Future pollen allergy risk under climate change scenarios

Our results also indicate that, under projected climate change scenarios, the risk of pollen allergies is expected to increase, regardless of whether low- or high-emission trajectories are considered. This trend is consistent with the rising incidence of pollen allergy cases documented in recent years and aligns with global studies indicating that climate change exacerbates the prevalence of allergenic species [9,14]. In the low-emission scenario, the total risk area is projected to expand from 4,037,934 km^2^ (2040) to 4,098,872 km^2^ (2100). By contrast, the high-emissions scenario indicates a more dramatic escalation, with increases from 4,186,788 km^2^ (2040) to 4,913,333 km^2^ (2100). These distribution shifts are primarily driven by the 14 NDAP species under investigation, given the inherent uncertainty in predicting future trends of anthropogenic activities. Specifically, under high-emissions scenarios, eleven NDAP species showed an upward trend, whereas under low-emission scenarios, seven species exhibited similar increases. This observation supports our initial hypothesis that climate change is likely to favor an upward trend in allergenic plant populations, corroborating previous findings reporting analogous trends in temperate regions [42]. The significant disparity between SSP126 and SSP585 outcomes highlights the critical importance of emission mitigation strategies in shaping future allergy burdens. While both scenarios predict increased cumulative risks, the high-emission pathway possibly amplifies the threat through three key mechanisms: (1) greater expansion trend (cumulative area under SSP585 exceeds SSP126 by 20% by 2100), (2) greater species involvement (11 vs. 7 species showing upward trends), and (3) intensified habitat suitability. These differential responses demonstrate the nonlinear relationship between warming magnitudes and ecological impacts. Additionally, it is crucial to recognize that not all allergenic species will respond positively to climate change; in both high and low scenarios, certain species may experience a reduction in their suitable habitats, posing potential threats to biodiversity.

### 4.5 Ecological mechanism underlying allergy risk: evidence from climatic gradient validation

From an ecological perspective, our mechanistic investigation suggests that the continuous increase in allergy cases is positively linked to the prolonged flowering periods of allergenic plants, a phenomenon exacerbated by climate change, alongside the associated increases in pollen production. This relationship is supported by previous studies that established a direct correlation between extended flowering durations and increased pollen exposure. [43]. To further elucidate these dynamics, we implemented a climatic gradient-based strategy, selecting six key wild plants and investigating three distinct regions within Shaanxi Province—Yulin, Xi'an, and Hanzhong, each exhibiting different environmental conditions. Our approach builds upon a previous study that utilized a similar stratification to elucidate the impacts of climate on pollen distribution [44]. Field monitoring results indicate significant disparities in temperature and precipitation across these three locations. Specifically, significant differences in flowering timing were observed among the three sites for all six NDAPs. Hanzhong exhibited the longest flowering duration, followed by Xi'an, with Yulin exhibiting the shortest. Flowering duration demonstrated a positive correlation with annual mean temperature and annual precipitation, with increased warmth and moisture correlated with prolonged flowering periods. Correspondingly, pollen monitoring results corroborated this trend, revealing that total pollen production in these areas followed a similar pattern. In terms of clinical allergy cases recorded during the same timeframe, significant differences emerged among the three locations, with Xi'an exhibiting the highest incidence, followed by Hanzhong and Yulin. This result diverges from the above findings concerning flowering timing and total pollen count, as socio-economic factors (e.g., total urban population) varied across these locations. Although Hanzhong experienced higher temperatures and precipitation, its urban population was lower than that of Xi'an. After correcting for the total human population, this trend is consistent with earlier observations regarding allergenic plants, demonstrating a similar pattern. Collectively, these results strongly indicate that the increasing incidence of allergy cases is closely associated with extended flowering periods and augmented pollen production among allergenic plants, thus providing empirical evidence to elucidate the ecological mechanisms underpinning these trends.

### 4.6 Targeted public health and management implications

Building on our findings, we propose targeted public health interventions to mitigate pollen allergy risks, informed by the spatial and temporal patterns of allergenic plant distributions and their socio-environmental drivers. First, urban planning and green space management in high-risk provincial-level regions (e.g., Jiangsu, Gansu, Shanxi, Shaanxi, Hubei, Hebei, and Guangdong) should prioritize replacing high-risk APAP species (e.g., *Pinus*, *Populus*, and *Platanus*) with low-allergenic alternatives in densely populated areas. For instance, urban forestation programs in Beijing and Shanghai, where APAP diversity peaks, could substitute allergenic *Cupressus* and *Salix* with native flowering shrubs like *Hibiscus* or non-pollen-producing ornamentals. Second, ecological restoration projects in NDAP-dominated regions (e.g., northern China’s *Artemisia* habitats) should incorporate pollen suppression measures, such as strategic mowing before flowering peaks (August–September for *Artemisia*), to reduce airborne pollen loads. Third, early warning systems should be established to monitor provincial-level phenological patterns: spring allergy alerts (March–May) targeting *Populus* and *Ulmus* pollen in northern regions (e.g., Hebei, Liaoning), and autumn alerts (August–October) focusing on *Artemisia* in northwestern regions (e.g., Gansu, Inner Mongolia). Population prioritization should focus on economically developed urban clusters (e.g., Yangtze River Delta, Pearl River Delta) where high APAP density intersects with extreme population density (>1000 persons/km^2^). These regions require enhanced allergenic pollen monitoring networks, particularly downwind of major green spaces, combined with real-time public health advisories through mobile platforms. Rural populations in NDAP expansion zones (e.g., Loess Plateau) need improved access to allergy diagnostics and immunotherapy, as our validation in Shaanxi revealed great increases in outpatient cases despite limited healthcare infrastructure. Finally, climate adaptation strategies must address the projected expansion of high-suitability NDAP habitats under SSP585 by 2100. Provincial-level governments should establish dynamic risk zoning maps that integrate future climate projections, particularly in transitional ecotones like the Qinling Mountains in Shaanxi, where our validation showed northward shifts of *X. sibiricum* and *A. annua* habitats. Cross-departmental collaborations between ecological conservation and public health agencies are critical to balance urban greening benefits with allergy mitigation.

### 4.7 Study limitations and future research directions

Despite the comprehensive insights gained from this study, several key limitations warrant consideration. Specifically, air pollution exerts a critical synergistic effect with pollen exposure to exacerbate respiratory allergic symptoms, especially in economically developed urban clusters with high APAP density and heavy industrial activity. Ambient air pollutants (e.g., PM_2.5_, NO_2_, SO_2_) can damage the respiratory mucosal barrier, increasing the permeability of the airway epithelium and enhancing the penetration of pollen allergens into the circulatory system [45]. Additionally, reactive oxygen species generated by air pollutants can modify pollen protein structures, elevating their allergenicity and triggering stronger immune responses in sensitized individuals [46]. This synergistic interaction highlights an important gap in our current risk assessment framework, which does not fully incorporate air quality parameters. Future studies should integrate real-time air pollution monitoring data and pollen allergen modification assays to develop a more holistic risk prediction model. Moreover, the present study focused primarily on terrestrial allergenic plants, while airborne pollen from aquatic or wetland species was not included; expanding the taxonomic scope of monitored plants could further refine allergy risk mapping. Addressing these limitations will help advance the development of integrated environmental health strategies that simultaneously target pollen exposure reduction and air pollution control, ultimately providing more robust support for public health decision-making.

## 5 Conclusion

In this study, we first revealed the spatiotemporal dynamics of pollen allergenic risks across China, demonstrating a distinct divergence between anthropogenic (APAP) and natural (NDAP) allergenic plant distributions shaped by socio-economic development and climatic gradients. We demonstrate that high-risk zones predominantly cluster in eastern urban centers with intensive artificial plantations (e.g., *Pinus*, *Populus*) and northwestern natural ecosystems dominated by *Artemisia* species, while future climate scenarios project significant habitat expansions of wild allergenic flora, particularly under high-emission (SSP585) trajectories. We further elucidate that the escalating allergy burden is mechanistically linked to climate-driven prolongation of pollen seasons and intensified pollen production, compounded by urbanization patterns. These findings establish a critical scientific foundation for spatially targeted public health strategies and climate-resilient urban planning, emphasizing the urgent need to reconcile ecological conservation with allergy mitigation in China’s ongoing green transition. By integrating multi-dimensional risk drivers from biogeographical patterns to socio-economic exposures, this work provides a valuable reference for predictive environmental health management in the context of global change.

## Data availability statement

The sources of NDAP occurrence data from GBIF database are available in Table S4. The sources of NDAP occurrence data from NPSRC database are openly available at https://www.cvh.ac.cn. The data of current and future global environmental variables are openly available at https://www.worldclim.org. The other data that supports the findings of this study are available in the supporting information material of this article.

## CRediT authorship contribution statement

**Bo Cao:** Writing – review & editing, Writing – original draft, Visualization, Supervision, Resources, Project administration, Conceptualization. **Chengke Bai:** Writing – review & editing, Supervision, Funding acquisition. **Kunyi Wu:** Validation, Resources, Methodology, Investigation. **Ting La:** Visualization, Validation, Resources, Methodology, Investigation. **Nan Xu:** Validation, Resources, Investigation. **Lianjin Liu:** Validation, Resources, Investigation. **Xiaofang Zhou:** Validation, Resources, Investigation. **Chong Chen:** Validation, Resources, Investigation. **Tiantian Bin:** Validation, Resources, Investigation. **Shan Feng:** Validation, Resources, Investigation. **Yali Zhang:** Validation, Resources, Investigation. **Guishuang Li:** Writing – review & editing, Supervision, Methodology.

## Declaration of competing interest

All authors declare that they have no conflict of interest.
