# Supplementary material for: Unraveling the increasing burden of allergies: Spatiotemporal dynamics of pollen allergy risk in China
Source: Eco Environ Health. 2026 Jul 30;5(3):100267. doi: 10.1016/j.eehl.2026.100267 (PMC13560001; doi:10.1016/j.eehl.2026.100267)
Supplement: Supplementary file 1 [file mmc1.docx]

**Supporting information for:**

Unraveling the increasing burden of allergies: Spatiotemporal dynamics of pollen allergy risk in China

Bo Cao^a,b,c,1,*^, Chengke Bai^c,1^, Kunyi Wu^b^, Ting La^d^, Nan Xu^e^, Lianjin Liu^c^, Xiaofang Zhou^c^, Chong Chen^c^, Tiantian Bin^c^, Shan Feng^c^, Yali Zhang^c^, Guishuang Li^c^

^a^ Department of Otolaryngology, The Second Affiliated Hospital of Xi’an Jiaotong University, Xi’an 710004, China

^b^ Core Research Laboratory, The Second Affiliated Hospital of Xi’an Jiaotong University, Xi’an 710004, China

^c^ College of Life Sciences, Shaanxi Normal University, Xi’an 710119, China

^d^ National-Local Joint Engineering Research Center of Biodiagnosis & Biotherapy, The Second Affiliated Hospital of Xi’an Jiaotong University, Xi’an 710004, China

^e^ Department of Clinical Laboratory, The Second Affiliated Hospital of Xi’an Jiaotong University, Xi’an 710004, China

***Corresponding author.**

Email: [bo_cao@xjtu.edu.cn](mailto:bo_cao@xjtu.edu.cn) (Bo Cao)

^1^These authors contributed equally to this work.

**This file includes:**

*Supplementary Figure S1 to S8*

*Supplementary Table S1 to S8*

**Figure S1.** **Study design for spatial distribution of artificial planted allergenic plants (APAPs) in China.**

**Figure S2. Species occurrence records of main naturally distributed allergenic plants (NDAPs) in China.** The species occurrence records for main NDAPs in China were collected using a comprehensive approach including field investigation, literature review, and database search. A total of 16,131 occurrence records of the 14 main NDAP species for subsequently distribution modeling. NDAPs: naturally distributed allergenic plants.

**Figure S3. Weight - risk area response curve for allergic risk analysis.** A gradient-based weight sensitivity analysis was conducted in which the sum of all weights was constrained to 1. The APAP distribution layer and NDAP suitability layer were assigned a fixed weight of 0.3 and 0.3, respectively. Population density and wind speed layers were adjusted in increments of 0.05 while maintaining the unity constraint. For each combination, the area classified as high-risk and very high-risk class was calculated. APAP: artificial planted allergenic plant. NDAP: naturally distributed allergenic plant.

**Figure S4. Spatial distribution pattern of artificial planted** **allergenic plants (APAPs) in China.** The figure shows the spatial distribution of dominant APAPs in China, with eight major genera (*Pinus*, *Populus*, *Salix*, *Platanus*, *Metasequoia*, *Cupressus*, *Ulmus*, *Morus*) dominating urban green spaces. APAP distribution and abundance are positively correlated with regional economic development, with obvious regional differences in genus composition (e.g., *Pinus*, *Populus*, *Salix* dominate in Beijing; *Pinus*, *Cupressus*, *Morus* dominate in Guangdong). APAPs are predominantly distributed in East and South China corresponding to high economic development and favorable hydrothermal conditions.

**Figure S5. ROC/AUC and null-model validation for Maxent modeling of main NDAPs under current condition.** (A) - (N): ROC curves and AUC values of Maxent modeling for 14 main NDAPs. (O): Null-model validation of Maxent modeling. The modeling outcomes demonstrated robust accuracy and reliability with AUC values ranging from 0.836 to 0.950, indicating high predictive performance. ROC: receiver operating characteristic. AUC: area under the curve. NDAPs: naturally distributed allergenic plants.

**Figure S6. Jackknife validation for Maxent modeling of 14 main NDAPs under current condition.** The contribution of different environmental factors on the distribution of each NDAP was shown. NDAPs: naturally distributed allergenic plants.

**Figure S7. Potential suitability distribution of main NDAPs under current condition in China.** The suitability distribution was represented as probability and divided into four classes. Class 1: Low suitability; Class 2: Moderate suitability; Class 3: High suitability; Class 4: Very high suitability. (A): *Amaranthus retroflexus*. (B): *Ambrosia artemisiifolia*. (C): *Artemisia annua*. (D): *Artemisia argyi*. (E): *Artemisia caruifolia*. (F): *Artemisia desertorum*. (G): *Artemisia dracunculus*. (H): *Artemisia sieversiana*. (I): *Bassia scoparia*. (J): *Chenopodium album*. (K): *Humulus scandens*. (L): *Juniperus chinensis*. (M): *Plantago asiatica*. (N): *Xanthium sibiricum*. NDAPs: naturally distributed allergenic plants. The full names for abbreviations of provincial-level regions in China were given in Table 1.

**Figure S8. Proposed ecological mechanism of increasing pollen allergies under climate change.** Based on field and clinical investigations, an ecological mechanism for the increase in pollen allergy cases is proposed in this study. Rising temperatures and precipitation in recent years contribute to: 1) expanded habitat suitability and potential distribution of allergenic plants; and 2) prolonged flowering period along with increased pollen production. These changes result in greater population exposure to pollen allergens, ultimately leading to higher incidence of pollen allergy cases globally.

**Table S1.** Prefecture-level cities of APAP distribution investigation

| **Provincial-level region** | **City/Region** | **Manual survey (M)** | **Image analysis (I)** | **Literature review (L)** |
| --- | --- | --- | --- | --- |
| Anhui | Hefei | M |  | L |
|  | Wuhu |  | I | L |
|  | Bengbu |  | I | L |
|  | Huainan |  | I | L |
|  | Ma’anshan |  | I | L |
|  | Huaibei |  | I | L |
|  | Tongling |  | I | L |
|  | Anqing |  | I | L |
|  | Huangshan |  | I | L |
|  | Chuzhou | M |  | L |
|  | Fuyang | M |  | L |
|  | Suzhou |  | I | L |
|  | Chaohu |  | I | L |
|  | Lu’an |  | I | L |
|  | Bozhou |  | I | L |
|  | Chizhou |  | I | L |
|  | Xuancheng |  | I | L |
| Beijing | Beijing | M |  | L |
| Fujian | Fuzhou | M |  | L |
|  | Xiamen | M |  | L |
|  | Putian |  | I | L |
|  | Sanming |  | I | L |
|  | Quanzhou |  | I | L |
|  | Zhangzhou |  | I | L |
|  | Nanping |  | I | L |
|  | Longyan |  | I | L |
|  | Ningde |  | I | L |
| Chongqing | Chongqing | M |  | L |
| Gansu | Lanzhou | M |  | L |
|  | Jiuquan |  | I | L |
|  | Jinchang |  | I | L |
|  | Baiyin |  | I | L |
|  | Tianshui | M |  | L |
|  | Wuwei |  | I | L |
|  | Zhangye |  | I | L |
|  | Pingliang |  | I | L |
|  | Jiuquan |  | I | L |
|  | Qingyang |  | I | L |
|  | Dingxi |  | I | L |
|  | Longnan |  | I | L |
|  | Linxia |  | I | L |
|  | Gannan |  | I | L |
| Guangdong | Guangzhou | M |  | L |
|  | Shaoguan |  | I | L |
|  | Shenzhen | M |  | L |
|  | Zhuhai | M |  | L |
|  | Shantou |  | I | L |
|  | Foshan | M |  | L |
|  | Jiangmen |  | I | L |
|  | Zhanjiang |  | I | L |
|  | Maoming |  | I | L |
|  | Zhaoqing |  | I | L |
|  | Huizhou |  | I | L |
|  | Meizhou |  | I | L |
|  | Shanwei |  | I | L |
|  | Heyuan |  | I | L |
|  | Yangjiang |  | I | L |
|  | Qingyuan |  | I | L |
|  | Dongguan | M |  | L |
|  | Zhongshan | M |  | L |
|  | Chaozhou |  | I | L |
|  | Jieyang |  | I | L |
|  | Yunfu |  | I | L |
| Guangxi | Nanning | M |  | L |
|  | Liuzhou |  | I | L |
|  | Guilin |  | I | L |
|  | Wuzhou |  | I | L |
|  | Beihai |  | I | L |
|  | Fangchenggang |  | I | L |
|  | Qinzhou |  | I | L |
|  | Guigang |  | I | L |
|  | Yulin |  | I | L |
|  | Baise |  | I | L |
|  | Hezhou |  | I | L |
|  | Hechi |  | I | L |
|  | Laibin |  | I | L |
|  | Chongzuo |  | I | L |
| Guizhou | Guiyang | M |  | L |
|  | Liupanshui |  | I | L |
|  | Zunyi |  | I | L |
|  | Anshun |  | I | L |
|  | Bijie |  | I | L |
|  | Tongren |  | I | L |
|  | Qianxinan |  | I | L |
|  | Qiandongnan |  | I | L |
|  | Qiannan |  | I | L |
| Hainan | Haikou | M |  | L |
|  | Sanya |  | I | L |
| Hebei | Shijiazhuang | M |  | L |
|  | Tangshan |  | I | L |
|  | Qinhuangdao |  | I | L |
|  | Handan |  | I | L |
|  | Xingtai |  | I | L |
|  | Zhangjiakou | M |  | L |
|  | Chengde |  | I | L |
|  | Cangzhou |  | I | L |
|  | Langfang |  | I | L |
|  | Hengshui |  | I | L |
| Henan | Zhengzhou | M |  | L |
|  | Kaifeng |  | I | L |
|  | Luoyang | M |  | L |
|  | Pingdingshan |  | I | L |
|  | Anyang |  | I | L |
|  | Hebi |  | I | L |
|  | Xinxiang |  | I | L |
|  | Jiaozuo |  | I | L |
|  | Puyang |  | I | L |
|  | Xuchang |  | I | L |
|  | Luohe |  | I | L |
|  | Sanmenxia |  | I | L |
|  | Nanyang |  | I | L |
|  | Shangqiu |  | I | L |
|  | Xinyang |  | I | L |
|  | Zhoukou |  | I | L |
|  | Zhumadian |  | I | L |
| Heilongjiang | Harbin |  | I | L |
|  | Qiqihar |  | I | L |
|  | Jixi |  | I | L |
|  | Hegang |  | I | L |
|  | Shuangyashan |  | I | L |
|  | Daqing |  | I | L |
|  | Yichun |  | I | L |
|  | Jiamusi |  | I | L |
|  | Qitaihe |  | I | L |
|  | Mudanjiang |  | I | L |
|  | Heihe |  | I | L |
|  | Suihua |  | I | L |
| Hong Kong | Hong Kong |  | I | L |
| Hubei | Wuhan | M |  | L |
|  | Huangshi |  | I | L |
|  | Shiyan |  | I | L |
|  | Yichang |  | I | L |
|  | Xiangyang |  | I | L |
|  | Ezhou |  | I | L |
|  | Jingmen |  | I | L |
|  | Shizhou |  | I | L |
|  | Xiaogan |  | I | L |
|  | Jingzhou |  | I | L |
|  | Huanggang |  | I | L |
|  | ShiMen |  | I | L |
|  | Suizhou |  | I | L |
|  | Enshi |  | I | L |
| Hunan | Changsha | M |  | L |
|  | Zhuzhou |  | I | L |
|  | Xiangtan |  | I | L |
|  | Hengyang |  | I | L |
|  | Shaoyang |  | I | L |
|  | Yueyang |  | I | L |
|  | Changde |  | I | L |
|  | Zhangjiajie |  | I | L |
|  | Yiyang |  | I | L |
|  | Yongzhou |  | I | L |
|  | Huaihua |  | I | L |
|  | Loudi |  | I | L |
|  | Xiangxi |  | I | L |
| Jilin | Changchun |  | I | L |
|  | Jilin |  | I | L |
|  | Siping |  | I | L |
|  | Liaoyuan |  | I | L |
|  | Tonghua |  | I | L |
|  | Baishan |  | I | L |
|  | Songyuan |  | I | L |
|  | Baicheng |  | I | L |
|  | Yanbian |  | I | L |
| Jiangsu | Nanjing | M |  | L |
|  | Wuxi | M |  | L |
|  | Xuzhou |  | I | L |
|  | Changzhou |  | I | L |
|  | Suzhou | M |  | L |
|  | Nantong |  | I | L |
|  | Lianyungang |  | I | L |
|  | Huai’an |  | I | L |
|  | Yancheng |  | I | L |
|  | Yangzhou |  | I | L |
|  | Zhenjiang |  | I | L |
|  | Taizhou |  | I | L |
|  | Suqian |  | I | L |
| Jiangxi | Nanchang | M |  | L |
|  | Jingdezhen |  | I | L |
|  | Pingxiang |  | I | L |
|  | Jiujiang |  | I | L |
|  | Xinyu |  | I | L |
|  | Yingtan |  | I | L |
|  | Ganzhou |  | I | L |
|  | Ji’an |  | I | L |
|  | Yichun |  | I | L |
|  | Fuzhou |  | I | L |
|  | Shangrao |  | I | L |
| Inner Mongolia | Hohhot |  | I | L |
|  | Baotou |  | I | L |
|  | Wuhai |  | I | L |
|  | Chifeng |  | I | L |
|  | Tongliao |  | I | L |
|  | Ordos |  | I | L |
|  | Hulunbuir |  | I | L |
|  | Bayannur |  | I | L |
|  | Ulanqab |  | I | L |
|  | Xilingol |  | I | L |
|  | Xing’an |  | I | L |
|  | Alxa |  | I | L |
| Liaoning | Shenyang | M |  | L |
|  | Dalian | M |  | L |
|  | Anshan |  | I | L |
|  | Fushun |  | I | L |
|  | Benxi |  | I | L |
|  | Dandong |  | I | L |
|  | Jinzhou |  | I | L |
|  | Yingkou |  | I | L |
|  | Fuxin |  | I | L |
|  | Liaoyang |  | I | L |
|  | Panjin |  | I | L |
|  | Tieling |  | I | L |
|  | Chaoyang |  | I | L |
|  | Huludao |  | I | L |
| Macau | Macau |  | I | L |
| Ningxia | Yinchuan | M |  | L |
|  | Shizuishan |  | I | L |
|  | Wuzhong |  | I | L |
|  | Guyuan |  | I | L |
|  | Zhongwei |  | I | L |
| Qinghai | Xining |  | I | L |
|  | Haidong |  | I | L |
|  | Haibei |  | I | L |
|  | Huangnan |  | I | L |
|  | Golog |  | I | L |
|  | Yushu |  | I | L |
|  | Haixi |  | I | L |
| Shandong | Jin’an | M |  | L |
|  | Qingdao | M |  | L |
|  | Zibo |  | I | L |
|  | Zaozhuang |  | I | L |
|  | Dongying |  | I | L |
|  | Yantai |  | I | L |
|  | Weifang |  | I | L |
|  | Jining |  | I | L |
|  | Tai’an |  | I | L |
|  | Weihai |  | I | L |
|  | Rizhao |  | I | L |
|  | Linyi |  | I | L |
|  | Dezhou |  | I | L |
|  | Liaocheng |  | I | L |
|  | Binzhou |  | I | L |
|  | Heze |  | I | L |
| Shanxi | Taiyuan | M |  | L |
|  | Datong |  | I | L |
|  | Yangquan |  | I | L |
|  | Changzhi |  | I | L |
|  | Jincheng |  | I | L |
|  | Shuozhou |  | I | L |
|  | Jinzhong |  | I | L |
|  | Yuncheng | M |  | L |
|  | Xinzhou |  | I | L |
|  | Linfen | M |  | L |
|  | Lvliang |  | I | L |
| Shaanxi | Xi’an | M |  | L |
|  | Tongchuan | M |  | L |
|  | Baoji | M |  | L |
|  | Xianyang | M |  | L |
|  | Weinan | M |  | L |
|  | Yan’an | M |  | L |
|  | Yulin | M |  | L |
|  | Hanzhong | M |  | L |
|  | Ankang | M |  | L |
|  | Shangluo | M |  | L |
| Shanghai | Shanghai | M |  | L |
| Sichuan | Chengdu | M |  | L |
|  | Zizhou |  | I | L |
|  | Panzhihua |  | I | L |
|  | Luzhou |  | I | L |
|  | Deyang |  | I | L |
|  | Mianyang |  | I | L |
|  | Guangyuan |  | I | L |
|  | Suining |  | I | L |
|  | Neijiang |  | I | L |
|  | Leshan |  | I | L |
|  | Nanchong |  | I | L |
|  | Meishan |  | I | L |
|  | Yibin |  | I | L |
|  | Guang’an | M |  | L |
|  | Dazhou |  | I | L |
|  | Ya’an |  | I | L |
|  | Bazhong |  | I | L |
|  | Ziyang |  | I | L |
|  | Aba |  | I | L |
|  | Garze |  | I | L |
|  | Liangshan |  | I | L |
| Taiwan | Taipei |  | I | L |
|  | New Taipei |  | I | L |
|  | Taoyuan |  | I | L |
|  | Taichung |  | I | L |
|  | Tainan |  | I | L |
|  | Kaohsiung |  | I | L |
|  | Keelung |  | I | L |
|  | Hsinchu |  | I | L |
|  | Chiayi |  | I | L |
| Tianjin | Tianjin | M |  | L |
| Xizang | Lhasa |  | I | L |
|  | Chamdo |  | I | L |
|  | Nyingchi |  | I | L |
|  | Shannan |  | I | L |
|  | Nagqu |  | I | L |
|  | Ali |  | I | L |
|  | Shigatse |  | I | L |
| Yunnan | Kunming | M |  | L |
|  | Qujing |  | I | L |
|  | Yuxi |  | I | L |
|  | Baoshan |  | I | L |
|  | Zhaotong |  | I | L |
|  | Lijiang |  | I | L |
|  | Pu’er |  | I | L |
|  | Lincang |  | I | L |
|  | Chuxiong |  | I | L |
|  | Honghe |  | I | L |
|  | Wenshan |  | I | L |
|  | Xishuangbanna |  | I | L |
|  | Dali |  | I | L |
|  | Dehong |  | I | L |
|  | Nujiang |  | I | L |
|  | Diqing |  | I | L |
| Xinjiang | Urumqi |  | I | L |
|  | Karamay |  | I | L |
|  | Turpan |  | I | L |
|  | Hami |  | I | L |
| Zhejiang | Hangzhou | M |  | L |
|  | Ningbo |  | I | L |
|  | Wenzhou | M |  | L |
|  | Jiaxing |  | I | L |
|  | Huzhou |  | I | L |
|  | Shaoxing |  | I | L |
|  | Jinhua |  | I | L |
|  | Quzhou |  | I | L |
|  | Zhoushan |  | I | L |
|  | Taizhou |  | I | L |
|  | Lishui |  | I | L |

**Table S2.** References for artificial planted allergenic plant (APAP) occurrence records in China

The detailed version of Table S2 can be found at the end of the supporting information.

**Table S3.** Main NDAP species and their allergenicity in this study

| **NDAP species**  **(common name)** | **Allergen** | **Biochemical name** | **Allergen exposure route** |
| --- | --- | --- | --- |
| *Amaranthus retroflexus*  (Redroot pigweed) * | Ama r 1 | Ole e 1- like protein | Airway |
|  | Ama r 2 | Profilin | Airway |
| *Ambrosia artemisiifolia*  (Short ragweed) * | Amb a 1 | Pectate lyase | Airway |
|  | Amb a 3 | Plastocyanine | Airway |
|  | Amb a 12 | Enolase | Airway |
| *Artemisia annua*  (Sweet wormwood) * | Art an 2 | Pathogenesis related protein 1 | Airway |
|  | Art an 4 | Profilin | Airway |
|  | Art an 14 | Fructose bisphosphate aldolase | Airway |
| *Artemisia argyi*  (Silvery wormwood) * | Art ar 1 | Defensin-like protein | Airway |
|  | Art ar 2 | PR-1 | Airway |
|  | Art ar 3 | Non-specific lipid transfer protein type 1 | Airway |
| *Artemisia caruifolia*  (Green wormwood) ^[1]^ | Art a 1 | Unknown | Airway |
| *Artemisia desertorum*  (Desert wormwood) ^[2]^ | Art d 2 | Unknown | Airway |
| *Artemisia dracunculus*  (Tarragon) ^[3]^ | Unknown | Unknown | Airway |
| *Artemisia sieversiana*  (Sieversian wormwood) * | Art si 1 | Defensin-like protein | Airway |
|  | Art si 2 | Pathogenesis related protein 1 | Airway |
|  | Art si 14 | Fructose bisphosphate aldolase | Airway |
| *Bassia scoparia*  (Burning bush) * | Koc s 1 | Ole e 1- like protein | Airway |
|  | Koc s 2 | Profilin | Airway |
| *Chenopodium album*  (Lamb's quarters) * | Che a 1 | Ole e 1 homologue | Airway |
|  | Che a 2 | Profilin | Airway |
|  | Che a 3 | Polcalcin | Airway |
| *Humulus scandens*  (Japanese hop) ^[4,5]^ | Hum j 1 | Unknown | Airway |
|  | Hum s 1 | Unknown | Airway |
| *Juniperus chinensis*  (Chinese juniper) ^[6]^ | Sab c 1 | Unknown | Airway |
| *Plantago asiatica*  (Asian plantain) ^[7]^ | Unknown | Unknown | Airway |
| *Xanthium sibiricum*  (Cang Er Zi) ^[8]^ | Unknown | Unknown | Airway |

*Note*: The allergenicity of NDAP species with asterisk (*) was referred to the ALLERGEN NOMENCLATURE database (<https://www.allergen.org/>). The allergenicity of NDAP species with numerical superscripts was referred to references as following. NDAP: naturally distributed allergenic plant.

References:

[1]	He Y, Liu Z, Gao B, et al. Cloning, expression and immunocharacterization of *Artemisia apiacea Hance* allergen, *Art a1*. *Chinese Journal of Microbiology and Immunology*, 2005, 25: 768-772.

[2] Artemisia desertorum pollen allergen Art d 2 mRNA, complete cds. <https://www.ncbi.nlm.nih.gov/nuccore/1682103807>

[3] Dragon Wormwood (Artemisia dracunculus). <https://www.pollenlibrary.com/Specie/Artemisia+dracunculus/>

[4] Yin X, Gu X, Li Z, et al. Recombinant expression, purification and identification of *Humulus Japonicus* pollen allergen *Humj1*. *Journal of Changchun University of Science and Technology*, 2024, 47: 101-106.

[5] Liu J, Sun X, Xu J, et al. Expression, purification and structure prediction of *Hum s1*, a major allergen of *Humulus Scandens*. *Journal of Sichuan University*, 2016, 47: 14-18.

[6] Sab c 1 allergen [*Juniperus chinensis*]. <https://www.ncbi.nlm.nih.gov/protein/AVW83019.1>

[7] Sun X, Li Y. Study on allergens of *Plantago asiatica* pollen in the city of Xi'an. *Chinese Journal of Public Health*, 1996, 6: 1.

[8] Zhang H, Zhang M, Zhao L, et al. Analysis of inhaled allergen characteristics of adult patients with allergic asthma in Beijing. *Journal of New Medicine*, 2024, 55: 787-793.

**Table S4.** Occurrence records of naturally distributed allergenic plants (NDAPs) from GBIF database

| **NDAP species** | **GBIF occurrence record link** |
| --- | --- |
| *Ambrosia artemisiifolia* | <https://doi.org/10.15468/dl.ygkrvv> |
| *Artemisia annua* | <https://doi.org/10.15468/dl.72ctf7> |
| *Artemisia argyi* | <https://doi.org/10.15468/dl.xunmnk> |
| *Artemisia caruifolia* | <https://doi.org/10.15468/dl.sjdxwg> |
| *Artemisia desertorum* | <https://doi.org/10.15468/dl.m6g9d5> |
| *Artemisia dracunculus* | <https://doi.org/10.15468/dl.c669pz> |
| *Artemisia sieversiana* | <https://doi.org/10.15468/dl.fpvhxt> |
| *Bassia scoparia* | <https://doi.org/10.15468/dl.sdtzwc> |
| *Chenopodium album* | <https://doi.org/10.15468/dl.jrsvsx> |
| *Humulus scandens* | <https://doi.org/10.15468/dl.j25pak> |
| *Juniperus chinensis* | <https://doi.org/10.15468/dl.396enx> |
| *Plantago asiatica* | <https://doi.org/10.15468/dl.979dwm> |
| *Xanthium sibiricum* | <https://doi.org/10.15468/dl.s854yb> |

**Table S5.** Weight sensitivity analysis of pollen allergy risk

| **APAP/NDAP distribution weight** | **Human population density weight** | **Wind speed weight** | **Area of high-risk region (10,000 km^2^)** |
| --- | --- | --- | --- |
| 0.6 | 0.05 | 0.35 | 181.6897 |
| 0.6 | 0.1 | 0.3 | 197.7105 |
| 0.6 | 0.15 | 0.25 | 210.2175 |
| 0.6 | 0.2 | 0.2 | 225.1620 |
| 0.6 | 0.25 | 0.15 | 240.9658 |
| 0.6 | 0.3 | 0.1 | 258.3272 |
| 0.6 | 0.35 | 0.05 | 232.7386 |

*Note*: Area of high-risk region for each weight gradient combination was calculated as regions of [high risk + very high risk] classes in China.

**Table S6.** Main APAP species and estimated distribution area by provincial-level region in China

| **Provincial-level region** | **Main APAP species** | **Estimated distribution area (km^2^)** |
| --- | --- | --- |
| Anhui | *Pinus, Metasequoia, Cupressus, Populus, Salix, Platanus* | 1014 |
| Beijing | *Pinus, Populus, Salix, Platanus, Morus, Cupressus, Ulmus, Metasequoia* | 486 |
| Chongqing | *Pinus, Salix, Cupressus, Populus* | 824 |
| Fujian | *Pinus, Morus, Metasequoia* | 981 |
| Gansu | *Pinus, Salix, Cupressus, Populus* | 738 |
| Guangdong | *Pinus, Cupressus, Morus, Salix, Ulmus, Metasequoia* | 4236 |
| Guangxi | *Pinus, Morus, Ulmus, Cupressus* | 898 |
| Guizhou | *Pinus, Morus, Cupressus* | 768 |
| Hainan | *Pinus, Salix* | 473 |
| Hebei | *Pinus, Platanus, Cupressus, Populus* | 1236 |
| Heilongjiang | *Pinus, Salix, Ulmus, Populus* | 814 |
| Henan | *Pinus, Salix, Platanus, Populus* | 1500 |
| Hong Kong | *Pinus, Cupressus, Morus, Salix, Ulmus, Metasequoia* | 403 |
| Hubei | *Pinus, Populus, Salix, Metasequoia, Cupressus, Platanus* | 1316 |
| Hunan | *Pinus, Populus, Platanus, Metasequoia, Ulmus* | 1245 |
| Inner Mongolia | *Pinus, Populus, Salix, Cupressus, Ulmus* | 719 |
| Jiangsu | *Pinus, Cupressus, Metasequoia, Platanus, Salix, Populus, Morus* | 2350 |
| Jiangxi | *Pinus, Morus, Salix, Platanus, Ulmus, Cupressus, Populus, Metasequoia* | 795 |
| Jilin | *Pinus, Salix, Ulmus, Populus* | 741 |
| Liaoning | *Pinus, Salix, Cupressus, Populus* | 1082 |
| Macau | *Pinus, Cupressus, Morus, Salix, Ulmus, Metasequoia* | 6 |
| Ningxia | *Pinus, Salix, Cupressus, Populus* | 613 |
| Qinghai | *Pinus, Salix, Cupressus, Populus* | 677 |
| Shaanxi | *Pinus, Populus, Salix, Platanus, Metasequoia, Cupressus* | 934 |
| Shandong | *Pinus, Populus, Salix, Ulmus, Cupressus, Platanus* | 1947 |
| Shanghai | *Platanus, Pinus, Populus, Morus, Ulmus, Cupressus, Salix, Metasequoia* | 617 |
| Shanxi | *Pinus, Salix, Ulmus, Populus* | 683 |
| Sichuan | *Pinus, Populus, Salix, Cupressus, Ulmus, Morus* | 1378 |
| Taiwan | *Pinus, Morus, Cupressus, Metasequoia* | 8 |
| Tianjin | *Pinus, Salix, Platanus, Populus* | 459 |
| Xizang | *Pinus, Cupressus* | 559 |
| Xinjiang | *Pinus, Salix, Populus* | 697 |
| Yunnan | *Pinus, Salix, Cupressus, Metasequoia* | 1114 |
| Zhejiang | *Pinus, Salix, Metasequoia, Platanus, Populus, Cupressus, Morus* | 1068 |

*Note*: Data were collected in urban centers of prefecture-level cities in each provincial-level region. APAP: artificial planted allergenic plant.

**Table S7.** Area of potential distribution of main NDAPs in China

| **NDAP species** | **High**  **(10,000 km^2^)** | **Very high**  **(10,000 km^2^)** | **Total [high+very high]**  **(10,000 km^2^)** |
| --- | --- | --- | --- |
| *Amaranthus retroflexus* | 171.1163 | 82.7292 | 253.8455 |
| *Ambrosia artemisiifolia* | 92.0156 | 51.1424 | 143.1580 |
| *Artemisia annua* | 211.9826 | 124.3559 | 336.3385 |
| *Artemisia argyi* | 146.8646 | 75.3420 | 222.2066 |
| *Artemisia caruifolia* | 77.6962 | 50.4913 | 128.1875 |
| *Artemisia desertorum* | 61.8264 | 30.7934 | 92.6198 |
| *Artemisia dracunculus* | 103.5365 | 51.8003 | 155.3368 |
| *Artemisia sieversiana* | 185.2865 | 91.2152 | 276.5017 |
| *Bassia scoparia* | 231.3437 | 114.8490 | 346.1927 |
| *Chenopodium album* | 247.9323 | 166.9323 | 414.8646 |
| *Humulus scandens* | 184.7031 | 72.0521 | 256.7552 |
| *Juniperus chinensis* | 122.2378 | 77.4931 | 199.7309 |
| *Plantago asiatica* | 208.8194 | 156.3455 | 365.1649 |
| *Xanthium sibiricum* | 216.4792 | 121.9184 | 338.3976 |

*Note*: NDAP: naturally distributed allergenic plants.

**Table S8.** Area of distribution shifts of main NDAPs under climate change in China

| **Climate scenario** | **High**  **(10,000 km^2^)** | **Very high**  **(10,000 km^2^)** | **Total [high+very high]**  **(10,000 km^2^)** |
| --- | --- | --- | --- |
| **SSP126** |  |  |  |
| 2040 | 321.6753 | 82.1181 | 403.7934 |
| 2060 | 307.4948 | 96.2396 | 403.7344 |
| 2080 | 295.7986 | 109.2656 | 405.0642 |
| 2100 | 284.5295 | 125.3577 | 409.8872 |
| **SSP585** |  |  |  |
| 2040 | 338.5851 | 80.0937 | 418.6788 |
| 2060 | 306.4566 | 134.2899 | 440.7465 |
| 2080 | 306.8438 | 148.4010 | 455.2448 |
| 2100 | 304.2639 | 187.0694 | 491.3333 |

*Note*: NDAP: naturally distributed allergenic plant. SSP: shared socioeconomic pathway.

**Table S2.** References for artificial planted allergenic plant (APAP) occurrence records in China

1. An, Y., Ouyang, Y., & Zhang, L. (2023). Impact of airborne pollen concentration and meteorological factors on the number of outpatients with allergic rhinitis. World Allergy Organ J, 16(4), 100762. doi:10.1016/j.waojou.2023.100762
2. Bai, Q., Zhang, X., Wang, W., Zhang, H., & He, S. (2023). [Detection and analysis of TLR2 expression on blood monocytes and B cells in patients with allergic rhinitis and asthma]. Xi Bao Yu Fen Zi Mian Yi Xue Za Zhi, 39(3), 193-198.
3. Bao, J., Tian, L., Meng, Y., Wu, B., Wang, J., He, J., . . . Zhang, L. (2022). Total IgE in tears accurately reflects the severity and predicts the prognosis of seasonal allergic conjunctivitis. Clin Transl Allergy, 12(3), e12139. doi:10.1002/clt2.12139
4. Bian, Y., Zhu, Y., Guo, S., Wang, J., Xue, Z., Fan, X., & Zhang, J. (2009). [Studies on the correlation of 489 cases of bronchial asthma complicated with allergic rhinitis in Datong]. Lin Chuang Er Bi Yan Hou Tou Jing Wai Ke Za Zhi, 23(2), 67-69.
5. Bishan, C., Bing, L., Chixin, C., Junxia, S., Shulin, Z., Cailang, L., . . . Chuanxiu, L. (2020). Relationship between airborne pollen assemblages and major meteorological parameters in Zhanjiang, South China. PLoS ONE, 15(10), e0240160. doi:10.1371/journal.pone.0240160
6. Buzan, M. R., Zbîrcea, L. E., Gattinger, P., Babaev, E., Stolz, F., Valenta, R., . . . Chen, K. W. (2022). Complex IgE sensitization patterns in ragweed allergic patients: Implications for diagnosis and specific immunotherapy. Clin Transl Allergy, 12(7), e12179. doi:10.1002/clt2.12179
7. Cao, Y., Feng, Y., Wang, Q., & Han, S. (2023). [Analysis of adherence and determinants of sublingual immunotherapy in patients with Artemisia pollen-induced allergic rhinitis]. Lin Chuang Er Bi Yan Hou Tou Jing Wai Ke Za Zhi, 37(6), 448-452. doi:10.13201/j.issn.2096-7993.2023.06.008
8. Cao, Y., Shao, L., Wang, L., Su, K., Zhang, D., Xie, Y., . . . Guo, Y. (2023). Heat shock cognate 70 protein like-2 protein in camphor pollen is one of the major culprits of asthma. Mol Immunol, 156, 170-176. doi:10.1016/j.molimm.2023.01.006
9. Cao, Y. J., Xu, R., Tao, X. Y., Shi, J. B., & Chen, F. H. (2019). [Investigation of temporal and spatial characteristics of allergic rhinitis epidemics by Baidu Index in China]. Zhonghua Er Bi Yan Hou Tou Jing Wai Ke Za Zhi, 54(12), 888-893. doi:10.3760/cma.j.issn.1673-0860.2019.12.002
10. Cao, Z. Z., Xiang, H. J., Gao, J. J., Huang, S. Y., Zheng, B., Zhan, X., . . . Chen, B. B. (2018). [Prevalence of allergy in children with acute rhinosinusitis]. Lin Chuang Er Bi Yan Hou Tou Jing Wai Ke Za Zhi, 32(18), 1377-1382. doi:10.13201/j.issn.1001-1781.2018.18.004
11. Celedón, J. C., Palmer, L. J., Weiss, S. T., Wang, B., Fang, Z., & Xu, X. (2001). Asthma, rhinitis, and skin test reactivity to aeroallergens in families of asthmatic subjects in Anqing, China. Am J Respir Crit Care Med, 163(5), 1108-1112. doi:10.1164/ajrccm.163.5.2005086
12. Chang, C. C., Lee, I. M., Tsai, S. S., & Yang, C. Y. (2006). Correlation of Asian dust storm events with daily clinic visits for allergic rhinitis in Taipei, Taiwan. J Toxicol Environ Health A, 69(3-4), 229-235. doi:10.1080/15287390500227415
13. Chang, Z. N., Chi, C. W., Sun, H. F., Tsai, L. C., Lee, D. T., & Han, S. H. (1991). A unique human IgE-binding epitope on the Bermuda grass pollen recognized by mouse lambda-type monoclonal antibodies. Clin Exp Allergy, 21(4), 503-509. doi:10.1111/j.1365-2222.1991.tb01692.x
14. Chang, Z. N., Liu, C. C., Tam, M. F., Peng, H. J., Tsai, J. J., & Han, S. H. (1995). Characterization of the isoforms of the group I allergen of Cynodon dactylon. J Allergy Clin Immunol, 95(6), 1206-1214. doi:10.1016/s0091-6749(95)70077-3
15. Chang, Z. N., Peng, H. J., Lee, W. C., Chen, T. S., Chua, K. Y., Tsai, L. C., . . . Han, S. H. (1999). Sequence polymorphism of the group 1 allergen of Bermuda grass pollen. Clin Exp Allergy, 29(4), 488-496. doi:10.1046/j.1365-2222.1999.00523.x
16. Chang, Z. N., Tam, M. F., Liu, C. C., Chi, C. W., Peng, H. J., & Han, S. H. (1997). Using monoclonal antibodies to characterize a sequential epitope on the group I allergen of Bermuda grass pollen. Int Arch Allergy Immunol, 114(3), 258-264. doi:10.1159/000237677
17. Chang, Z. N., Tsai, L. C., Chi, C. W., Wang, M. C., Shen, H. D., Lee, D. T., & Han, S. H. (1991). Analysis of allergenic components of Bermuda grass pollen by monoclonal antibodies. Allergy, 46(7), 520-528. doi:10.1111/j.1398-9995.1991.tb00615.x
18. Chen, H., Jiang, Q., Yang, Y., Zhang, W., Yang, L., & Zhu, R. (2022). Cross-Reacting Carbohydrate Determinants Inhibitor Can Improve the Diagnostic Accuracy in Pollen and Food Allergy. J Asthma Allergy, 15, 713-725. doi:10.2147/jaa.S363206
19. Chen, H., Lou, H., Wang, Y., Cao, F., Zhang, L., & Wang, C. (2018). Comparison of the efficacy and mechanisms of intranasal budesonide, montelukast, and their combination in treatment of patients with seasonal allergic rhinitis. Int Forum Allergy Rhinol, 8(11), 1242-1252. doi:10.1002/alr.22197
20. Chen, H., Zhang, L., Lou, H., Wang, Y., Cao, F., & Wang, C. (2021). A Randomized Trial of Comparing a Combination of Montelukast and Budesonide With Budesonide in Allergic Rhinitis. Laryngoscope, 131(4), E1054-e1061. doi:10.1002/lary.28433
21. Chen, J., Kong, W., Xiang, J., Shu, H., Shi, Q., Tan, H., & Zhou, Y. (2008). [Age features of the allergens in allergic rhinitis patients of different age in Hubei area]. Lin Chuang Er Bi Yan Hou Tou Jing Wai Ke Za Zhi, 22(15), 683-685, 694.
22. Chen, J., Kong, W., Zhou, Y., Xiang, J., Shu, H., Shi, Q., & Tan, H. (2007). [The expression of serum IL-10,12,13,16 in patients with allergic rhinitis and vasomotor rhinitis]. Lin Chuang Er Bi Yan Hou Tou Jing Wai Ke Za Zhi, 21(20), 913-915.
23. Chen, K., Liao, Y. F., & Zhang, J. T. (1988). The major aeroallergens in Guangxi, China. Clin Allergy, 18(6), 589-596. doi:10.1111/j.1365-2222.1988.tb02910.x
24. Chen, K., & Zhang, J. T. (1985). A study of aeroallergens in Nanning, Guangxi, China. Ann Allergy, 54(3), 242-244.
25. Chen, K. B., Ji, J., Li, H., Chen, X. W., & Ding, X. (2021). [Effect of daily average temperature on the incidence of allergic rhinitis in Lanzhou]. Zhonghua Er Bi Yan Hou Tou Jing Wai Ke Za Zhi, 56(12), 1300-1306. doi:10.3760/cma.j.cn115330-20210330-00158
26. Chen, L., Lei, L., Cai, Y., & Li, T. (2020). Specific sublingual immunotherapy in children with perennial rhinitis: a systemic review and meta-analysis. Int Forum Allergy Rhinol, 10(11), 1226-1235. doi:10.1002/alr.22589
27. Chen, M., Fan, Z., Zheng, X., Cao, F., & Wang, L. (2016). Risk Factors of Acoustic Neuroma: Systematic Review and Meta-Analysis. Yonsei Med J, 57(3), 776-783. doi:10.3349/ymj.2016.57.3.776
28. Chen, Q. Y., Li, L., Zhang, L., Mo, J. H., Yang, Z. F., Wei, X. L., . . . Xie, P. F. (2018). Efficacy of indoor air purification in treating Artemisia (mugwort) pollen allergic rhinitis: study protocol for a randomised controlled trial. BMC Public Health, 18(1), 841. doi:10.1186/s12889-018-5678-0
29. Chen, R., Zheng, D., Zhang, Y., & Sima, G. (2022). Efficacy and safety of twice-daily olopatadine-mometasone combination nasal spray (GSP301) in the treatment of allergic rhinitis: a systematic review and meta-analysis. Eur Arch Otorhinolaryngol, 279(4), 1691-1699. doi:10.1007/s00405-021-07085-w
30. Chen, R. X., Lu, W. M., Zhu, L. P., Lu, M. P., Wang, M. L., Wang, Y. L., . . . Cheng, L. (2014). Association study on ADAM33 polymorphisms in mite-sensitized persistent allergic rhinitis in a Chinese population. PLoS ONE, 9(4), e95033. doi:10.1371/journal.pone.0095033
31. Chen, S., & Liu, J. (2023). Associations between endometriosis and allergy-related diseases as well as different specific immunoglobulin E allergy: A cross-sectional study. J Obstet Gynaecol Res, 49(2), 665-674. doi:10.1111/jog.15506
32. Chen, T., Norback, D., Deng, Q., Huang, C., Qian, H., Zhang, X., . . . Zhao, Z. (2022). Maternal exposure to PM(2.5)/BC during pregnancy predisposes children to allergic rhinitis which varies by regions and exclusive breastfeeding. Environ Int, 165, 107315. doi:10.1016/j.envint.2022.107315
33. Chen, X., Deng, C., Mi, J., Chen, M., Li, Y., Liu, Z., & Feng, S. (2020). Barrier Protection Measures for the Management of Allergic Rhinitis: A Systematic Review and Meta-analysis. Am J Rhinol Allergy, 34(4), 564-572. doi:10.1177/1945892420912370
34. Chen, X., Deng, R., Chi, W., Hua, X., Lu, F., Bian, F., . . . Li, D. Q. (2019). IL-27 signaling deficiency develops Th17-enhanced Th2-dominant inflammation in murine allergic conjunctivitis model. Allergy, 74(5), 910-921. doi:10.1111/all.13691
35. Chen, Y., Han, L., Zhou, Y., Yang, L., & Guo, Y. S. (2020). Artemisia Pollen Extracts Exposed to Diesel Exhaust Enhance Airway Inflammation and Immunological Imbalance in Asthmatic Mice Model. Int Arch Allergy Immunol, 181(5), 342-352. doi:10.1159/000505747
36. Chen, Y., Wong, G. W., & Li, J. (2016). Environmental Exposure and Genetic Predisposition as Risk Factors for Asthma in China. Allergy Asthma Immunol Res, 8(2), 92-100. doi:10.4168/aair.2016.8.2.92
37. Chen, Y. L., Shi, H. Y., Ma, T. T., & Wang, X. Y. (2020). Investigation of self-reported chronic urticaria among adults in grasslands of northern China. Medicine (Baltimore), 99(47), e23301. doi:10.1097/md.0000000000023301
38. Chen, Y. L., Wang, X. Y., Wang, H. T., & Wang, X. Y. (2021). [Analysis of airborne allergens in chronic urticaria with airway allergic diseases]. Zhonghua Yu Fang Yi Xue Za Zhi, 55(8), 1011-1015. doi:10.3760/cma.j.cn112150-20210225-00189
39. Chen, Z., Yang, Y., Chen, X., Wu, Z., & Li, S. (2015). Characterization of two pollen allergens of the London plane tree in Shanghai. Iran J Allergy Asthma Immunol, 14(2), 139-148.
40. Chen, Z., Zhu, N., Chen, X., Yang, Y., Li, Y., Wu, Z., & Chen, S. (2013). Purification and identification of 72 kDa and 15 kDa allergens from Broussonetia papyrifera pollen. Iran J Allergy Asthma Immunol, 12(4), 312-320.
41. Chen, Z. X. (2007). [Clinical observation on acupuncture for treatment of allergic rhinitis]. Zhongguo Zhen Jiu, 27(8), 578-580.
42. Cheng, J., Yang, X. N., Liu, X., & Zhang, S. P. (2006). Capsaicin for allergic rhinitis in adults. Cochrane Database Syst Rev, 2006(2), Cd004460. doi:10.1002/14651858.CD004460.pub2
43. Cheng, L., Hirota, T., Enomoto, T., Tamari, M., Akahoshi, M., Matsuda, A., . . . Shirakawa, T. (2006). Lack of association between the IL13 variant Arg110Gln and susceptibility to cedar pollinosis in a Japanese population. Int Arch Allergy Immunol, 139(1), 25-30. doi:10.1159/000089519
44. Cheng, S., Ruan, B., Yu, Y., & Pu, J. (2015). [Relationship among allergic rhinitis, regional pollens and meteorological conditions in Kunming area]. Zhonghua Er Bi Yan Hou Tou Jing Wai Ke Za Zhi, 50(7), 560-563.
45. Cheng, Y. L., Xu, Z. Q., Wang, H., Zhu, D. X., Zhu, Y., Sun, J. L., & Wei, J. F. (2023). Molecular and immunological characterization of two polcalcins as novel allergens of Artemisia sieversiana pollen. Allergol Int, 72(2), 347-350. doi:10.1016/j.alit.2022.10.006
46. Chiang, L. C., Chen, Y. H., Hsueh, K. C., & Huang, J. L. (2007). Prevalence and severity of symptoms of asthma, allergic rhinitis, and eczema in 10- to 15-year-old schoolchildren in central Taiwan. Asian Pac J Allergy Immunol, 25(1), 1-5.
47. Cui, L., & Yin, J. (2019). Association of serum specific IgE levels with asthma in autumn pollen-induced allergic rhinitis: A retrospective analysis. J Asthma, 56(5), 505-511. doi:10.1080/02770903.2018.1466316
48. Cui, X., Tian, W., Wang, W., Li, J., Wang, Z., & Li, C. (2023). Sequence Analysis and Biochemical Characteristics of Two Non-specific Lipid Transfer Proteins from Tartary Buckwheat Seeds. Protein Pept Lett, 30(6), 520-529. doi:10.2174/0929866530666230511154511
49. Cui, X. Y., Chen, X., Yu, C. J., Yang, J., Lin, Z. P., Yin, M., & Cheng, L. (2015). Increased expression of toll-like receptors 2 and 4 and related cytokines in persistent allergic rhinitis. Otolaryngol Head Neck Surg, 152(2), 233-238. doi:10.1177/0194599814562173
50. Cui, Z., Zhang, H., Liu, Y., Yang, Y., & Xiang, Y. (2011). [Analysis of HLA-DQB1 polymorphism for patients with allergic rhinitis of Uygur and Han people in Xinjiang]. Lin Chuang Er Bi Yan Hou Tou Jing Wai Ke Za Zhi, 25(14), 645-648.
51. Dai, Y., Ni, S., Wu, F., & Zhao, X. (2018). Glucocorticoid-Induced Transcription Factor 1 (GLCCI1) Variant Impacts the Short-Term Response to Intranasal Corticosteroids in Chinese Han Patients with Seasonal Allergic Rhinitis. Med Sci Monit, 24, 4691-4697. doi:10.12659/msm.908814
52. Deng, S., & Yin, J. (2019). Clinical utility of basophil activation test in diagnosis and predicting severity of mugwort pollen-related peach allergy. World Allergy Organ J, 12(6), 100043. doi:10.1016/j.waojou.2019.100043
53. Deng, S., & Yin, J. (2019). Mugwort Pollen-Related Food Allergy: Lipid Transfer Protein Sensitization and Correlation With the Severity of Allergic Reactions in a Chinese Population. Allergy Asthma Immunol Res, 11(1), 116-128. doi:10.4168/aair.2019.11.1.116
54. Deng, X., Thurston, G., Zhang, W., Ryan, I., Jiang, C., Khwaja, H., . . . Lin, S. (2021). Application of data science methods to identify school and home risk factors for asthma and allergy-related symptoms among children in New York. Sci Total Environ, 770, 144746. doi:10.1016/j.scitotenv.2020.144746
55. Deng, Z. Y., Liu, X. J., Sa, R. N., Xu, H. X., Fu, Q., Xu, D. Y., . . . Liu, X. L. (2021). [Epidemiological investigation of allergic rhinitis in central cities and countrysides of Inner Mongolia region]. Zhonghua Er Bi Yan Hou Tou Jing Wai Ke Za Zhi, 56(6), 635-642. doi:10.3760/cma.j.cn115330-20200929-00778
56. Dong, G. H., Ren, W. H., Wang, D., Yang, Z. H., Zhang, P. F., Zhao, Y. D., & He, Q. C. (2011). Exposure to secondhand tobacco smoke enhances respiratory symptoms and responses to animals in 8,819 children in kindergarten: results from 25 districts in northeast China. Respiration, 81(3), 179-185. doi:10.1159/000321222
57. Du, C., Li, B., Yu, W., Yao, R., Cai, J., Li, B., . . . Essah, E. (2022). Characteristics of annual mold variations and association with childhood allergic symptoms/diseases via combining surveys and home visit measurements. Indoor Air, 32(9), e13113. doi:10.1111/ina.13113
58. Du, M., Xu, S., Qiu, B., Hu, S., Tjakkes, G. E., Li, A., & Ge, S. (2023). Serum antibodies to periodontal pathogens are related to allergic symptoms. J Periodontol, 94(2), 204-216. doi:10.1002/jper.22-0346
59. Duan, S., Zhao, L., Zhang, Y., Zhang, N., Zheng, M., Wang, Q., . . . Lan, F. (2021). Tropomyosin in mugwort cross-reacts to house dust mite, eliciting non-Th2 response in allergic rhinitis patients sensitized to house dust mite. Clin Mol Allergy, 19(1), 2. doi:10.1186/s12948-021-00142-z
60. Fan, E. Z., Xi, L., Han, D. M., Zhang, S. Z., Li, Y., & Zhang, L. (2009). [Evaluation of the safety of aluminium adjuvant in the preparation of allergic rhinitis animal model]. Zhonghua Er Bi Yan Hou Tou Jing Wai Ke Za Zhi, 44(8), 664-668.
61. Fan, G. K., Wang, H., & Takenaka, H. (2007). Eosinophil infiltration and activation in nasal polyposis. Acta Otolaryngol, 127(5), 521-526. doi:10.1080/00016480600951368
62. Fan, Z. Z., Lu, Z. Y., & Jin, J. Q. (2023). [Research progress of oral allergy syndrome]. Zhonghua Yu Fang Yi Xue Za Zhi, 57(3), 341-347. doi:10.3760/cma.j.cn112150-20220508-00458
63. Fang, H., Li, J., Ren, L., & Liu, E. (2023). Age-related differences in IgE between childhood and adulthood allergic asthma: Analysis of NHANES 2005-2006. World Allergy Organ J, 16(12), 100842. doi:10.1016/j.waojou.2023.100842
64. Fang, S. Y., Perng, D. W., Lee, J. Y., Lin, D. Y., & Huangs, C. Y. (2010). An open-label, multicentre study of levocetirizine for the treatment of allergic rhinitis and urticaria in Taiwanese patients. Chin J Physiol, 53(4), 199-207. doi:10.4077/cjp.2010.amk007
65. Feng, B., Wu, J., Chen, B., Xiang, H., Chen, R., Li, B., & Chen, S. (2017). Efficacy and safety of sublingual immunotherapy for allergic rhinitis in pediatric patients: A meta-analysis of randomized controlled trials. Am J Rhinol Allergy, 31(1), 27-35. doi:10.2500/ajra.2017.31.4382
66. Feng, J., Wu, Z., Wang, X., Zhang, Y., & Teng, N. (2019). Analysis of Pollen Allergens in Lily by Transcriptome and Proteome Data. Int J Mol Sci, 20(23). doi:10.3390/ijms20235892
67. Feng, S., Deng, C., Li, L., Liao, W., Fan, Y., Xu, G., & Li, H. (2014). [Efficacy of intranasal antihistamine in the treatment of allergic rhinitis: a meta-analysis]. Zhonghua Er Bi Yan Hou Tou Jing Wai Ke Za Zhi, 49(10), 832-838.
68. Feng, S., Fan, Y., Liang, Z., Ma, R., & Cao, W. (2016). Concomitant corticosteroid nasal spray plus antihistamine (oral or local spray) for the symptomatic management of allergic rhinitis. Eur Arch Otorhinolaryngol, 273(11), 3477-3486. doi:10.1007/s00405-015-3832-1
69. Feng, Y., Cao, Y., Liu, Y., Wang, Q., He, C., Hao, J., . . . HuangFu, H. (2023). Clinical efficacy and safety of coseasonal initiation of Artemisia annua sublingual immunotherapy on patients with Artemisia-induced rhinoconjunctivitis. Am J Otolaryngol, 44(5), 103942. doi:10.1016/j.amjoto.2023.103942
70. Feng, Y., Meng, Y. P., Dong, Y. Y., Qiu, C. Y., & Cheng, L. (2021). Management of allergic rhinitis with leukotriene receptor antagonists versus selective H1-antihistamines: a meta-analysis of current evidence. Allergy Asthma Clin Immunol, 17(1), 62. doi:10.1186/s13223-021-00564-z
71. Feng, Y., Wang, X., Wang, F., Liu, R., Chen, L., Wu, S., . . . Li, J. (2017). The Prevalence of Ocular Allergy and Comorbidities in Chinese School Children in Shanghai. Biomed Res Int, 2017, 7190987. doi:10.1155/2017/7190987
72. Fu, W., Gao, Z., Gao, L., Jin, J., Liu, M., Sun, Y., . . . van Ree, R. (2018). Identification of a 62-kDa major allergen from Artemisia pollen as a putative galactose oxidase. Allergy, 73(5), 1041-1052. doi:10.1111/all.13375
73. Fucheng, Y., Zheng, Z., Yichun, L., Yang, L., Luying, Z., Jiaxian, P., . . . Senlin, L. (2019). Subpollens delivery of Platanus acerifolia pollen allergen Pla a3 and nucleic acid into lungs and cells. Biochem Biophys Res Commun, 513(4), 767-774. doi:10.1016/j.bbrc.2019.03.210
74. Gao, H., Niu, Y., Wang, Q., Shan, G., Ma, C., Wang, H., . . . Liu, Y. (2021). Analysis of Prevalence and Risk Factors of Adult Self-Reported Allergic Rhinitis and Asthma in Plain Lands and Hilly Areas of Shenmu City, China. Front Public Health, 9, 749388. doi:10.3389/fpubh.2021.749388
75. Gao, J., Cai, R., & Zhou, N. (2021). Development of a sensitive and stable chemiluminescent immunoassay for detection of birch pollen allergic specific IgE based on recombinant Bet v1 protein. J Immunol Methods, 493, 113040. doi:10.1016/j.jim.2021.113040
76. Gao, Q., Zhang, Y., Wang, C., Zhao, Y., & Zhang, L. (2011). [Effect of topical steroid on oral and nasal nitric oxide production in patients with allergic rhinitis]. Lin Chuang Er Bi Yan Hou Tou Jing Wai Ke Za Zhi, 25(20), 913-915, 919.
77. Gao, S. S., Song, Z. S., Jia, Q., Li, X., Li, C. L., Chen, X. W., & Zheng, C. B. (2017). [Serum levels of periostin and their influencing factors in the beta-lactam antibiotic enterprise workers]. Zhonghua Lao Dong Wei Sheng Zhi Ye Bing Za Zhi, 35(4), 261-264. doi:10.3760/cma.j.issn.1001-9391.2017.04.005
78. Gao, Z., Fu, W. Y., Sun, Y., Gao, B., Wang, H. Y., Liu, M., . . . van Ree, R. (2019). Artemisia pollen allergy in China: Component-resolved diagnosis reveals allergic asthma patients have significant multiple allergen sensitization. Allergy, 74(2), 284-293. doi:10.1111/all.13597
79. Gao, Z., Huang, M., Qu, Z., Wang, J., & Cai, X. (2019). Identification of DNA methylation module in seasonal allergic rhinitis. Int J Pediatr Otorhinolaryngol, 117, 163-166. doi:10.1016/j.ijporl.2018.11.030
80. Gao, Z., van de Weg, E. W., Matos, C. I., Arens, P., Bolhaar, S. T., Knulst, A. C., . . . Gilissen, L. J. (2008). Assessment of allelic diversity in intron-containing Mal d 1 genes and their association to apple allergenicity. BMC Plant Biol, 8, 116. doi:10.1186/1471-2229-8-116
81. Gao, Z. S., Fu, W. Y., Zhao, L., Gao, L., Zhou, J. Y., Gao, B. Y., . . . van Ree, R. (2019). Localization of Four Allergens in Artemisia Pollen by Immunofluorescent Antibodies. Int Arch Allergy Immunol, 179(3), 165-172. doi:10.1159/000497321
82. Gao, Z. S., Yang, Z. W., Wu, S. D., Wang, H. Y., Liu, M. L., Mao, W. L., . . . van Ree, R. (2013). Peach allergy in China: a dominant role for mugwort pollen lipid transfer protein as a primary sensitizer. J Allergy Clin Immunol, 131(1), 224-226.e221-223. doi:10.1016/j.jaci.2012.07.015
83. Gao, Z. S., Zhou, X., Yang, Z. W., Versteeg, S. A., Gao, L., Fu, W. Y., . . . van Ree, R. (2016). IgE-binding potencies of three peach Pru p 1 isoforms. Mol Nutr Food Res, 60(11), 2457-2466. doi:10.1002/mnfr.201500798
84. Gu, H., Chen, X. S., Chen, K., Yan, Y., Jing, H., Chen, X. Q., . . . Ye, G. Y. (2001). Evaluation of diagnostic criteria for atopic dermatitis: validity of the criteria of Williams et al. in a hospital-based setting. Br J Dermatol, 145(3), 428-433. doi:10.1046/j.1365-2133.2001.04379.x
85. Gu, J., Qin, G., Jiang, L., Xu, W., Wang, Y., Liao, J., . . . Liang, Z. (2023). Correlations between IL-36 family cytokines in peripheral blood and subjective and objective assessment results in patients with allergic rhinitis. Allergy Asthma Clin Immunol, 19(1), 79. doi:10.1186/s13223-023-00834-y
86. Gu, J. L., Ma, H. L., & Zheng, Y. J. (2012). [Epidemiological survey of asthma in children aged 0-14 years in the Futian District of Shenzhen, China between 2010 and 2011]. Zhongguo Dang Dai Er Ke Za Zhi, 14(12), 918-923.
87. Gu, R. J. (1989). Nasal allergy in China. Proc Chin Acad Med Sci Peking Union Med Coll, 4(3), 167-171.
88. Guan, K., Zhou, J. X., Wang, R. Q., Yin, J., Wang, L. L., Zhi, Y. X., . . . Xu, T. (2018). [The clinical value of China Savin pollen extract used for skin prick test]. Lin Chuang Er Bi Yan Hou Tou Jing Wai Ke Za Zhi, 32(3), 161-166. doi:10.13201/j.issn.1001-1781.2018.03.001
89. Guan, K., Zhu, W., Sha, L., Liu, C., Zhao, J., Yin, J., & Chen, Y. (2022). Prevalence of Sensitization to Aeroallergens in Greater Beijing Region Children With Respiratory Allergy. Front Pediatr, 10, 848357. doi:10.3389/fped.2022.848357
90. Guo, J., Yu, D., Lv, N., Bai, R., Xu, C., Chen, G., & Cao, W. (2017). Relationships between acrylamide and glycidamide hemoglobin adduct levels and allergy-related outcomes in general US population, NHANES 2005-2006. Environ Pollut, 225, 506-513. doi:10.1016/j.envpol.2017.03.016
91. Han, D., Chen, L., Cheng, L., Liu, S., Fu, Z., Zhang, W., . . . Zhang, L. (2011). A multicenter randomized double-blind 2-week comparison study of azelastine nasal spray 0.1% versus levocabastine nasal spray 0.05% in patients with moderate-to-severe allergic rhinitis. ORL J Otorhinolaryngol Relat Spec, 73(5), 260-265. doi:10.1159/000330269
92. Han, D., Lai, X., Gjesing, B., Zhong, N., Zhang, L., & Spangfort, M. D. (2011). The specific IgE reactivity pattern of weed pollen-induced allergic rhinitis patients. Acta Otolaryngol, 131(5), 533-538. doi:10.3109/00016489.2010.539265
93. Han, D., Liu, S., Zhang, Y., Wang, J., Wang, D., Kong, W., . . . Zhang, L. (2011). Efficacy and safety of fluticasone furoate nasal spray in Chinese adult and adolescent subjects with intermittent or persistent allergic rhinitis. Allergy Asthma Proc, 32(6), 472-481. doi:10.2500/aap.2011.32.3474
94. Han, D., She, W., & Zhang, L. (2010). Association of the CD14 gene polymorphism C-159T with allergic rhinitis. Am J Rhinol Allergy, 24(1), e1-3. doi:10.2500/ajra.2010.24.3411
95. Han, D., Xi, L., Fan, E., Li, Y., & Zhang, L. (2009). Expression of the Foxp3 gene in spleen mononuclear cells of a mouse model with allergic rhinitis. ORL J Otorhinolaryngol Relat Spec, 71(6), 317-322. doi:10.1159/000267304
96. Han, D. M. (2006). [Allergic rhinitis in China]. Zhonghua Er Bi Yan Hou Tou Jing Wai Ke Za Zhi, 41(7), 481-482.
97. Han, D. M., Zhang, L., Huang, D., Wu, Y. F., Dong, Z., Xu, G., . . . Wang, Q. P. (2007). [Self-reported prevalence of allergic rhinitis in eleven cities in China]. Zhonghua Er Bi Yan Hou Tou Jing Wai Ke Za Zhi, 42(5), 378-384.
98. Han, D. M., Zhou, B., Wang, T., Wang, X. D., & Fan, E. Z. (2006). Intranasal application of Epstein-Barr virus/lipoplex to abrogate eosinophillia in murine model of allergic rhinitis. Chin Med J (Engl), 119(12), 991-997.
99. Han, J., Wang, W., Zhu, Z., Wang, L., Chen, Y., Wang, R., . . . Lv, W. (2022). Profile of Tissue Immunoglobulin E in Eosinophilic Chronic Rhinosinusitis with Nasal Polyps. Int Arch Allergy Immunol, 183(8), 835-842. doi:10.1159/000522624
100. Han, J. L., Ren, Z., & Jiang, X. J. (2008). [Expression and significance of galectin-3 and galectin-9 in mice nasal mucosa with allergic rhinitis]. Zhonghua Er Bi Yan Hou Tou Jing Wai Ke Za Zhi, 43(7), 504-508.
101. Han, P., Chen, L., Chen, D., Yang, R., Wang, W., Liu, J., . . . Zhang, H. (2022). Upregulated expression of substance P and NK1R in blood monocytes and B cells of patients with allergic rhinitis and asthma. Clin Exp Immunol, 210(1), 39-52. doi:10.1093/cei/uxac074
102. Han, S. H., Chang, Z. N., Chang, H. H., Chi, C. W., Wang, J. Y., & Lin, C. Y. (1993). Identification and characterization of epitopes on Cyn d I, the major allergen of Bermuda grass pollen. J Allergy Clin Immunol, 91(5), 1035-1041. doi:10.1016/0091-6749(93)90217-4
103. Han, S. H., Chang, Z. N., Chi, C. W., Perng, H. J., Liu, C. C., Tsai, J. J., & Tam, M. F. (1993). Use of monoclonal antibodies to isolate and characterize Cyn d I, the major allergen of Bermuda grass pollen. J Allergy Clin Immunol, 92(4), 549-558. doi:10.1016/0091-6749(93)90079-u
104. Han, Y., & Zhang, H. (2009). [Epidemiological investigation of allergic rhinitis in the primary school students in grade three of Shihezi city]. Lin Chuang Er Bi Yan Hou Tou Jing Wai Ke Za Zhi, 23(23), 1074-1078.
105. Hao, G. D., Zheng, Y. W., Gjesing, B., Kong, X. A., Wang, J. Y., Song, Z. J., . . . Spangfort, M. D. (2013). Prevalence of sensitization to weed pollens of Humulus scandens, Artemisia vulgaris, and Ambrosia artemisiifolia in northern China. J Zhejiang Univ Sci B, 14(3), 240-246. doi:10.1631/jzus.B1200185
106. Hao, G. D., Zheng, Y. W., Wang, Z. X., Kong, X. A., Song, Z. J., Lai, X. X., & Spangfort, M. D. (2016). High correlation of specific IgE sensitization between birch pollen, soy and apple allergens indicates pollen-food allergy syndrome among birch pollen allergic patients in northern China. J Zhejiang Univ Sci B, 17(5), 399-404. doi:10.1631/jzus.B1500279
107. Hon, K. L., Leung, T. F., Kam, W. Y., Lam, M. C., Wong, K. Y., Yung, E., & Fok, T. F. (2006). Exhaled nitric oxide levels are not correlated with eczema severity in Chinese children with atopic dermatitis. J Asthma, 43(6), 417-419. doi:10.1080/02770900600701341
108. Hong, H., Fan, Y., Yang, Q., Li, X., Guo, J., & Chen, X. (2011). [A cohort investigation of the allergen and pathogenesis in the patients with allergic rhinitis from three hospital of Guangdong Province]. Lin Chuang Er Bi Yan Hou Tou Jing Wai Ke Za Zhi, 25(23), 1064-1067.
109. Hong, Q., Zhou, S., Zhao, H., Peng, J., Li, Y., Shang, Y., . . . Wang, Q. (2018). Allergenicity of recombinant Humulus japonicus pollen allergen 1 after combined exposure to ozone and nitrogen dioxide. Environ Pollut, 234, 707-715. doi:10.1016/j.envpol.2017.11.078
110. Hong, S., Huang, J., & Bai, Y. (2001). [A survey of pollens in Chongqing]. Lin Chuang Er Bi Yan Hou Ke Za Zhi, 15(12), 558-560.
111. Hou, J., Lou, H., Wang, Y., He, F., Cao, F., Wang, C., & Zhang, L. (2018). Nasal ventilation is an important factor in evaluating the diagnostic value of nasal nitric oxide in allergic rhinitis. Int Forum Allergy Rhinol, 8(6), 686-694. doi:10.1002/alr.22087
112. Hou, X., Luo, W., Gan, H., Chen, T., & Sun, B. (2022). Childhood blood eosinophils and symptoms of allergic disorders: a cross-sectional study in Southern China. Ann Med, 54(1), 2929-2940. doi:10.1080/07853890.2022.2134584
113. Hou, X., Luo, W., Wu, L., Chen, Y., Li, G., Zhang, R., . . . Sun, B. (2022). Associations of Four sensitization patterns revealed by Latent Class Analysis with Clinical symptoms: A multi-center study of China. EClinicalMedicine, 46, 101349. doi:10.1016/j.eclinm.2022.101349
114. Hu, H., Huang, Z., Luo, W., Zou, X., Chen, H., Liao, C., & Sun, B. (2021). Allergen diagnosis strategy: An experimental application of different methods in Guangzhou, Southern China. Sci Prog, 104(1), 36850421997277. doi:10.1177/0036850421997277
115. Hu, H., Luo, W., Wu, Z., Cai, C., Huang, H., & Sun, B. (2019). A pilot study on the allergen-specific IgE to molecular components on polysensitized mite allergic asthmatic patients in Guangzhou, China. Mol Immunol, 105, 38-45. doi:10.1016/j.molimm.2018.11.004
116. Hu, S. J., Wei, P., Kou, W., Wu, X. F., Liu, M. Y., Chen, C., & Yao, H. B. (2017). [Prevalence and risk factors of allergic rhinitis: a Meta-analysis]. Lin Chuang Er Bi Yan Hou Tou Jing Wai Ke Za Zhi, 31(19), 1485-1491. doi:10.13201/j.issn.1001-1781.2017.19.006
117. Hu, W., Zhang, T., Zhang, Y., Meng, C., Xie, L., Song, Y., . . . Zhu, L. (2023). [A multi-center observation of the therapeutic efficacy of Bencycloquidium bromide in the treatment of seasonal allergic rhinitis with predominant symptoms of rhinorrhea]. Lin Chuang Er Bi Yan Hou Tou Jing Wai Ke Za Zhi, 37(7), 550-555. doi:10.13201/j.issn.2096-7993.2023.07.008
118. Hu, W. N., Zhu, L., Xie, L. F., Zhang, F. Z., Bai, M. Y., Wang, N., & Sun, Z. W. (2017). [Correlation between the visiting rate of patients with allergic rhinitis and airborne pollen concentrations in Beijing in recent 3 years]. Zhonghua Er Bi Yan Hou Tou Jing Wai Ke Za Zhi, 52(1), 31-36. doi:10.3760/cma.j.issn.1673-0860.2017.01.006
119. Huang, C., Liu, W., Cai, J., Weschler, L. B., Wang, X., Hu, Y., . . . Sundell, J. (2017). Breastfeeding and timing of first dietary introduction in relation to childhood asthma, allergies, and airway diseases: A cross-sectional study. J Asthma, 54(5), 488-497. doi:10.1080/02770903.2016.1231203
120. Huang, C., Liu, W., Hu, Y., Zou, Z., Zhao, Z., Shen, L., . . . Sundell, J. (2015). Updated prevalences of asthma, allergy, and airway symptoms, and a systematic review of trends over time for childhood asthma in Shanghai, China. PLoS ONE, 10(4), e0121577. doi:10.1371/journal.pone.0121577
121. Huang, F., Zhao, Y., He, J., Deng, P., Zhou, P., Zhou, J., . . . Yang, Z. (2010). [Analyzing of the inhaled allergens profiles of allergic rhinitis patients in district of Jingmen]. Lin Chuang Er Bi Yan Hou Tou Jing Wai Ke Za Zhi, 24(8), 341-343.
122. Huang, G. J., Lin, B. R., Fan, Z. J., & Lu, B. Q. (2022). Ragweed sublingual immunotherapy tablets in allergic rhinoconjunctivitis. Eur Arch Otorhinolaryngol, 279(12), 5965-5966. doi:10.1007/s00405-022-07375-x
123. Huang, J., Huang, D. M., Xiao, X. X., Fu, S. M., Luo, C. M., Zeng, G., . . . Guo, X. L. (2015). [Epidemiological survey of asthma among children aged 0-14 years in 2010 in urban Zhongshan, China]. Zhongguo Dang Dai Er Ke Za Zhi, 17(2), 149-154.
124. Huang, T. H., Wu, D. W., & Wei, Y. X. (2021). [Research progress of allergic rhinitis-related psychological disorders]. Zhonghua Er Bi Yan Hou Tou Jing Wai Ke Za Zhi, 56(8), 876-880. doi:10.3760/cma.j.cn115330-20200828-00707
125. Huang, Y., Wang, C., Zhang, Y., & Zhang, L. (2021). Developing nomograms for identifying allergic rhinitis among chronic rhinitis: A real-world study. World Allergy Organ J, 14(4), 100534. doi:10.1016/j.waojou.2021.100534
126. Huang, Y., Zhang, Y., Wang, J., Li, X., Wang, C., Chen, R., & Zhang, L. (2022). Impact of Air Pollutants and Pollen on the Severity of Nonallergic Rhinitis: A Data-Oriented Analysis. J Asthma Allergy, 15, 1045-1054. doi:10.2147/jaa.S372927
127. Jiang, M., Wang, Q., Li, Z., Xue, F., & Ji, J. (2006). [The epidemic survey of children rhinosinal inflammatory diseases and their correlation with asthma in Nanjing]. Lin Chuang Er Bi Yan Hou Ke Za Zhi, 20(20), 928-930.
128. Jiang, N., Xiang, L., Guan, H., & Zhang, X. (2023). Blueberry (Vaccinium myrtillus) Induced Anaphylaxis in a Chinese Child with Lipid Transfer Protein Sensitization. J Asthma Allergy, 16, 1253-1258. doi:10.2147/jaa.S436561
129. Jiang, N., Xu, W., Huang, H., Hou, X., & Xiang, L. (2022). Anaphylaxis in Chinese Children with Pollen Sensitization: Triggers, Clinical Presentation, and Acute Management. J Asthma Allergy, 15, 633-643. doi:10.2147/jaa.S363113
130. Jiang, N., Yin, J., Mak, P., & Wen, L. (2015). Occupational Allergy to Peach (Prunus persica) Tree Pollen and Potential Cross-Reactivity between Rosaceae Family Pollens. Iran J Allergy Asthma Immunol, 14(5), 483-492.
131. Jiang, N., Yin, J., Wen, L., & Li, H. (2016). Characteristics of Anaphylaxis in 907 Chinese Patients Referred to a Tertiary Allergy Center: A Retrospective Study of 1,952 Episodes. Allergy Asthma Immunol Res, 8(4), 353-361. doi:10.4168/aair.2016.8.4.353
132. Jiang, X. D., Li, G. Y., Dong, Z., & Zhu, D. D. (2011). Correlation analysis of two serum-specific immunoglobulin E test systems and skin-prick test in allergic rhinitis patients from northeast China. Am J Rhinol Allergy, 25(2), 116-119. doi:10.2500/ajra.2011.25.3572
133. Jiang, Z., Xiao, H., Zhang, H., Jia, Q., Lin, H., Zhang, L., . . . Wang, L. (2022). Broussonetia papyrifera (paper mulberry) pollen is an important cause of allergic rhinitis in Southwest China. Clin Exp Allergy, 52(12), 1448-1451. doi:10.1111/cea.14236
134. Jiao, Y. X., Song, L. B., Xu, Z. Q., Zhu, D. X., Yang, Y. S., Tian, M., . . . Wei, J. F. (2022). Purification and characterization of enolase as a novel allergen in Platanus acerifolia pollen. Int Immunopharmacol, 113(Pt A), 109313. doi:10.1016/j.intimp.2022.109313
135. Jin, J. J., Zou, Y. X., & Zeng, S. W. (2016). Risk factors for and expression of immune and inflammatory factors in atopic dermatitis in Chinese population: A birth cohort study. Mol Cell Probes, 30(3), 168-173. doi:10.1016/j.mcp.2016.03.006
136. Jin, P., Zhang, H., Zhu, X., Sun, K., Jiang, T., Shi, L., . . . Zhang, H. (2022). Bioinformatics analysis of mRNA profiles and identification of microRNA-mRNA network in CD4(+) T cells in seasonal allergic rhinitis. J Int Med Res, 50(8), 3000605221113918. doi:10.1177/03000605221113918
137. Kang, I. G., Ju, Y. H., Jung, J. H., Ko, K. P., Oh, D. K., Kim, J. H., . . . Kim, S. T. (2015). The effect of PM10 on allergy symptoms in allergic rhinitis patients during spring season. Int J Environ Res Public Health, 12(1), 735-745. doi:10.3390/ijerph120100735
138. Kong, R., & Yin, J. (2019). Efficacy of subcutaneous immunotherapy in a mouse model of Humulus pollen-induced allergic asthma. Am J Transl Res, 11(1), 280-289.
139. Kong, R., & Yin, J. (2021). Kidney-bean (Phaseolus Vulgaris) Dependent, Exercise-induced Anaphylaxis in Patients Comorbid with Mugwort (Artemisia Vulgaris) Pollinosis. Immunol Invest, 50(4), 389-398. doi:10.1080/08820139.2020.1770783
140. Kong, W., Chen, J., Wang, Y., Xiang, J., Zhang, X., Wang, J., & Yin, X. (2012). A population-based 5-year follow-up of allergic rhinitis in Chinese children. Am J Rhinol Allergy, 26(4), 315-320. doi:10.2500/ajra.2012.26.3790
141. Kong, W., Xie, Y., Zhong, J., & Cao, C. (2022). Ultra-processed foods and allergic symptoms among children and adults in the United States: A population-based analysis of NHANES 2005-2006. Front Public Health, 10, 1038141. doi:10.3389/fpubh.2022.1038141
142. Kong, W. J., Chen, J. J., Zheng, Z. Y., Shi, Q. M., & Zhou, Y. (2009). Prevalence of allergic rhinitis in 3-6-year-old children in Wuhan of China. Clin Exp Allergy, 39(6), 869-874. doi:10.1111/j.1365-2222.2009.03206.x
143. Kuang, L., Feng, H. L., & Xie, B. B. (2012). [Efficacy and compliance of specific immunotherapy in allergic rhinitis]. Lin Chuang Er Bi Yan Hou Tou Jing Wai Ke Za Zhi, 26(2), 56-58. doi:10.13201/j.issn.1001-1781.2012.02.009
144. Lam, S. C., Yeung, C. C. Y., Chan, J. H. M., Lam, D. W. C., Lam, A. H. Y., Annesi-Maesano, I., & Bousquet, J. (2017). Adaptation of the Score for Allergic Rhinitis in the Chinese Population: Psychometric Properties and Diagnostic Accuracy. Int Arch Allergy Immunol, 173(4), 213-224. doi:10.1159/000477727
145. Lei, F., Cui, Y. N., Jiang, Y. P., Kong, H., Zhu, D. D., & Dong, Z. (2008). [Effects of minimal persistent inflammation on nasal mucosa of experimental allergic rhinitis in guinea pigs]. Zhonghua Er Bi Yan Hou Tou Jing Wai Ke Za Zhi, 43(7), 499-503.
146. Lei, F., Zhu, D., Sun, J., & Dong, Z. (2010). Effects of minimal persistent inflammation on nasal mucosa of experimental allergic rhinitis. Am J Rhinol Allergy, 24(1), e23-28. doi:10.2500/ajra.2010.24.3414
147. Lei, F., Zhu, D. D., Kong, H., Cui, Y. N., Jiang, Y. P., & Dong, Z. (2008). [Establishment and significance of the experimental minimal persistent inflammation models in allergic rhinitis guinea pigs]. Zhonghua Er Bi Yan Hou Tou Jing Wai Ke Za Zhi, 43(6), 443-446.
148. Leng, X., & Ye, S. T. (1987). An investigation on in vivo allergenicity of Artemisia annua leaves and stems. Asian Pac J Allergy Immunol, 5(2), 125-128.
149. Leng, X., & Ye, S. T. (1987). One year observation of immunotherapy for Artemisia hay fever in China: a clinical and immunological study. Asian Pac J Allergy Immunol, 5(2), 167-172.
150. Leung, R., & Jenkins, M. (1994). Asthma, allergy and atopy in southern Chinese school students. Clin Exp Allergy, 24(4), 353-358. doi:10.1111/j.1365-2222.1994.tb00246.x
151. Li, C., Cheung, C. L., Cheung, T. T., Samaranayake, N. R., & Cheung, B. M. (2014). Hay fever and hypertension in the US adult population. Clin Exp Hypertens, 36(4), 206-210. doi:10.3109/10641963.2013.804545
152. Li, C. S., Chae, S. C., Lee, J. H., Zhang, Q., & Chung, H. T. (2006). Identification of single nucleotide polymorphisms in FOXJ1 and their association with allergic rhinitis. J Hum Genet, 51(4), 292-297. doi:10.1007/s10038-006-0359-8
153. Li, C. W., Chen, D. D., Zhong, J. T., Lin, Z. B., Peng, H., Lu, H. G., . . . Li, T. Y. (2014). Epidemiological characterization and risk factors of allergic rhinitis in the general population in Guangzhou City in china. PLoS ONE, 9(12), e114950. doi:10.1371/journal.pone.0114950
154. Li, C. Y., Huang, H. Y., Liu, X. J., & Liu, X. L. (2022). [Research progress on the characteristics of artemisia pollen allergens and related pollinosis]. Zhonghua Yu Fang Yi Xue Za Zhi, 56(6), 748-754. doi:10.3760/cma.j.cn112150-20220314-00232
155. Li, D., Ou, Q., Shen, Q., Lu, M. M., Xu, J. Y., Jin, C., . . . Tian, H. (2023). Subconjunctival injection of human umbilical cord mesenchymal stem cells alleviates experimental allergic conjunctivitis via regulating T cell response. Stem Cell Res Ther, 14(1), 281. doi:10.1186/s13287-023-03484-4
156. Li, D. D., & He, S. H. (2003). Plant allergic proteins and their biological functions. Sheng Wu Hua Xue Yu Sheng Wu Wu Li Xue Bao (Shanghai), 35(9), 789-792.
157. Li, H., Zhang, H., Yin, J., Wang, R., & Fang, Q. (2004). [Clinical examination of mizolastine in the seasonal allergic rhinitis]. Lin Chuang Er Bi Yan Hou Ke Za Zhi, 18(8), 479-481.
158. Li, J., Fang, F., Mei, M., & Wu, D. (2023). The gut microbiome and allergic rhinitis; refocusing on the role of probiotics as a treatment option. Eur Arch Otorhinolaryngol, 280(2), 511-517. doi:10.1007/s00405-022-07694-z
159. Li, J., Huang, Y., Lin, X., Zhao, D., Tan, G., Wu, J., . . . Zhong, N. (2011). Influence of degree of specific allergic sensitivity on severity of rhinitis and asthma in Chinese allergic patients. Respir Res, 12(1), 95. doi:10.1186/1465-9921-12-95
160. Li, J., Lu, Y., Huang, K., Wang, C., Lu, J., Zhang, C., & Zhong, N. (2008). Chinese response to allergy and asthma in Olympic athletes. Allergy, 63(8), 962-968. doi:10.1111/j.1398-9995.2008.01816.x
161. Li, J., Luo, D. F., Li, S. Y., Sun, B. Q., & Zhong, N. S. (2005). Efficacy of intramuscular BCG polysaccharide nucleotide on mild to moderate bronchial asthma accompanied with allergic rhinitis: a randomized, double blind, placebo-controlled study. Chin Med J (Engl), 118(19), 1595-1603.
162. Li, J., Sun, B., Huang, Y., Lin, X., Zhao, D., Tan, G., . . . Zhong, N. (2009). A multicentre study assessing the prevalence of sensitizations in patients with asthma and/or rhinitis in China. Allergy, 64(7), 1083-1092. doi:10.1111/j.1398-9995.2009.01967.x
163. Li, J., Wang, C., Liu, C., Kang, J., Kong, L., Huang, Y., . . . Zhong, N. (2020). Efficacy predictors of omalizumab in Chinese patients with moderate-to-severe allergic asthma: Findings from a post-hoc analysis of a randomised phase III study. World Allergy Organ J, 13(12), 100469. doi:10.1016/j.waojou.2020.100469
164. Li, J., Wang, F., Cui, Y., Song, L., Yu, Y., & Chen, X. (2022). Identification of Diagnostic Biomarkers of Rape Pollen Allergy Based on MRNA Sequencing. Evid Based Complement Alternat Med, 2022, 6153577. doi:10.1155/2022/6153577
165. Li, J., Wang, H. Y., Zhang, C. Q., Sun, B. Q., & Zhong, N. S. (2006). Links between allergic rhinitis and asthma. Chin Med J (Engl), 119(8), 676-683.
166. Li, J. D., Du, Z. R., Liu, J., Xu, Y. Y., Wang, R. Q., & Yin, J. (2020). Characteristics of pollen-related food allergy based on individual pollen allergy profiles in the Chinese population. World Allergy Organ J, 13(5), 100120. doi:10.1016/j.waojou.2020.100120
167. Li, J. D., Gu, J. Q., Xu, Y. Y., Cui, L., Li, L. S., Wang, Z. X., . . . Guan, K. (2022). Serum IgE profiles in Chinese pollinosis patients with grass pollen sensitisation. World Allergy Organ J, 15(1), 100624. doi:10.1016/j.waojou.2021.100624
168. Li, L., Chang, C., & Guan, K. (2022). Birch Pollen Allergens. Curr Protein Pept Sci, 23(11), 731-743. doi:10.2174/1389203723666220815095725
169. Li, L., & Guan, K. (2016). Effect on quality of life of the mixed house dust mite/weed pollen extract immunotherapy. Asia Pac Allergy, 6(3), 168-173. doi:10.5415/apallergy.2016.6.3.168
170. Li, L., Hao, D., Li, X., Chen, M., Zhou, Y., Jurgens, D., . . . Sapkota, A. (2022). Satellite-based phenology products and in-situ pollen dynamics: A comparative assessment. Environ Res, 204(Pt A), 111937. doi:10.1016/j.envres.2021.111937
171. Li, L., Hart, J. E., Coull, B. A., Cao, S. J., Spengler, J. D., & Adamkiewicz, G. (2019). Effect of Residential Greenness and Nearby Parks on Respiratory and Allergic Diseases among Middle School Adolescents in a Chinese City. Int J Environ Res Public Health, 16(6). doi:10.3390/ijerph16060991
172. Li, L., Lang, Y. Y., Zhang, J. Y., & Shen, B. (2016). [Clinical analysis of food serum specific IgE in children with allergic rhinitis in Tianjin]. Lin Chuang Er Bi Yan Hou Tou Jing Wai Ke Za Zhi, 30(9), 718-721;725. doi:10.13201/j.issn.1001-1781.2016.09.012
173. Li, L., Liu, H., Wang, Y., Han, X., Ge, T., & Pan, L. (2020). Construction of a nomogram for predicting the risk of allergic rhinitis among employees of long-distance bus stations in China. Indoor Air, 30(6), 1178-1188. doi:10.1111/ina.12694
174. Li, L., Liu, Y., Wang, Q., Wang, Z., Cui, L., Xu, Y., & Guan, K. (2021). Levels of nasal exhaled hydrogen sulfide in the general population and allergic rhinitis patients. J Clin Lab Anal, 35(3), e23678. doi:10.1002/jcla.23678
175. Li, L., Wang, R., Cui, L., & Guan, K. (2018). Efficacy of montelukast as prophylactic treatment for seasonal allergic rhinitis. Ear Nose Throat J, 97(7), E12-e16.
176. Li, L., Wang, Z., Cui, L., Xu, Y., Lee, H., & Guan, K. (2023). The efficacy of a novel smart watch on medicine adherence and symptom control of allergic rhinitis patients: Pilot study. World Allergy Organ J, 16(1), 100739. doi:10.1016/j.waojou.2022.100739
177. Li, L., Wen, X., Gong, Y., Chen, Y., Xu, J., Sun, J., . . . Guan, K. (2023). HMGN2 and Histone H1.2: potential targets of a novel probiotic mixture for seasonal allergic rhinitis. Front Microbiol, 14, 1202858. doi:10.3389/fmicb.2023.1202858
178. Li, L., Zhang, H., Wang, X., Wang, Z., Cui, L., Xu, Y., & Guan, K. (2022). Upregulated antimicrobial immune response mediated by neutrophils in the development from allergic rhinitis to asthma. Front Immunol, 13, 1026121. doi:10.3389/fimmu.2022.1026121
179. Li, L., Zhang, L., Mo, J. H., Li, Y. Y., Xia, J. Y., Bai, X. B., . . . Chen, Q. Y. (2020). Efficacy of indoor air purification in the treatment of Artemisia pollen-allergic rhinitis: A randomised, double-blind, clinical controlled trial. Clin Otolaryngol, 45(3), 394-401. doi:10.1111/coa.13514
180. Li, L., Zhang, Y., Liu, X., Li, J., Yang, Q., Jiang, J., . . . Chen, W. (2023). Potential causal association between aspirin use and the reduced risk of hayfever or allergic rhinitis: a Mendelian randomization study. Front Immunol, 14, 1232981. doi:10.3389/fimmu.2023.1232981
181. Li, L. F., & Wang, J. (2000). Patch testing and aeroallergen intradermal testing in facial dermatitis. Contact Dermatitis, 43(2), 90-94. doi:10.1034/j.1600-0536.2000.043002090.x
182. Li, L. S., Guan, K., Cui, L., & Wang, R. Q. (2018). [Effects of double-dose antihistamine or combined therapy in allergic rhinitis]. Lin Chuang Er Bi Yan Hou Tou Jing Wai Ke Za Zhi, 32(1), 27-32. doi:10.13201/j.issn.1001-1781.2018.01.006
183. Li, L. S., Guan, K., Li, J. D., Wang, Z. X., Cui, L., & Xu, Y. Y. (2021). [The effect on patient life quality of prophylactic treatment to seasonal allergic rhinitis and related transcriptomics research]. Zhonghua Yu Fang Yi Xue Za Zhi, 55(5), 606-612. doi:10.3760/cma.j.cn112150-20210228-00198
184. Li, L. S., Guan, K., Wang, Z. X., Cui, L., & Xu, Y. Y. (2020). [The comparison of step-down pharmacotherapy and maintaining dose therapy for seasonal allergic rhinitis]. Lin Chuang Er Bi Yan Hou Tou Jing Wai Ke Za Zhi, 34(1), 9-12;18. doi:10.13201/j.issn.1001-1781.2020.01.003
185. Li, L. S., Guan, K., Wang, Z. X., Cui, L., & Xu, Y. Y. (2021). [Appropriate protocol of treatment modulation for seasonal allergic rhinitis caused by cypress pollens]. Zhonghua Yi Xue Za Zhi, 101(17), 1256-1261. doi:10.3760/cma.j.cn112137-20200916-02656
186. Li, L. S., Guan, K., Yin, J., Wang, L. L., Zhi, Y. X., Sun, J. L., . . . Bian, S. N. (2023). [Risk factors of systemic allergic reactions caused by subcutaneous allergen immunotherapy]. Zhonghua Yu Fang Yi Xue Za Zhi, 57(12), 1972-1977. doi:10.3760/cma.j.cn112150-20230703-00500
187. Li, L. S., Wang, R. Q., & Guan, K. (2017). [Treatment effect of sodium pyruvate nasal spray on allergic rhinitis]. Lin Chuang Er Bi Yan Hou Tou Jing Wai Ke Za Zhi, 31(2), 103-106. doi:10.13201/j.issn.1001-1781.2017.02.006
188. Li, P., Xiao, J., Liu, J., Liu, R., Bi, K., & Li, Q. (2018). Development and full validation of a liquid chromatography-tandem mass spectrometry method for determination of carbinoxamine in beagle plasma and its application to a pharmacokinetic study. J Chromatogr B Analyt Technol Biomed Life Sci, 1093-1094, 183-189. doi:10.1016/j.jchromb.2018.05.002
189. Li, P., Xu, H., & Qiu, M. (2010). [The correlative study between skin prick test with antigens and nasal provoking test in patients with allergic rhinitis]. Lin Chuang Er Bi Yan Hou Tou Jing Wai Ke Za Zhi, 24(2), 60-62.
190. Li, Q., Li, X. L., Yang, X., Bao, J. M., & Shen, X. H. (2009). Effects of antiallergic herbal agents on cystic fibrosis transmembrane conductance regulator in nasal mucosal epithelia of allergic rhinitis rabbits. Chin Med J (Engl), 122(24), 3020-3024.
191. Li, Q., Zhang, X., Feng, Q., Zhou, H., Ma, C., Lin, C., . . . Yin, J. (2023). Common Allergens and Immune Responses Associated with Allergic Rhinitis in China. J Asthma Allergy, 16, 851-861. doi:10.2147/jaa.S420328
192. Li, S., Yang, Q., Qiong, D., & Gao, C. (2010). [Epidemiological investigation on relativity of allergic rhinitis and recurring respiratory infection of preschool children in Shenzhen]. Lin Chuang Er Bi Yan Hou Tou Jing Wai Ke Za Zhi, 24(23), 1060-1061.
193. Li, W. J., Huang, N., Zhang, S. C., Ma, D. X., Yang, Y. Q., & Zhu, R. F. (2021). [Airborne pollens in Wuhan urban area and its correlation with hospital visits of allergic rhinitis patients from 2017 to 2018]. Zhonghua Yu Fang Yi Xue Za Zhi, 55(5), 620-626. doi:10.3760/cma.j.cn112150-20210312-00248
194. Li, W. K., & Wang, C. S. (1986). Survey of air-borne allergic pollens in North China: contamination with ragweed. N Engl Reg Allergy Proc, 7(2), 134-143.
195. Li, X., Lee, E. J., Lilja, S., Loscalzo, J., Schäfer, S., Smelik, M., . . . Benson, M. (2022). A dynamic single cell-based framework for digital twins to prioritize disease genes and drug targets. Genome Med, 14(1), 48. doi:10.1186/s13073-022-01048-4
196. Li, X., Zhang, Q., Jiang, L., Li, T., Liu, M., Liu, H., . . . Zhang, F. (2013). Clinical effect of catgut implantation at acupoints for allergic rhinitis: study protocol for a randomized controlled trial. Trials, 14, 12. doi:10.1186/1745-6215-14-12
197. Li, Y., Cheng, L., Chen, X., Yang, B., & Wang, D. (2015). Efficacy evaluation of a pollen blocker cream against dust-mite allergy: A multicenter, randomized, double-blind, placebo-controlled crossover trial. Am J Rhinol Allergy, 29(5), e129-133. doi:10.2500/ajra.2015.29.4218
198. Li, Y., & Liu, F. (2008). [The effect of desloratadine on quality of life of patients with seasonal allergic rhinitis]. Lin Chuang Er Bi Yan Hou Tou Jing Wai Ke Za Zhi, 22(10), 439-442.
199. Li, Y., Ouyang, Y., Jiao, J., Xu, Z., & Zhang, L. (2021). Exposure to environmental black carbon exacerbates nasal epithelial inflammation via the reactive oxygen species (ROS)-nucleotide-binding, oligomerization domain-like receptor family, pyrin domain containing 3 (NLRP3)-caspase-1-interleukin 1β (IL-1β) pathway. Int Forum Allergy Rhinol, 11(4), 773-783. doi:10.1002/alr.22669
200. Li, Y., Wang, D., Liu, Q., & Liu, J. (2013). Randomized double-blind placebo-controlled crossover study of efficacy of pollen blocker cream for perennial allergic rhinitis. Am J Rhinol Allergy, 27(4), 299-303. doi:10.2500/ajra.2013.27.3923
201. Li, Y. X., Chen, D., Li, T. Y., Feng, L. Q., Xu, G., & Wen, W. P. (2008). [Effect of garlicin on the serum levels of interleukin 4 and interferon gamma in allergic rhinitis model in rats]. Zhonghua Er Bi Yan Hou Tou Jing Wai Ke Za Zhi, 43(6), 447-450.
202. Liao, C., Hou, X., Wu, L., Luo, W., Zhang, H., Sun, X., . . . Sun, B. (2022). Major Grass Pollen Allergen Components and Cross-Reactive Carbohydrate Determinants in Mugwort-Sensitized Child Patients With Allergic Respiratory Disease in Western China. Front Pediatr, 10, 816354. doi:10.3389/fped.2022.816354
203. Liao, C., Liang, C., Hu, H., Luo, W., Wu, G., Huang, Z., . . . Sun, B. (2020). Major Pollen Allergen Components and CCD Detection in Bermuda Grass Sensitized Patients in Guangzhou, China. J Asthma Allergy, 13, 615-623. doi:10.2147/jaa.S277704
204. Liao, C. X., Huang, D. M., Hu, H. S., Luo, W. T., Wu, L. T., Zhu, H. Q., & Sun, B. Q. (2021). [Analysis of serum allergen-specific IgE in children with respiratory allergic disease in Guangdong]. Zhonghua Yu Fang Yi Xue Za Zhi, 55(5), 613-619. doi:10.3760/cma.j.cn112150-20201217-01467
205. Lin, M. H., Hsieh, C. J., Caffrey, J. L., Lin, Y. S., Wang, I. J., Ho, W. C., . . . Lin, R. S. (2015). Fetal Growth, Obesity, and Atopic Disorders in Adolescence: a Retrospective Birth Cohort Study. Paediatr Perinat Epidemiol, 29(5), 472-479. doi:10.1111/ppe.12215
206. Lin, X., He, S., & Xie, H. (2005). [Correlations between plasma levels of IL-18, IL-4, IFN-gamma, IgE and ECP in the patients with seasonal allergic rhinitis and their clinical implications]. Lin Chuang Er Bi Yan Hou Ke Za Zhi, 19(5), 195-197.
207. Ling, X. J., Zhou, Y. J., Yang, Y. S., Xu, Z. Q., Wang, Y., Sun, J. L., . . . Wei, J. F. (2022). A new cysteine protease allergen from Ambrosia trifida pollen: proforms and mature forms. Mol Immunol, 147, 170-179. doi:10.1016/j.molimm.2022.05.003
208. Liu, C., Han, D., Zhang, L., & Zhao, Y. (2009). [Comparison of two specific immunoglobulin E test systems in the diagnosis of allergic rhinitis]. Lin Chuang Er Bi Yan Hou Tou Jing Wai Ke Za Zhi, 23(11), 484-487.
209. Liu, C. Y., Zhang, Y., Han, D. M., & Zhang, L. (2010). Evaluation of serum specific IgE for the diagnosis of allergic rhinitis with multi-allergens. Chin Med J (Engl), 123(20), 2836-2841.
210. Liu, D., Zhang, B., Zhu, L., Zheng, L., Li, S., Tan, X., . . . Li, H. (2022). A Simple and Rapid Light-Initiated Chemiluminescence Assay for Quantitation of Artemisia-Specific Immunoglobulin E. Int Arch Allergy Immunol, 183(5), 490-497. doi:10.1159/000520511
211. Liu, F., Zhang, J., Liu, Y., Zhang, N., Holtappels, G., Lin, P., . . . Bachert, C. (2010). Inflammatory profiles in nasal mucosa of patients with persistent vs intermittent allergic rhinitis. Allergy, 65(9), 1149-1157. doi:10.1111/j.1398-9995.2010.02340.x
212. Liu, G., Huang, X., & Li, H. (1998). [A survey of main allergic pollen in pollinosis in district of Wuhan]. Lin Chuang Er Bi Yan Hou Ke Za Zhi, 12(5), 226-227, 230.
213. Liu, G., Zhou, X., Chen, J., & Liu, F. (2018). Oral Antihistamines Alone vs in Combination with Leukotriene Receptor Antagonists for Allergic Rhinitis: A Meta-analysis. Otolaryngol Head Neck Surg, 158(3), 450-458. doi:10.1177/0194599817752624
214. Liu, G. H., Zhu, R. F., Zhang, W., Li, W. J., Wang, Z. X., & Chen, H. (2008). Survey of airborne pollen in Hubei province of China. Chin Med Sci J, 23(4), 212-217. doi:10.1016/s1001-9294(09)60041-9
215. Liu, J., Liu, L., Cui, Y., Zhang, J., & Jiang, H. (2010). p38 MAPK regulates Th2 cytokines release in PBMCs in allergic rhinitis rats. J Huazhong Univ Sci Technolog Med Sci, 30(2), 222-225. doi:10.1007/s11596-010-0218-x
216. Liu, J. H., Yin, R., Tang, Y. Q., Tang, Q., & Zong, H. (2023). Variations of pollen concentration in urban alleys of park city and the transmission pattern during the period of high allergenic risk: Taking Qingyang District of Chengdu City as an example. Ying Yong Sheng Tai Xue Bao, 34(11), 3105-3113. doi:10.13287/j.1001-9332.202311.021
217. Liu, L., Li, G., Sun, Y., Li, J., Tang, N., & Dong, L. (2015). Airway wall thickness of allergic asthma caused by weed pollen or house dust mite assessed by computed tomography. Respir Med, 109(3), 339-346. doi:10.1016/j.rmed.2014.11.011
218. Liu, L., Wang, W., Sun, Y., & Li, G. (2023). Allergen Differences and Correlation Analysis in Siblings Diagnosed with Respiratory Allergic Diseases. Int Arch Allergy Immunol, 184(12), 1165-1172. doi:10.1159/000533331
219. Liu, L., Zhou, P., Wang, Z., Zhai, S., & Zhou, W. (2022). Efficacy and Safety of Omalizumab for the Treatment of Severe or Poorly Controlled Allergic Diseases in Children: A Systematic Review and Meta-Analysis. Front Pediatr, 10, 851177. doi:10.3389/fped.2022.851177
220. Liu, Q., & Guo, Z. (2009). Molecular cloning and characterization of a profilin gene BnPFN from Brassica nigra that expressing in a pollen-specific manner. Mol Biol Rep, 36(1), 135-139. doi:10.1007/s11033-007-9161-8
221. Liu, S., Guan, K., & Yin, J. (2023). [Research progress on pollen food allergy syndrome]. Zhonghua Yu Fang Yi Xue Za Zhi, 57(9), 1336-1341. doi:10.3760/cma.j.cn112150-20230705-00511
222. Liu, W., Xia, W., Fan, Y., Wang, H., Zuo, K., Lai, Y., . . . Xu, G. (2012). Elevated serum osteopontin level is associated with blood eosinophilia and asthma comorbidity in patients with allergic rhinitis. J Allergy Clin Immunol, 130(6), 1416-1418.e1416. doi:10.1016/j.jaci.2012.06.010
223. Liu, X., Ren, Y., Sun, X., Huang, H., & Liu, X. (2019). Bioinformatics-Based Approaches Predict That MIR-17-5P Functions in the Pathogenesis of Seasonal Allergic Rhinitis Through Regulating ABCA1 and CD69. Am J Rhinol Allergy, 33(3), 269-276. doi:10.1177/1945892418823388
224. Liu, X., Xiao, X., Xia, L., Yang, P. C., Liu, Z., & Li, L. (2014). Identification and characterization of a pan-allergen profilin, a major allergen from Caryota mitis pollen. Asian Pac J Allergy Immunol, 32(1), 39-45. doi:10.12932/ap0269.32.1.2014
225. Liu, X., Xing, Z., & Gao, Z. (2005). [The effect of antianaphylaxis drugs on specific IgE and eosinophil in serum of patients with allergic rhinitis]. Lin Chuang Er Bi Yan Hou Ke Za Zhi, 19(8), 337-339.
226. Liu, Y., & Cheng, L. (2008). [Observation on cardiovascular safety of loratadine in treatment of persistent allergic rhinitis]. Lin Chuang Er Bi Yan Hou Tou Jing Wai Ke Za Zhi, 22(23), 1076-1078.
227. Liu, Y., Jiang, P., Chen, X., Zhang, W., & Shi, J. (2022). Efficacy and Safety of Rupatadine Fumarate Combined with Acupoint Application in Allergic Rhinitis Complicated with Diabetes. Comput Intell Neurosci, 2022, 6935758. doi:10.1155/2022/6935758
228. Liu, Y., Liu, S., Lu, S. F., & Zang, G. M. (2023). Analysis of the efficacy of azelastine nasal spray combined with mussel mucin in the treatment of allergic rhinitis and the influence of peripheral blood CCL26 and CCR3 levels. Eur Rev Med Pharmacol Sci, 27(20), 9738-9746. doi:10.26355/eurrev_202310_34145
229. Liu, Y., Lu, C., Li, Y., Norbäck, D., & Deng, Q. (2022). Outdoor Air Pollution and Indoor Window Condensation Associated with Childhood Symptoms of Allergic Rhinitis to Pollen. Int J Environ Res Public Health, 19(13). doi:10.3390/ijerph19138071
230. Liu, Y., Peng, J., Zhou, Y., & Cui, Y. (2017). Comparison of atopy patch testing to skin prick testing for diagnosing mite-induced atopic dermatitis: a systematic review and meta-analysis. Clin Transl Allergy, 7, 41. doi:10.1186/s13601-017-0178-3
231. Liu, Y., Shi, J., & Chen, X. (2015). Identification of novel targets for seasonal allergic rhinitis during and outside the pollen season by microarray analysis. Acta Otolaryngol, 135(12), 1330-1336. doi:10.3109/00016489.2015.1067906
232. Liu, Y., Sun, X., Wang, G., Tao, A., Wu, Y., Li, M., . . . Xie, M. (2015). Expression, purification and identification of Pla a1 in a codon-optimized Platanus pollen allergen. Mol Med Rep, 12(2), 2197-2202. doi:10.3892/mmr.2015.3652
233. Liu, Y., Ye, X. J., Zhao, C. L., & Ji, Q. (2016). [The effect of combined therapy on seasonal allergic rhinitis]. Lin Chuang Er Bi Yan Hou Tou Jing Wai Ke Za Zhi, 30(13), 1049-1052. doi:10.13201/j.issn.1001-1781.2016.13.011
234. Liu, Z. G., Song, J. J., & Kong, X. L. (2010). A study on pollen allergens in China. Biomed Environ Sci, 23(4), 319-322. doi:10.1016/s0895-3988(10)60070-0
235. Liu, Z. G., Yu, H. M., Wen, S. L., & Liu, Y. L. (2005). [Histopathological study on allergic rhinitis treated with Centipeda minima]. Zhongguo Zhong Yao Za Zhi, 30(4), 292-294.
236. Lou, H., Huang, Y., Chu, X., Wang, Y., Wang, C., & Zhang, L. (2023). M2 Macrophages Upregulated by Allergen Exposure in Seasonal Allergic Rhinitis. Int Arch Allergy Immunol, 184(6), 587-597. doi:10.1159/000529436
237. Lou, H., Huang, Y., Ouyang, Y., Zhang, Y., Xi, L., Chu, X., . . . Zhang, L. (2020). Artemisia annua-sublingual immunotherapy for seasonal allergic rhinitis: A randomized controlled trial. Allergy, 75(8), 2026-2036. doi:10.1111/all.14218
238. Lou, H., Ma, S., Zhao, Y., Cao, F., He, F., Liu, Z., . . . Bachert, C. (2017). Sensitization patterns and minimum screening panels for aeroallergens in self-reported allergic rhinitis in China. Sci Rep, 7(1), 9286. doi:10.1038/s41598-017-10111-9
239. Lou, H., Wang, X., Wei, Q., Zhao, C., Xing, Z., Zhang, Q., . . . Zhang, L. (2020). Artemisia Annua sublingual immunotherapy for seasonal allergic rhinitis: A multicenter, randomized trial. World Allergy Organ J, 13(9), 100458. doi:10.1016/j.waojou.2020.100458
240. Lu, D., Zhao, Y., Zheng, Y., An, P., Wang, L., Qiao, X., & Wang, D. (2011). Evaluation of quality of life questionnaires for adult patients with moderate to severe allergic rhinitis. Am J Otolaryngol, 32(6), 494-498. doi:10.1016/j.amjoto.2010.09.014
241. Lu, D., Zhau, P., Yu, Q., & Zhang, C. (1994). Airway provocation test with ragweed pollen extract in Chinese asthmatics. Asian Pac J Allergy Immunol, 12(2), 125-129.
242. Lu, D. W. (1992). [Investigations on ragweed pollens in the air in Qingdao District and its allergenicity]. Zhonghua Yu Fang Yi Xue Za Zhi, 26(4), 216-218.
243. Lu, H. G., Peng, H., Chen, D. H., Li, T. Y., & Shi, J. B. (2011). [Relationship between allergic symptoms and RORC2 and IL-17 in patients with allergic rhinitis]. Zhonghua Er Bi Yan Hou Tou Jing Wai Ke Za Zhi, 46(2), 144-148.
244. Lu, J., Li, M., Sun, X., Zhang, Y., Liu, Y., Xu, J., & Zhang, S. (2014). [Foxp3(+)Treg cells mediate immune protection of Humulus pollen allergy DNA vaccine pcDNA3.1-Hum in asthmatic mice]. Nan Fang Yi Ke Da Xue Xue Bao, 34(1), 14-19.
245. Lu, K., He, Y. M., Mao, W., Du, Z. Y., Wang, L. J., Liu, G. M., . . . Duan, Y. Z. (2020). [Potential geographical distribution and changes of Artemisia ordosica in China under future climate change]. Ying Yong Sheng Tai Xue Bao, 31(11), 3758-3766. doi:10.13287/j.1001-9332.202011.017
246. Lu, L. X., Bao, J. Y., Wang, J. Y., Liu, S. X., Chen, J., Tian, L., . . . Jie, Y. (2021). [The prevalence of allergic conjunctivitis based on outpatients in Baotou area]. Zhonghua Yu Fang Yi Xue Za Zhi, 55(5), 627-632. doi:10.3760/cma.j.cn112150-20210318-00266
247. Lu, S., Yao, C., Zhou, S., Lin, Y., Zhang, L., Zeng, J., . . . Wang, Q. (2020). Studies on relationships between air pollutants and allergenicity of Humulus Scandens pollen collected from different areas of Shanghai. J Environ Sci (China), 95, 43-48. doi:10.1016/j.jes.2020.03.037
248. Lü, S. L., Wang, Q. Y., Wu, M. H., Feng, M., Shinichi, N., Wang, X. J., & Shinichi, Y. (2010). [Perspectives on synergic biological effects induced by ambient allergenic pollen and urban fine/ultrafine particulate matters in atmosphere]. Huan Jing Ke Xue, 31(9), 2260-2266.
249. Lu, Y., Sun, L., Li, C., Wang, X., Li, W., Zhao, T., . . . Wang, Z. (2022). Comparative mass spectrometry analysis of N-glycans from the glycoproteins of eight allergy-inducing plants. Food Chem, 384, 132440. doi:10.1016/j.foodchem.2022.132440
250. Lu, Y., Yin, M., & Cheng, L. (2014). [Meta-analysis of leukotriene receptor antagonist montelukast in the treatment of allergic rhinitis]. Zhonghua Er Bi Yan Hou Tou Jing Wai Ke Za Zhi, 49(8), 659-667.
251. Luo, H., Tao, Z., Yan, N., Liang, J., Wang, P., & Wang, J. (2009). [Clinical research of Ibudilast on treating the steroid resistant allergic rhinitis]. Lin Chuang Er Bi Yan Hou Tou Jing Wai Ke Za Zhi, 23(2), 63-66.
252. Luo, R., Zhong, L. L., Yi, H. L., Tan, Y. P., Chen, M., & Li, Y. (2012). [Results of skin prick test in young children with wheezing or allergic diseases]. Zhongguo Dang Dai Er Ke Za Zhi, 14(4), 282-284.
253. Luo, W., Hou, X., Sun, Y., Zhang, H., Ren, H., Ma, X., . . . Sun, B. (2023). Developing and validating clinical models to identify candidates for allergic rhinitis pre-exposure prophylaxis. Ann Med, 55(2), 2287188. doi:10.1080/07853890.2023.2287188
254. Luo, W., Huang, H., Zheng, P., Wei, N., Luo, J., Sun, B., & Zeng, G. (2016). Major grass pollen allergens and components detected in a southern Chinese cohort of patients with allergic rhinitis and/or asthma. Mol Immunol, 78, 105-112. doi:10.1016/j.molimm.2016.08.013
255. Luo, W., Huang, H., Zheng, P., Zheng, J., & Sun, B. (2021). CCD Inhibition Test Can Improve the Accuracy of the Detection of Pollen and Seed Food Allergen-Specific IgE in Southern China. J Asthma Allergy, 14, 439-447. doi:10.2147/jaa.S302920
256. Luo, W., Li, Y., Xu, L., Yu, Y., Ma, J., Wang, Y., . . . Sun, B. (2023). Pollen allergens sensitization characteristics and risk factors among allergy rhinitis of children in mainland China: A multicenter study. Heliyon, 9(4), e14914. doi:10.1016/j.heliyon.2023.e14914
257. Luo, W., Pan, G., Huang, H., Zheng, P., Wei, N., Zhang, Y., . . . Sun, B. (2017). A Component-resolved Diagnostic Approach for a Study on Grass Pollen Allergens in Chinese Southerners with Allergic Rhinitis and/or Asthma. J Vis Exp(124). doi:10.3791/55723
258. Luo, W., Yang, S., Huang, H., Wu, L., Cheng, Z. J., Zheng, P., . . . Sun, B. (2021). Analysis of Peanut Allergen Components Sensitization and Cross Reaction with Pollen Allergen in Chinese Southerners with Allergic Rhinitis and/or Asthma. J Asthma Allergy, 14, 1285-1293. doi:10.2147/jaa.S335265
259. Lv, X., Xi, L., Han, D., & Zhang, L. (2010). Evaluation of the psychological status in seasonal allergic rhinitis patients. ORL J Otorhinolaryngol Relat Spec, 72(2), 84-90. doi:10.1159/000297576
260. Ma, L., Chen, D. L., Zhang, R. X., Wang, X. L., Shi, Y. J., Ji, C., . . . Guan, P. (2007). [Genetic epidemiological study on allergic rhinitis in Nantong region of Jiangsu Province]. Zhonghua Er Bi Yan Hou Tou Jing Wai Ke Za Zhi, 42(9), 643-646.
261. Ma, R., Shen, Y., Hou, L., Yang, Z., Feng, N., Yan, X., . . . Di, L. (2014). [The correlation of allergic rhinitis and trace elements in Ningxia region]. Zhonghua Er Bi Yan Hou Tou Jing Wai Ke Za Zhi, 49(12), 1017-1020.
262. Ma, S., & Yin, J. (2014). Imbalance of serum IL-10 and TGF-β in patients with pollen food syndrome. Allergol Immunopathol (Madr), 42(3), 198-205. doi:10.1016/j.aller.2013.01.004
263. Ma, S., Yin, J., & Jiang, N. (2013). Component-resolved diagnosis of peach allergy and its relationship with prevalent allergenic pollens in China. J Allergy Clin Immunol, 132(3), 764-767. doi:10.1016/j.jaci.2013.04.017
264. Ma, T., Chen, Y., Pang, Y., Wang, X., Dai, D., Zhuang, Y., . . . Zhang, L. (2021). Prevalence and risk factors of allergic rhinitis and asthma in the southern edge of the plateau grassland region of northern China: A cross-sectional study. World Allergy Organ J, 14(7), 100537. doi:10.1016/j.waojou.2021.100537
265. Ma, T., Wang, H., & Wang, X. (2021). Effectiveness and Response Predictors of Omalizumab in Treating Patients with Seasonal Allergic Rhinitis: A Real-World Study. J Asthma Allergy, 14, 59-66. doi:10.2147/jaa.S288952
266. Ma, T., Wang, X., Zhuang, Y., Shi, H., Ning, H., Lan, T., . . . Zhang, L. (2020). Prevalence and risk factors for allergic rhinitis in adults and children living in different grassland regions of Inner Mongolia. Allergy, 75(1), 234-239. doi:10.1111/all.13941
267. Ma, T., Wang, Z., He, N., Chen, Y., Lan, T., Wang, H., . . . Wang, X. (2022). [Analysis on the allergic characteristics of humulus pollen in Beijing]. Lin Chuang Er Bi Yan Hou Tou Jing Wai Ke Za Zhi, 36(1), 41-44;50. doi:10.13201/j.issn.2096-7993.2022.01.009
268. Ma, T., Zhuang, Y., Wang, H., Wei, Q., Shi, H., Ning, H., . . . Wang, X. (2020). [Analysis of sensitization characteristics of artemisia pollen in the Inner Mongolian grassland region of China]. Lin Chuang Er Bi Yan Hou Tou Jing Wai Ke Za Zhi, 34(12), 1092-1096. doi:10.13201/j.issn.2096-7993.2020.12.009
269. Ma, T. T., Cao, M. D., Yu, R. L., Shi, H. Y., Yan, W. J., Liu, J. G., . . . Yin, J. S. (2020). Leukotriene A(4) Hydrolase Is a Candidate Predictive Biomarker for Successful Allergen Immunotherapy. Front Immunol, 11, 559746. doi:10.3389/fimmu.2020.559746
270. Ma, T. T., He, N., Wang, H. T., Chen, Y. L., Zhuang, Y., Shi, H. Y., . . . Wang, X. Y. (2022). [Sensitization characteristics of Juniperus chinensis pollen in Beijing area]. Zhonghua Er Bi Yan Hou Tou Jing Wai Ke Za Zhi, 57(4), 479-484. doi:10.3760/cma.j.cn115330-20210701-00416
271. Ma, T. T., Zhuang, Y., Gong, H. Y., Yii, A. C., Wang, X. Y., & Shi, H. Z. (2017). Predictive value of respiratory symptoms for the diagnosis of pollen-induced seasonal asthma among children and adults in Inner Mongolia. Ther Clin Risk Manag, 13, 967-974. doi:10.2147/tcrm.S138355
272. Ma, T. T., Zhuang, Y., Shi, H. Y., Ning, H. Y., Guo, M. Y., He, H., . . . Wang, X. Y. (2019). [Epidemiology of allergic rhinitis in children in grassland of Inner mongolia]. Zhonghua Er Bi Yan Hou Tou Jing Wai Ke Za Zhi, 54(8), 571-575. doi:10.3760/cma.j.issn.1673-0860.2019.08.003
273. Ma, T. T., Zhuang, Y., Shi, H. Y., Ning, H. Y., Kang, Z. X., Siqin, Q. B. T., . . . Wang, X. Y. (2021). [Analysis of characteristics of Chenopodiaceae pollen induced seasonal allergic rhinitis in the Inner Mongolian grassland region of China]. Zhonghua Er Bi Yan Hou Tou Jing Wai Ke Za Zhi, 56(5), 471-477. doi:10.3760/cma.j.cn115330-20200713-00584
274. Ma, W. J., Bao, M. J., Zhu, J. P., Yao, H. Y., Xie, Y. C., Guan, Y., . . . Xie, Q. M. (2012). Oral administration of allergen extracts from mugwort pollen desensitizes specific allergen-induced allergy in mice. Vaccine, 30(8), 1437-1444. doi:10.1016/j.vaccine.2012.01.005
275. Mao, D., Tang, R., Wu, R., Hu, H., Sun, L. J., Zhu, H., . . . Han, J. G. (2017). Prevalence trends in the characteristics of patients with allergic asthma in Beijing, 1994 to 2014. Medicine (Baltimore), 96(22), e7077. doi:10.1097/md.0000000000007077
276. Meng, C. D., Dong, Z., Sha, J. C., Li, L., & Zhu, D. D. (2012). [Analysis of the level and significance of immunoglobulin free light chain in nasal secretion and in serum of patients with allergic rhinitis and non-allergic rhinitis]. Zhonghua Er Bi Yan Hou Tou Jing Wai Ke Za Zhi, 47(11), 908-912.
277. Meng, C. D., Li, L., Jiang, X. D., Dong, Z., & Zhu, D. D. (2010). [Clinical characteristics in patients with non-allergic rhinitis and allergic rhinitis: preliminary analysis]. Zhonghua Er Bi Yan Hou Tou Jing Wai Ke Za Zhi, 45(12), 999-1002.
278. Meng, G., Li, C., Cai, Q., & Xie, C. (2005). [Survey on pollen sensitization of tropic plants in Haikou]. Lin Chuang Er Bi Yan Hou Ke Za Zhi, 19(22), 1044-1045.
279. Meng, L., Yang, L., Wang, Q. H., & Wang, J. J. (2023). [Distribution, hazard evaluation, and control measures of allergenic airborne pollen]. Ying Yong Sheng Tai Xue Bao, 34(10), 2845-2853. doi:10.13287/j.1001-9332.202310.001
280. Miao, Q., Wang, Y., Liu, Y. G., Ren, Y. X., Guan, H., Li, Z., . . . Xiang, L. (2018). Seasonal variation in circulating group 2 innate lymphoid cells in mugwort-allergic asthmatics during and outside pollen season. Allergy Asthma Clin Immunol, 14, 6. doi:10.1186/s13223-018-0229-x
281. Miao, Q., Xiang, L., Guan, H., Liu, Y., Li, Z., Ren, Y., & Xu, W. (2020). Factors related to disagreement between self-reported versus objective measurement of allergen sensitization at a tertiary pediatric center in Beijing, China. BMC Pediatr, 20(1), 259. doi:10.1186/s12887-020-02148-z
282. Mou, Z., Shi, J., Tan, Y., Xu, R., Zhao, Z., Xu, G., & Li, H. (2010). Association between TIM-1 gene polymorphisms and allergic rhinitis in a Han Chinese population. J Investig Allergol Clin Immunol, 20(1), 3-8.
283. Mou, Z., Xia, J., Tan, Y., Wang, X., Zhang, Y., Zhou, B., . . . Han, D. (2009). Overexpression of thymic stromal lymphopoietin in allergic rhinitis. Acta Otolaryngol, 129(3), 297-301. doi:10.1080/00016480802225884
284. Ni, W. W., Huang, W., Wu, D. Q., Zhou, Y. J., Ji, C. M., Cao, M. D., . . . Wei, J. F. (2017). Expression and purification of a major allergen, Pla a 1, from Platanus acerifolia pollen and the preparation of its monoclonal antibody. Mol Med Rep, 16(3), 2887-2892. doi:10.3892/mmr.2017.6899
285. Ni, W. W., Wang, L. B., Zhou, Y. J., Cao, M. D., Huang, W., Guo, M., . . . Wei, J. F. (2017). Expression, purification and epitope analysis of Pla a 3 allergen from Platanus acerifolia pollen. Mol Med Rep, 16(3), 2851-2855. doi:10.3892/mmr.2017.6867
286. Ning, H. Y., Cai, H. J., Ma, T. T., Fan, C. E., Wu, D. D., Gao, F. Y., . . . Wang, X. Y. (2023). [Investigation and analysis of airborne allergenic pollen in 4 districts and 5 counties of Hohhot City]. Zhonghua Yu Fang Yi Xue Za Zhi, 57(9), 1364-1372. doi:10.3760/cma.j.cn112150-20230116-00034
287. Ning, X., Kuang, Y., Zhao, S., Hou, W., Yang, G., Zhu, X., . . . Huang, J. (2020). Design of an Optimally-Diagnostic Skin Test for Diagnosis of Sensitivity to Eight Allergens: A First-in-Human Study of Dose Escalation and Simultaneous Administration in Chinese Subjects. J Asthma Allergy, 13, 471-481. doi:10.2147/jaa.S276720
288. Ning, X., Xing, Z., Wang, M., & Gao, Z. (2011). [To investigate the effect of pretreatment with budesonide spray in patients with allergic rhinitis]. Lin Chuang Er Bi Yan Hou Tou Jing Wai Ke Za Zhi, 25(14), 633-635.
289. Niu, X., Yin, X., Wu, X., Zhang, Q., Jiang, Y., He, J., . . . Wang, R. (2023). Heat-Killed Bifidobacterium longum BBMN68 in Pasteurized Yogurt Alleviates Mugwort Pollen-Induced Allergic Airway Responses through Gut Microbiota Modulation in a Murine Model. Foods, 12(10). doi:10.3390/foods12102049
290. Ouyang, Y., Fan, E., Li, Y., & Zhang, L. (2014). [Onset feature and efficacy of early interventional treatment of Artemisia pollinosis]. Zhonghua Er Bi Yan Hou Tou Jing Wai Ke Za Zhi, 49(4), 272-276.
291. Ouyang, Y., Li, J., Zhang, D., Fan, E., Li, Y., & Zhang, L. (2017). A model to predict the incidence of allergic rhinitis based on meteorological factors. Sci Rep, 7(1), 10006. doi:10.1038/s41598-017-10721-3
292. Ouyang, Y., Li, Y., Xu, Z., An, Y., & Zhang, L. (2021). Assessment of changes in genetic transcriptome in nasal epithelial cells exposed to ozone-aged black carbon and pollen allergen by high-throughput transcriptomics. Allergy Asthma Clin Immunol, 17(1), 52. doi:10.1186/s13223-021-00553-2
293. Ouyang, Y., Xu, Z., Fan, E., Li, Y., & Zhang, L. (2016). Effect of nitrogen dioxide and sulfur dioxide on viability and morphology of oak pollen. Int Forum Allergy Rhinol, 6(1), 95-100. doi:10.1002/alr.21632
294. Ouyang, Y., Yin, Z., Li, Y., Fan, E., & Zhang, L. (2019). Associations among air pollutants, grass pollens, and daily number of grass pollen allergen-positive patients: a longitudinal study from 2012 to 2016. Int Forum Allergy Rhinol, 9(11), 1297-1303. doi:10.1002/alr.22389
295. Ouyang, Y. H., Zhang, D. S., Fan, E. Z., Li, Y., & Zhang, L. (2012). [Correlation between symptoms of pollen allergic rhinitis and pollen grain spreading in summer and autumn]. Zhonghua Er Bi Yan Hou Tou Jing Wai Ke Za Zhi, 47(8), 623-627.
296. Ouyang, Z. Y., Xin, J. N., Zheng, H., Meng, X. S., & Wang, X. K. (2007). [Species composition, distribution and phenological characters of pollen-allergenic plants in Beijing urban area]. Ying Yong Sheng Tai Xue Bao, 18(9), 1953-1958.
297. Pan, J., Liu, X., Chen, D., Zhao, W., Tao, J., & Wu, S. (2023). Effect of refined management on the recovery of patients undergoing sinusitis surgery via nasal endoscopy. Am J Transl Res, 15(1), 522-530.
298. Pang, C., Bian, S. N., Liu, C. H., Guo, L. L., Cui, Y., Lin, F., . . . Guan, K. (2021). [The characteristics and change of aeroallergens in children from 2015 to 2020 in a hospital of pediatric in Beijing]. Zhonghua Yu Fang Yi Xue Za Zhi, 55(7), 840-846. doi:10.3760/cma.j.cn112150-20210506-00441
299. Pang, K., Li, G., Li, M., Zhang, L., Fu, Q., Liu, K., . . . Zhang, Q. (2022). Prevalence and Risk Factors for Allergic Rhinitis in China: A Systematic Review and Meta-Analysis. Evid Based Complement Alternat Med, 2022, 7165627. doi:10.1155/2022/7165627
300. Peng, J., Wu, X., Hu, J., Fang, Y., Zi, M., & Liu, B. (2012). Influencing factors on efficacy of summer acupoint application treatment for allergic rhinitis: a retrospective study. J Tradit Chin Med, 32(3), 377-381. doi:10.1016/s0254-6272(13)60041-2
301. Qian, F. H., Zhang, Q., Zhou, L. F., Jin, G. F., Bai, J. L., & Yin, K. S. (2010). Polymorphisms in the toll-like receptor 2 subfamily and risk of asthma: a case-control analysis in a Chinese population. J Investig Allergol Clin Immunol, 20(4), 340-346.
302. Qing, M., Wei, X., Zhen, L., Hui, G., Xiao-Ying, L., Hui-Jie, H., . . . Li, X. (2017). Influence of Sensitization Patterns on Fractional Exhaled Nitric Oxide in Asthmatic Children. Iran J Allergy Asthma Immunol, 16(1), 53-59.
303. Qiu, H., Zheng, R., Wang, X., Chen, Z., Feng, P., Huang, X., . . . Yang, Q. (2021). Using the Internet Big Data to Investigate the Epidemiological Characteristics of Allergic Rhinitis and Allergic Conjunctivitis. Risk Manag Healthc Policy, 14, 1833-1841. doi:10.2147/rmhp.S307247
304. Qiu, Q., Xu, M., Lu, C., Chen, J., Chen, S., Kong, W., & Han, H. (2016). Safety and efficacy of rush allergen-specific immunotherapy in Chinese allergic rhinitis patients. Int J Immunopathol Pharmacol, 29(4), 720-725. doi:10.1177/0394632016659301
305. Qu, S. H., Li, T. Y., Ou, Z. Y., Lin, Z. B., Chen, Y. Q., & Xu, G. (2005). [Expression of T-bet and its relation with IgE and eosinophil cationic protein in patients with allergic rhinitis]. Zhonghua Er Bi Yan Hou Tou Jing Wai Ke Za Zhi, 40(12), 908-911.
306. Sha, J. C., Zhu, D. D., Dong, Z., Jiang, X. D., Li, L., Zhu, X. W., . . . Wang, Q. (2011). [Survey on clinical characteristics of pediatric allergic rhinitis]. Zhonghua Er Bi Yan Hou Tou Jing Wai Ke Za Zhi, 46(1), 26-30.
307. Shaoqing, Y., Ruxin, Z., Guojun, L., Zhiqiang, Y., Hua, H., Shudong, Y., & Jie, Z. (2011). Microarray analysis of differentially expressed microRNAs in allergic rhinitis. Am J Rhinol Allergy, 25(6), e242-246. doi:10.2500/ajra.2011.25.3682
308. Shen, H. D., Chang, L. Y., Su, S. N., & Han, S. H. (1988). Characteristics of five monoclonal antibodies to major allergens of the short ragweed pollen. Int Arch Allergy Appl Immunol, 85(2), 167-173. doi:10.1159/000234497
309. Shen, H. D., Wang, S. R., Tang, R. B., Chang, Z. N., Su, S. N., & Han, S. H. (1988). Identification of allergens and antigens of Bermuda grass (Cynodon dactylon) pollen by immunoblot analysis. Clin Allergy, 18(4), 401-409. doi:10.1111/j.1365-2222.1988.tb02888.x
310. Shen, L., Hu, X. X., Zeng, L., Liu, Y. H., Wu, Y., Yi, H. R., . . . Ye, J. (2023). [Preliminary analysis of seasonal pollen allergens of allergic rhinitis in a hospital of Nanchang City]. Zhonghua Yu Fang Yi Xue Za Zhi, 57(12), 1988-1995. doi:10.3760/cma.j.cn112150-20230529-00415
311. Shen, Z., Zhang, P., Kang, W., Chen, X., Li, H., Shao, Y., . . . Quan, F. (2023). Clinical efficacy in one-year treatment with Artemisia annua-SLIT drops in monosensitized and polysensitized individuals. Am J Otolaryngol, 44(6), 104002. doi:10.1016/j.amjoto.2023.104002
312. Shi, H. Y., Pan, C., Ma, T. T., Chen, Y. L., Yan, W. J., Liu, J. G., . . . Wei, J. F. (2020). Clinical Efficacy Evaluation of 1-Year Subcutaneous Immunotherapy for Artemisia sieversiana Pollen Allergic Rhinitis by Serum Metabolomics. Front Pharmacol, 11, 305. doi:10.3389/fphar.2020.00305
313. Shi, J., Zhang, Y., Qi, S., Liu, G., Dong, X., Huang, N., . . . Zhu, B. (2017). Identification of potential crucial gene network related to seasonal allergic rhinitis using microarray data. Eur Arch Otorhinolaryngol, 274(1), 231-237. doi:10.1007/s00405-016-4197-9
314. Shi, M., Yao, L., Yang, X., Chen, J., Zhang, L., Sun, J., & Xu, X. (2016). [Analysis of allergens characteristic in 1172 patients with allergic rhinitis in Changzhou area]. Lin Chuang Er Bi Yan Hou Tou Jing Wai Ke Za Zhi, 30(1), 54-57.
315. Shumin, Z., Luying, Z., Senlin, L., Jiaxian, P., Yang, L., Lanfang, R., . . . Qingyue, W. (2021). Ambient particulate matter-associated autophagy alleviates pulmonary inflammation induced by Platanus pollen protein 3 (Pla3). Sci Total Environ, 758, 143696. doi:10.1016/j.scitotenv.2020.143696
316. Song, L. B., Jiao, Y. X., Xu, Z. Q., Zhu, D. X., Yang, Y. S., Wei, J. F., . . . Lu, Y. (2023). Identification of Pla a 7 as a novel pollen allergen group in Platanus acerifolia pollen. Int Immunopharmacol, 125(Pt A), 111160. doi:10.1016/j.intimp.2023.111160
317. Song, T. T., Jing, X. H., Guo, W., Han, G. X., Du, S., & Zhao, J. P. (2023). [Acupuncture for prevention of moderate to severe seasonal allergic rhinitis: a randomized controlled trial]. Zhongguo Zhen Jiu, 43(2), 123-127. doi:10.13703/j.0255-2930.20220414-k0004
318. Song, X., Liu, C. H., Wang, W., Huang, G. M., Zhao, J., & Sha, L. (2022). [Characteristics and changes of sensitization patterns of major allergens in children from 2010 to 2020 in a hospital of pediatric in Beijing]. Zhonghua Yu Fang Yi Xue Za Zhi, 56(6), 763-773. doi:10.3760/cma.j.cn112150-20220321-00266
319. Song, Y., & Yan, Z. (2018). Exploring of the molecular mechanism of rhinitis via bioinformatics methods. Mol Med Rep, 17(2), 3014-3020. doi:10.3892/mmr.2017.8213
320. Su, S. N., Lau, G. X., Tsai, J. J., Yang, S. Y., Shen, H. D., & Han, S. H. (1991). Isolation and partial characterization of Bermuda grass pollen allergen, BG-60a. Clin Exp Allergy, 21(4), 449-455. doi:10.1111/j.1365-2222.1991.tb01685.x
321. Su, S. N., Shu, P., Lau, G. X., Yang, S. Y., Huang, S. W., & Lee, Y. C. (1996). Immunologic and physicochemical studies of Bermuda grass pollen antigen BG60. J Allergy Clin Immunol, 98(3), 486-494. doi:10.1016/s0091-6749(96)70080-5
322. Su, W., Wan, Q., Huang, J., Han, L., Chen, X., Chen, G., . . . Liang, D. (2015). Culture medium from TNF-α-stimulated mesenchymal stem cells attenuates allergic conjunctivitis through multiple antiallergic mechanisms. J Allergy Clin Immunol, 136(2), 423-432.e428. doi:10.1016/j.jaci.2014.12.1926
323. Sun, B. Q., Lai, X. X., Gjesing, B., Spangfort, M. D., & Zhong, N. S. (2010). Prevalence of sensitivity to cockroach allergens and IgE cross-reactivity between cockroach and house dust mite allergens in Chinese patients with allergic rhinitis and asthma. Chin Med J (Engl), 123(24), 3540-3544.
324. Sun, L., Xu, Y., Wang, Y., Lou, Y., Xu, Y., & Guo, Y. (2017). Species and quantity of airborne pollens in Shanghai as monitored by gravitational and volumetric methods. Asian Pac J Allergy Immunol, 35(1), 38-45. doi:10.12932/ap0743
325. Sun, L. W., Liu, Z. Y., Sha, J. C., Meng, C. D., & Zhu, D. D. (2022). [Bioinformatics analysis of nasal epithelial cell gene expression in seasonal and perennial allergic rhinitis]. Zhonghua Er Bi Yan Hou Tou Jing Wai Ke Za Zhi, 57(4), 425-432. doi:10.3760/cma.j.cn115330-20210630-00397
326. Suo, S., Ma, T., Wang, H., Wang, Y., & Wang, X. (2023). [Sensitization characteristics of ragweed pollen in Beijing area]. Lin Chuang Er Bi Yan Hou Tou Jing Wai Ke Za Zhi, 37(5), 380-386. doi:10.13201/j.issn.2096-7993.2023.05.012
327. Tang, H., Wang, H., Bai, J., Ding, M., Liu, W., Xia, W., . . . Fang, J. (2012). Sensitivity of MUC5AC to topical corticosteroid is negatively associated with interleukin-17A in patients with allergic rhinitis. Am J Rhinol Allergy, 26(5), 359-364. doi:10.2500/ajra.2012.26.3795
328. Tang, R., Lei, S., Zhu, L., Lv, Y., & Li, H. (2022). Prevention of omalizumab for seasonal allergic rhinoconjunctivitis: a retrospective cohort study. Front Immunol, 13, 913424. doi:10.3389/fimmu.2022.913424
329. Tang, R., Sun, J. L., Yin, J., & Li, Z. (2015). Artemisia allergy research in China. Biomed Res Int, 2015, 179426. doi:10.1155/2015/179426
330. Tang, R. B., & Wu, K. K. (1989). Total serum IgE, allergy skin testing, and the radioallergosorbent test for the diagnosis of allergy in asthmatic children. Ann Allergy, 62(5), 432-435.
331. Tang, S., Kang, H., Zhu, L., Chen, X., Li, J., Lu, X., . . . Xia, G. (2008). [Epidemiology investigation of allergic rhinitis in urban and rural areas of Ningbo and Yongzhou]. Lin Chuang Er Bi Yan Hou Tou Jing Wai Ke Za Zhi, 22(14), 642-644.
332. Tang, X. F., Tang, H. Y., Sun, L. D., Xiao, F. L., Zhang, Z., Li, Y., . . . Zhang, X. J. (2012). Genetic variant rs4982958 at 14q11.2 is associated with allergic rhinitis in a Chinese Han population running title: 14q11.2 is a susceptibility locus for allergic rhinitis. J Investig Allergol Clin Immunol, 22(1), 55-62.
333. Tang, Y. Q., Wu, X. Y., & Zong, H. (2022). [Allergenic risk analysis of street trees of urban alleys in Qingyang District, Chengdu, China]. Ying Yong Sheng Tai Xue Bao, 33(6), 1615-1621. doi:10.13287/j.1001-9332.202206.020
334. Tao, A. L., & He, S. H. (2004). Bridging PCR and partially overlapping primers for novel allergen gene cloning and expression insert decoration. World J Gastroenterol, 10(14), 2103-2108. doi:10.3748/wjg.v10.i14.2103
335. Tao, A. L., & He, S. H. (2005). Cloning, expression, and characterization of pollen allergens from Humulus scandens (Lour) Merr and Ambrosia artemisiifolia L. Acta Pharmacol Sin, 26(10), 1225-1232. doi:10.1111/j.1745-7254.2005.00194.x
336. Tao, A. L., He, S. H., Zhang, L. D., Chen, Z. Q., & Li, D. D. (2004). [Cloning full-length homologous cDNAs of pollen allergens in Humulus Scandens(Lour.) Merr by degenerate primer]. Xi Bao Yu Fen Zi Mian Yi Xue Za Zhi, 20(1), 99-103.
337. Tao, X. Y., Ng, C. L., Chen, D., Lin, Z. B., Wu, S. L., Liang, M. J., . . . Xu, R. (2018). Clinical Characteristics and Allergen Sensitization Patterns of Patients with Local Allergic Rhinitis in Southern China. Int Arch Allergy Immunol, 175(1-2), 107-113. doi:10.1159/000485896
338. Tao, Y., Yin, S., Fu, L., Wang, M., Meng, L., Li, F., . . . Li, Q. (2022). Identification of allergens and allergen hydrolysates by proteomics and metabolomics: A comparative study of natural and enzymolytic bee pollen. Food Res Int, 158, 111572. doi:10.1016/j.foodres.2022.111572
339. Teng, B., Zhang, X., Yi, C., Zhang, Y., Ye, S., Wang, Y., . . . Lu, B. (2017). The Association between Ambient Air Pollution and Allergic Rhinitis: Further Epidemiological Evidence from Changchun, Northeastern China. Int J Environ Res Public Health, 14(3). doi:10.3390/ijerph14030226
340. Tian, R., Yang, Y., Liu, L., Sun, Y., Tang, N., Zhang, Y., . . . Bachert, C. (2022). Seasonal distribution of inhaled allergens in allergic asthma patients with or without allergic rhinitis. Chin Med J (Engl), 135(15), 1867-1869. doi:10.1097/cm9.0000000000001894
341. Tong, P., Gao, L., Gao, J., Li, X., Wu, Z., Yang, A., & Chen, H. (2017). Iron-induced chelation alleviates the potential allergenicity of ovotransferrin in a BALB/c mouse model. Nutr Res, 47, 81-89. doi:10.1016/j.nutres.2017.09.009
342. Tong, S., Yin, Y., & Bao, Y. (2022). Climatotherapy for asthma: Research progress and prospect. Environ Res, 214(Pt 3), 113988. doi:10.1016/j.envres.2022.113988
343. Tsai, Y. T., Chen, S. H., Lin, K. L., & Hsieh, K. H. (1990). Rice pollen allergy in Taiwan. Ann Allergy, 65(6), 459-462.
344. Wang, C., Zhang, L., Han, D., Zhou, B., Zhao, Y., & Wang, X. (2006). [Prevalence of sensitization to aeroallergens in Beijing patients with allergic rhinitis]. Lin Chuang Er Bi Yan Hou Ke Za Zhi, 20(5), 204-207.
345. Wang, G., Peng, Y., Du, C., Tang, B., Liu, J., Zhen, X., . . . Jiao, X. (2002). [Epidemiological survey on bronchial asthma in Henan province]. Zhonghua Jie He He Hu Xi Za Zhi, 25(1), 25-28.
346. Wang, G., Xu, Z., Zhu, J., Ren, J., Chen, M., He, G., & Yu, B. (2020). Decreased Risk in the Pancreatic Cancer With History of Hay Fever: A Meta-Analysis. Front Public Health, 8, 551490. doi:10.3389/fpubh.2020.551490
347. Wang, H. Y., Gao, Z. S., Yang, Z. W., Shao, J. X., Zhao, X. Z., Dai, Y., & Van Ree, R. (2012). Anaphylaxis and generalized urticaria from eating Chinese bayberry fruit. J Zhejiang Univ Sci B, 13(10), 851-854. doi:10.1631/jzus.B1200150
348. Wang, H. Y., Zhang, C. Q., Sun, B. Q., Li, S. Y., Zheng, J. P., & Zhong, N. S. (2007). [A survey on the relationship between atopy and bronchial asthma among adolescents in the city of Guangzhou, China]. Zhonghua Jie He He Hu Xi Za Zhi, 30(7), 504-508.
349. Wang, J., Shao, H., Ye, L., Zhang, Y., & Qin, B. (2023). [Upregulation of IL-18 expression in blood CD4(+) Th2 cells of patients with allergic rhinitis]. Xi Bao Yu Fen Zi Mian Yi Xue Za Zhi, 39(12), 1100-1107.
350. Wang, J., Wu, Y., Li, J., Huang, X., & Zhu, R. (2017). Eight Aeroallergen Skin Extracts May Be the Optimal Panel for Allergic Rhinitis Patients in Central China. Int Arch Allergy Immunol, 173(4), 193-198. doi:10.1159/000479429
351. Wang, J. D., Lee, F. Y., & Chang, T. K. (2010). Sinus mass with nasal obstruction mimicking allergic rhinitis in a child. J Pediatr Hematol Oncol, 32(7), 575-576. doi:10.1097/MPH.0b013e3181db05c8
352. Wang, L., Zhang, X., Zhang, J., Fan, W., Lu, M., & Hu, J. (2018). Proteomic Analysis and Identification of Possible Allergenic Proteins in Mature Pollen of Populus tomentosa. Int J Mol Sci, 19(1). doi:10.3390/ijms19010250
353. Wang, M., She, W., Yang, J., Wang, X., Zhang, Y., Zhang, X., & Zhang, L. (2021). Gene Expression Analysis by Real-Time PCR in Nasal Brushings of Adult Patients with Allergic Rhinitis, Suspected Allergic Rhinitis, and Nonallergic Rhinitis. Int Arch Allergy Immunol, 182(4), 301-310. doi:10.1159/000510942
354. Wang, M., Xing, Z., Feng, W., He, X., & Ma, Y. (2006). [The clinical study about the serum total and specific IgE levels in Artemisia pollen-induced allergic rhinitis patients]. Lin Chuang Er Bi Yan Hou Ke Za Zhi, 20(20), 925-927.
355. Wang, M., Xing, Z. M., Yu, D. L., Yan, Z., & Yu, L. S. (2004). Association between HLA class II locus and the susceptibility to Artemisia pollen-induced allergic rhinitis in Chinese population. Otolaryngol Head Neck Surg, 130(2), 192-196. doi:10.1016/j.otohns.2003.08.012
356. Wang, M., Zuo, L. M., Niu, W. Q., Wang, C. L., Xu, R. M., Song, L. X., . . . Li, S. R. (2005). [Survey on prevalence of 1 526 children with sleep disturbances in age of 2 to 12 years old in Chengdu]. Zhonghua Yu Fang Yi Xue Za Zhi, 39(6), 400-402.
357. Wang, Q. P., Wu, K. M., Li, Z. Q., Xue, F., Chen, W., Ji, H., & Wang, B. L. (2012). Association between maternal allergic rhinitis and asthma on the prevalence of atopic disease in offspring. Int Arch Allergy Immunol, 157(4), 379-386. doi:10.1159/000328789
358. Wang, S. Y., Spongberg, S. A., Rubenstein, J. S., & Rubenstein, H. S. (1985). Ragweed in China. Nature, 316(6027), 386. doi:10.1038/316386b0
359. Wang, S. Z., Ma, F. M., & Zhao, J. D. (2013). Expressions of nuclear factor-kappa B p50 and p65 and their significance in the up-regulation of intercellular cell adhesion molecule-1 mRNA in the nasal mucosa of allergic rhinitis patients. Eur Arch Otorhinolaryngol, 270(4), 1329-1334. doi:10.1007/s00405-012-2136-y
360. Wang, T. Q., You, M. Y., Lu, F., Hu, Y. H., Sun, J. F., Wang, M. M., . . . Yin, D. P. (2023). [Analysis of big data characteristics of allergic rhinitis patients in Beijing City from 2016 to 2021]. Zhonghua Yu Fang Yi Xue Za Zhi, 57(9), 1380-1384. doi:10.3760/cma.j.cn112150-20220928-00936
361. Wang, W., Chen, H., Gao, N., Yu, S., Liao, J., Wang, S., . . . Liu, Z. (2022). Effect of acupuncture at the sphenopalatine ganglion for the treatment of moderate to severe seasonal allergic rhinitis: Study protocol for a three-armed randomized controlled trial. Front Med (Lausanne), 9, 904864. doi:10.3389/fmed.2022.904864
362. Wang, W., Huang, X., Chen, Z., Zheng, R., Chen, Y., Zhang, G., & Yang, Q. (2016). Prevalence and trends of sensitisation to aeroallergens in patients with allergic rhinitis in Guangzhou, China: a 10-year retrospective study. BMJ Open, 6(5), e011085. doi:10.1136/bmjopen-2016-011085
363. Wang, W., Wang, J., Song, G., Xie, H., Lin, X., Chai, R., . . . Zhong, N. (2022). Environmental and sensitization variations among asthma and/or rhinitis patients between 2008 and 2018 in China. Clin Transl Allergy, 12(2), e12116. doi:10.1002/clt2.12116
364. Wang, W., Wei, C., Cheng, Z., & Yang, J. (2020). Aberrant Th2 Immune Responses Are Associated With a Reduced Frequency of IL-35-Induced Regulatory T Cells After Allergen Exposure in Patients With Allergic Asthma. Allergy Asthma Immunol Res, 12(6), 1029-1045. doi:10.4168/aair.2020.12.6.1029
365. Wang, W., Yin, J., Wang, X., Ma, T., Lan, T., Song, Q., & Guo, Y. (2020). Relationship between serum inhibitory activity for IgE and efficacy of Artemisia pollen subcutaneous immunotherapy for allergic rhinitis: a preliminary self-controlled study. Allergy Asthma Clin Immunol, 16, 18. doi:10.1186/s13223-020-0416-4
366. Wang, W., Zhang, H., Zheng, W., & He, S. (2017). [Up-regulated expression of NK1R in eosinophil-enriched blood cells from patients with chronic spontaneous urticaria]. Xi Bao Yu Fen Zi Mian Yi Xue Za Zhi, 33(8), 1108-1112.
367. Wang, X., Chen, L., Ding, J., Wang, H., & Wang, X. (2023). Profiles of Birch Allergen Component Sensitization and Its Association with Pollen Food Allergy Syndrome in Northern China. J Asthma Allergy, 16, 1241-1250. doi:10.2147/jaa.S427764
368. Wang, X., Guo, M., Wang, H., & Wang, X. (2020). [Pollen allergen sensitization feature of seasonal allergic rhinitis in children and adolescents in northern China]. Lin Chuang Er Bi Yan Hou Tou Jing Wai Ke Za Zhi, 34(11), 1005-1010. doi:10.13201/j.issn.2096-7993.2020.11.011
369. Wang, X., Wang, M., Wang, J., Tian, H., & Zhong, C. (2020). [Research on the correlation between otorhinolaryngologic diseases and environmental meteorological factors in children]. Lin Chuang Er Bi Yan Hou Tou Jing Wai Ke Za Zhi, 34(6), 546-553. doi:10.13201/j.issn.2096-7993.2020.06.016
370. Wang, X., Zhuang, Y., Chen, Y., Wang, H., & Wang, X. (2021). Prevalence of adult eczema, hay fever, and asthma, and associated risk factors: a population-based study in the northern Grassland of China. Allergy Asthma Clin Immunol, 17(1), 27. doi:10.1186/s13223-021-00532-7
371. Wang, X. Y., Lan, T. F., Kong, R., Chen, Y. L., & Wang, X. Y. (2018). [The sensitization feature of artemisia allergy and the diagnosis value of skin prick test]. Lin Chuang Er Bi Yan Hou Tou Jing Wai Ke Za Zhi, 32(24), 1860-1864. doi:10.13201/j.issn.1001-1781.2018.24.004
372. Wang, X. Y., Lim-Jurado, M., Prepageran, N., Tantilipikorn, P., & Wang de, Y. (2016). Treatment of allergic rhinitis and urticaria: a review of the newest antihistamine drug bilastine. Ther Clin Risk Manag, 12, 585-597. doi:10.2147/tcrm.S105189
373. Wang, X. Y., Ma, T. T., Wang, X. Y., Zhuang, Y., Wang, X. D., Ning, H. Y., . . . Wang, D. Y. (2018). Prevalence of pollen-induced allergic rhinitis with high pollen exposure in grasslands of northern China. Allergy, 73(6), 1232-1243. doi:10.1111/all.13388
374. Wang, X. Y., Ning, H. Y., Chu, S. M., Jiang, L., Pang, Y. J., Jin, W. T., . . . Wang, X. Y. (2017). [An analysis of spectrum of allergen sensitization among patients with tree pollen allergy in Beijing]. Lin Chuang Er Bi Yan Hou Tou Jing Wai Ke Za Zhi, 31(23), 1819-1823. doi:10.13201/j.issn.1001-1781.2017.23.010
375. Wang, X. Y., Ning, H. Y., Li, X. R., & Wang, X. Y. (2018). [The sensitization feature of Cypress pollen in urban area of Beijing]. Zhonghua Er Bi Yan Hou Tou Jing Wai Ke Za Zhi, 53(10), 740-744. doi:10.3760/cma.j.issn.1673-0860.2018.10.005
376. Wang, X. Y., Tian, Z. M., Ning, H. Y., & Wang, X. Y. (2017). The ambient pollen distribution in Beijing urban area and its relationship with consumption of outpatient anti-allergic prescriptions. Eur Rev Med Pharmacol Sci, 21(3 Suppl), 108-115.
377. Wang, X. Y., Tian, Z. M., Ning, H. Y., & Wang, X. Y. (2017). [Association between airborne pollen distribution and allergic diseases in Beijing urban area]. Lin Chuang Er Bi Yan Hou Tou Jing Wai Ke Za Zhi, 31(10), 757-761. doi:10.13201/j.issn.1001-1781.2017.10.005
378. Wang, Y., Fang, H., Liu, X., Wang, J., Wang, Z., & Yang, J. (2021). [Analysis of allergen distribution and clinical characteristics of autumn allergic rhinitis in Changchun and surrounding areas]. Lin Chuang Er Bi Yan Hou Tou Jing Wai Ke Za Zhi, 35(12), 1124-1129. doi:10.13201/j.issn.2096-7993.2021.12.014
379. Wang, Y., Gao, Z., Lv, H., & Xu, Y. (2022). Online public attention toward allergic rhinitis in Wuhan, China: Infodemiology study using Baidu index and meteorological data. Front Public Health, 10, 971525. doi:10.3389/fpubh.2022.971525
380. Wang, Y., Li, J., Wang, F., Cui, Y., Song, L., Ruan, B., & Yu, Y. (2023). Analysis of ceRNA Regulatory Mechanism of Rape Pollen Allergy Based on Whole-Transcriptome Sequencing of Peripheral Blood Mononuclear Cells. J Asthma Allergy, 16, 775-788. doi:10.2147/jaa.S416772
381. Wang, Y., Li, Y., Zhao, C. Q., Han, Z. Q., & Zhu, M. L. (2019). [A Meta-analysis of sublingual immunotherapy in the treatment of seasonal allergic rhinitis]. Lin Chuang Er Bi Yan Hou Tou Jing Wai Ke Za Zhi, 33(1), 17-22. doi:10.13201/j.issn.1001-1781.2019.01.005
382. Wang, Y., Liu, Z. L., Sun, J., Ilham, I., Hu, H. F., & Zhang, H. (2011). [Relationship between polymorphism sites of Tim-3 and allergic rhinitis in a population of Uigurs and Hans from Xinjiang Uigur Autonomous Region of China]. Zhonghua Er Bi Yan Hou Tou Jing Wai Ke Za Zhi, 46(9), 712-717.
383. Wang, Y., Tan, L. X., Xu, Z. Q., Jiao, Y. X., Zhu, D. X., Yang, Y. S., . . . Tian, M. (2023). Identification and characterization of natural PR-1 protein as major allergen from Humulus japonicus pollen. Mol Immunol, 153, 170-180. doi:10.1016/j.molimm.2022.11.023
384. Wang, Y., Zhou, J., & Liu, B. (2007). Decreased suppression of immune stimulatory CpG motifs in plant DNA. Immunol Lett, 114(2), 73-80. doi:10.1016/j.imlet.2007.08.002
385. Wang, Z. (2006). [The effect of TNF-alpha & IL-8 in the pathogenesis of allergic rhinitis]. Lin Chuang Er Bi Yan Hou Ke Za Zhi, 20(23), 1060-1061.
386. Wang, Z. H. (1989). [A study of relationship between allergenic pollens and pollinosis in Dongying area]. Zhonghua Yu Fang Yi Xue Za Zhi, 23(6), 355-357.
387. Wang, Z. H., Lin, W. S., Li, S. Y., Zhao, S. C., Wang, L., Yang, Z. G., . . . Yu, J. Z. (2011). [Research on prevalence and related factors in allergic rhinitis]. Zhonghua Er Bi Yan Hou Tou Jing Wai Ke Za Zhi, 46(3), 225-231.
388. Wen, C. M., Ye, S. T., Zhou, L. X., & Yu, Y. (1990). Silk-induced asthma in children: a report of 64 cases. Ann Allergy, 65(5), 375-378.
389. Wen, L., Wen, Z., & Yang, N. (2014). [Risk factors and management of systemic reactions induced by subcutaneous immunotherapy]. Zhonghua Yi Xue Za Zhi, 94(38), 3001-3004.
390. Wen, T. H. (2009). Dermatophagoides farinae mite allergen and specific immunotherapy in Shanghai. Zhongguo Ji Sheng Chong Xue Yu Ji Sheng Chong Bing Za Zhi, 27(5), 419-425.
391. Wen, W. D., Yuan, F., Wang, J. L., & Hou, Y. P. (2007). Botulinum toxin therapy in the ovalbumin-sensitized rat. Neuroimmunomodulation, 14(2), 78-83. doi:10.1159/000107422
392. Wen, Z., & Ye, S. (2002). [A report of 50 patients with artemisia pollenosis and plant food allergy]. Zhonghua Yi Xue Za Zhi, 82(9), 626-629.
393. Wu, L., Hou, X., Luo, W., Hu, H., Zheng, X., Chen, Y., . . . Sun, B. (2022). Three patterns of sensitization to mugwort, timothy, birch and their major allergen components revealed by Latent class analysis. Mol Immunol, 145, 59-66. doi:10.1016/j.molimm.2022.03.009
394. Wu, L., Luo, W., Hu, H., Zheng, X., Cheng, Z. J., Huang, D., . . . Sun, B. (2021). A Multicenter Study Assessing Risk Factors and Aeroallergens Sensitization Characteristics in Children with Self-Reported Allergic Rhinitis in China. J Asthma Allergy, 14, 1453-1462. doi:10.2147/jaa.S342495
395. Wu, L., Zhang, T., Luo, W., Zheng, X., Zhang, H., Ren, H., . . . Sun, B. (2023). Rhinitis symptom in patients with self-reported allergic rhinitis is influenced by sensitization pattern: A cross-sectional study of China. Int Forum Allergy Rhinol, 13(6), 1007-1016. doi:10.1002/alr.23098
396. Xi, G. P., Zhang, Q., & Yin, J. (2021). Establishment and characterization of murine models of asthma and subcutaneous immunotherapy for Humulus pollen allergy. Immun Inflamm Dis, 9(2), 443-455. doi:10.1002/iid3.405
397. Xi, L., Wang, G., Shi, B., Li, Y., & Zhang, L. (2020). The work behaviors of patients with allergic rhinitis (AR) during the autumn pollen season. Ann Palliat Med, 9(5), 2776-2785. doi:10.21037/apm-20-267
398. Xi, L., Zhang, Y., Han, D., & Zhang, L. (2012). Effect of asthma, aeroallergen category, and gender on the psychological status of patients with allergic rhinitis. J Investig Allergol Clin Immunol, 22(4), 264-269.
399. Xiao, B., Deng, Y., Tao, Z., & Kong, Y. (2008). [Analysis about the results of allergy screen in 134 cases of allergic rhinitis]. Lin Chuang Er Bi Yan Hou Tou Jing Wai Ke Za Zhi, 22(5), 214-216.
400. Xiao, L., Li, B., Du, Y. H., Xiong, J., & Gao, X. (2009). [Systematic evaluation of the randomized controlled trials about acupuncture and moxibustion treatment of allergic rhinitis]. Zhongguo Zhen Jiu, 29(6), 512-516.
401. Xiao, X., Fu, A., Xie, X., Kang, M., Hu, D., Yang, P., & Liu, Z. (2013). An investigation of airborne allergenic pollen at different heights. Int Arch Allergy Immunol, 160(2), 143-151. doi:10.1159/000339673
402. Xiao, X., Zeng, X., Zhang, X., Ma, L., Liu, X., Yu, H., . . . Liu, Z. (2013). Effects of Caryota mitis profilin-loaded PLGA nanoparticles in a murine model of allergic asthma. Int J Nanomedicine, 8, 4553-4562. doi:10.2147/ijn.S51633
403. Xie, Q. M., Deng, J. F., Deng, Y. M., Shao, C. S., Zhang, H., & Ke, C. K. (2006). Effects of cryptoporus polysaccharide on rat allergic rhinitis associated with inhibiting eotaxin mRNA expression. J Ethnopharmacol, 107(3), 424-430. doi:10.1016/j.jep.2006.03.040
404. Xie, Z., Sun, H., Li, X., Sun, W., & Yin, J. (2021). Alteration of lung tissues proteins in birch pollen induced asthma mice before and after SCIT. PLoS ONE, 16(10), e0258051. doi:10.1371/journal.pone.0258051
405. Xie, Z., & Yin, J. (2019). Chinese Birch Pollen Allergy and Immunotherapy in Mice. Inflammation, 42(3), 961-972. doi:10.1007/s10753-019-00957-8
406. Xie, Z. J., Guan, K., & Yin, J. (2019). Advances in the clinical and mechanism research of pollen induced seasonal allergic asthma. Am J Clin Exp Immunol, 8(1), 1-8.
407. Xiong, L., Lin, J., Luo, Y., Chen, W., & Dai, J. (2020). The Efficacy and Safety of Epicutaneous Immunotherapy for Allergic Diseases: A Systematic Review and Meta-Analysis. Int Arch Allergy Immunol, 181(3), 170-182. doi:10.1159/000504366
408. Xiong, M., Fu, X., Deng, W., Lai, H., & Yang, C. (2014). Tap water nasal irrigation in adults with seasonal allergic rhinitis: a randomized double-blind study. Eur Arch Otorhinolaryngol, 271(6), 1549-1552. doi:10.1007/s00405-013-2741-4
409. Xu, D., Wang, Y., Chen, Z., Li, S., Cheng, Y., Zhang, L., & Zhao, L. (2016). Prevalence and risk factors for asthma among children aged 0-14 years in Hangzhou: a cross-sectional survey. Respir Res, 17(1), 122. doi:10.1186/s12931-016-0439-z
410. Xu, F., Zou, Z., Yan, S., Li, F., Kan, H., Norback, D., . . . Zhao, Z. (2011). Fractional exhaled nitric oxide in relation to asthma, allergic rhinitis, and atopic dermatitis in Chinese children. J Asthma, 48(10), 1001-1006. doi:10.3109/02770903.2011.627487
411. Xu, G., Cheng, L., Wen, W., Oh, Y., Mou, Z., Shi, J., . . . Li, H. (2007). Inverse association between T-cell immunoglobulin and mucin domain-1 and T-bet in a mouse model of allergic rhinitis. Laryngoscope, 117(6), 960-964. doi:10.1097/mlg.0b013e318041549c
412. Xu, G., Zhang, L., Wang, D. Y., Xu, R., Liu, Z., Han, D. M., . . . Li, H. B. (2010). Opposing roles of IL-17A and IL-25 in the regulation of TSLP production in human nasal epithelial cells. Allergy, 65(5), 581-589. doi:10.1111/j.1398-9995.2009.02252.x
413. Xu, L., Luo, W., Lu, Y., Huang, Z., Yu, X., Liao, C., . . . Sun, B. (2022). A comprehensive analysis of the components of common weed pollen and related allergens in patients with allergic diseases in southern China. Mol Immunol, 147, 180-186. doi:10.1016/j.molimm.2022.05.005
414. Xu, L. J., Zhang, S. Y., Yang, Q., Cheng, L., Yin, M., & Miyoshi, A. (2000). [A preliminary study on the incidence of cedar pollinosis in district of Wuhan]. Lin Chuang Er Bi Yan Hou Ke Za Zhi, 14(11), 505-506.
415. Xu, M. Y., Ye, Q. Y., Zhang, J. L., Huang, Z. C., Wang, Y., Liu, J., . . . Luo, W. T. (2023). [Study on sIgE distribution characteristics and the sensitization pattern of allergen in 1 161 patients with allergic diseases of respiratory tract in northwest China]. Zhonghua Yu Fang Yi Xue Za Zhi, 57(9), 1355-1363. doi:10.3760/cma.j.cn112150-20230507-00352
416. Xu, Q., Zhang, Y., & Zhang, L. (2020). Effect of perennial dust mites allergy on symptom severity of autumn allergic rhinitis in adults. Allergy Asthma Proc, 41(5), 363-371. doi:10.2500/aap.2020.41.200046
417. Xu, X., Qin, L., Ren, L., Wang, C., Zhang, Y., & Zhang, L. (2021). Comparative analysis of chronic rhinitis patient profiles during autumn pollen season between grassland and non-grassland cities in North China. Allergy Asthma Clin Immunol, 17(1), 106. doi:10.1186/s13223-021-00591-w
418. Xu, X. R., Guo, L. Y., Liu, H. F., Gong, W. X., Zhang, Y., & Ye, T. (2000). [Characteristics and effect of allergic rhinitis in aircrew on flight]. Space Med Med Eng (Beijing), 13(3), 207-209.
419. Xu, Y., Gepp, B., Lengger, N., Yin, J., & Breiteneder, H. (2023). Identification of probable pectinesterase as a major allergen of pollen of the Asian white birch (Betula platyphylla) in northern China. Asian Pac J Allergy Immunol. doi:10.12932/ap-100722-1409
420. Xu, Y., Guan, K., Sha, L., Zhang, J., Niu, Y., Yin, J., & Wang, L. (2021). Sensitization Profiles of Timothy Grass Pollen in Northern China. J Asthma Allergy, 14, 1431-1439. doi:10.2147/jaa.S334183
421. Xu, Y., Zhang, J., & Wang, J. (2014). The efficacy and safety of selective H1-antihistamine versus leukotriene receptor antagonist for seasonal allergic rhinitis: a meta-analysis. PLoS ONE, 9(11), e112815. doi:10.1371/journal.pone.0112815
422. Xu, Y. Y., Xue, T., Li, H. R., & Guan, K. (2021). Retrospective analysis of epidemic thunderstorm asthma in children in Yulin, northwest China. Pediatr Res, 89(4), 958-961. doi:10.1038/s41390-020-0980-9
423. Xu-De, Z., Bei-Bei, G., Xi-Juan, W., Hai-Bo, L., Li-Li, Z., & Feng-Xia, L. (2021). Serum IgE Predicts Difference of Population and Allergens in Allergic Diseases: Data from Weifang City, China. Mediators Inflamm, 2021, 6627087. doi:10.1155/2021/6627087
424. Xue, J., Zhao, C., & Liang, A. (2011). [Pathway analysis of the differential expression genes of oligonucleotide microarray of airway allergic diseases using GenMAPP]. Lin Chuang Er Bi Yan Hou Tou Jing Wai Ke Za Zhi, 25(8), 371-373.
425. Xue, J., Zhao, C., Xie, J., & Wei, R. (2010). [Expression and DNA binding activity of NF-kappaB p50 in nasal mucosal tissue of seasonal allergic rhinitis]. Lin Chuang Er Bi Yan Hou Tou Jing Wai Ke Za Zhi, 24(11), 498-501.
426. Xue, J. M., Zhao, C. Q., Zhao, H. L., Liang, A. H., & Xie, J. (2007). [Differential gene expression profile in seasonal allergic rhinitis with and without asthma]. Zhonghua Er Bi Yan Hou Tou Jing Wai Ke Za Zhi, 42(9), 654-659.
427. Yan, A., Luo, G., Zhou, Z., Hang, W., & Qin, D. (2018). Tear osteopontin level and its relationship with local Th1/Th2/Th17/Treg cytokines in children with allergic conjunctivitis. Allergol Immunopathol (Madr), 46(2), 144-148. doi:10.1016/j.aller.2017.05.005
428. Yan, T., Song, X., Ding, X., Liu, X., Lan, T., Chen, T., . . . Wang, M. (2023). Exposure to household dust, allergens, and endotoxin and allergy-related outcomes alternation in the general U.S. population. Environ Res, 226, 115713. doi:10.1016/j.envres.2023.115713
429. Yan, Z., Zhang, R., Yu, S., & Wu, G. (2009). [Study on the expression of Eotaxin and the role of histamine in allergic rhinitis]. Lin Chuang Er Bi Yan Hou Tou Jing Wai Ke Za Zhi, 23(23), 1086-1088.
430. Yang, J., & Lei, S. (2023). Efficacy and safety of sublingual versus subcutaneous immunotherapy in children with allergic rhinitis: a systematic review and meta-analysis. Front Immunol, 14, 1274241. doi:10.3389/fimmu.2023.1274241
431. Yang, J., Shen, Z., Liu, L., Kang, W., Shao, Y., Zhang, P., & Quan, F. (2022). Clinical Efficacy and Safety of Artesimia annua-Sublingual Immunotherapy in Seasonal Allergic Rhinitis Patients Based on Different Intervention Time. Int Arch Allergy Immunol, 183(8), 852-859. doi:10.1159/000524108
432. Yang, J., Wang, W., Shen, Z., Kang, W., Zhang, P., Chen, X., . . . Quan, F. (2023). Efficacy and safety of Artemisia annua sublingual immunotherapy in patients with seasonal allergic rhinoconjunctivitis over two pollen seasons. Eur Arch Otorhinolaryngol, 280(11), 4939-4947. doi:10.1007/s00405-023-08078-7
433. Yang, J., Zhang, L., Zhao, Z., & Liao, S. (2018). Sublingual immunotherapy for pediatric allergic conjunctivitis: a meta-analysis of randomized controlled trials. Int Forum Allergy Rhinol, 8(11), 1253-1259. doi:10.1002/alr.22149
434. Yang, L., Li, W., Qi, S., Jiang, Q., Huang, N., Yang, Y., . . . Zhu, R. (2023). A Survey of Airborne Fungi and Their Sensitization Profile in Wuhan, China. Int Arch Allergy Immunol, 184(11), 1153-1164. doi:10.1159/000531245
435. Yang, L. F., Cai, L. M., Li, M., Liu, J. T., Wang, Z. N., Wang, W. H., . . . Chen, Z. G. (2018). A 10-year retrospective study of alterative aeroallergens sensitization spectrum in urban children with allergic rhinitis. Ther Clin Risk Manag, 14, 409-416. doi:10.2147/tcrm.S154692
436. Yang, P. C., Liu, T., Zhang, T. Y., & Fan, D. S. (1998). Effect of substance P on the short-circuit current of rat nasal mucosal epithelium. Ann Otol Rhinol Laryngol, 107(8), 675-679. doi:10.1177/000348949810700809
437. Yang, X., Zhang, J., Sima, Y., Zhao, J., Wang, X., & Zhang, L. (2023). A multicenter time series study on pollen exposure and COVID-19 infection in five cities in China. Allergy, 78(6), 1711-1713. doi:10.1111/all.15752
438. Yang, Y., & Gu, M. (2019). Association of body mass index and season with histamine skin reactivity in Chinese children with allergic rhinitis. Pediatr Neonatol, 60(2), 172-177. doi:10.1016/j.pedneo.2018.05.011
439. Yang, Y., Zhao, Y., Wang, C. S., Wang, X. D., & Zhang, L. (2011). [Prevalence of sensitization to aeroallergens in 10 030 patients with allergic rhinitis]. Zhonghua Er Bi Yan Hou Tou Jing Wai Ke Za Zhi, 46(11), 914-920.
440. Yang, Y., Zhou, W., & Chen, A. (2016). Efficacy of sublingual immunotherapy for cedar pollinosis: A systematic review and meta-analysis. Ann Allergy Asthma Immunol, 117(4), 348-353. doi:10.1016/j.anai.2016.07.024
441. Yang, Y. P., Maimaiti, Y., Wang, Y., Wang, L. L., Tan, G. P., & Zhang, H. (2017). [Changing analysis of allergic rhinitis inhalant allergen spectrum in Xinjiang region]. Zhonghua Er Bi Yan Hou Tou Jing Wai Ke Za Zhi, 52(5), 355-360. doi:10.3760/cma.j.issn.1673-0860.2017.05.007
442. Yang, Y. S., Xu, Z. Q., Zhu, W., Zhu, D. X., Jiao, Y. X., Zhang, L. S., . . . Sun, J. L. (2022). Molecular and immunochemical characterization of profilin as major allergen from Platanus acerifolia pollen. Int Immunopharmacol, 106, 108601. doi:10.1016/j.intimp.2022.108601
443. Yao, J., Xu, M., & Zhang, Y. (2009). [Experimental research on the relationship between GATA-3 and ICAM-1 in patients with allergic rhinitis]. Lin Chuang Er Bi Yan Hou Tou Jing Wai Ke Za Zhi, 23(2), 70-72.
444. Yao, L., & Zhang, H. (2009). [Concentration of airborne pollen in Beijing city with burkard sampler]. Lin Chuang Er Bi Yan Hou Tou Jing Wai Ke Za Zhi, 23(20), 913-916.
445. Yao, T. C., Chang, S. W., Hua, M. C., Liao, S. L., Tsai, M. H., Lai, S. H., . . . Huang, J. L. (2016). Tobacco smoke exposure and multiplexed immunoglobulin E sensitization in children: a population-based study. Allergy, 71(1), 90-98. doi:10.1111/all.12775
446. Yin, J., He, H. J., Wang, R. Q., Xu, T., Yue, F. M., Wang, L. L., . . . Ye, S. T. (2006). [Value of intradermal skin test and serum sIgE detection in diagnosing artemisia pollinosis]. Zhonghua Yi Xue Za Zhi, 86(25), 1759-1763.
447. Yin, J., Wang, R. Q., He, H. J., Xu, T., Yue, F. M., Wang, L. L., . . . Ye, S. T. (2006). [Values of intradermal skin test and serum sIgE detection in diagnosing Humulus scandens pollinosis]. Zhonghua Yi Xue Za Zhi, 86(27), 1906-1911.
448. Yin, J., Ye, S., Zou, M., He, H., & Gu, R. (1998). [An ELISA assay for humulus scandens specific IgE antibodies]. Zhongguo Yi Xue Ke Xue Yuan Xue Bao, 20(2), 148-153.
449. Yin, J., Yue, F. M., Wang, L. L., He, H. J., Xu, T., Li, H., . . . Ye, S. T. (2006). [Natural course from rhinitis to asthma in the patients with autumnal pollinosis: a clinical study of 1096 patients]. Zhonghua Yi Xue Za Zhi, 86(23), 1628-1632.
450. Yin, J., Yue, F. M., Wang, L. L., He, H. J., Xu, T., Zhang, H. Y., . . . Ye, S. T. (2005). [The clinical study of the relationship between allergic rhinitis and allergic asthma in the patients with autumnal pollinosis]. Zhonghua Yi Xue Za Zhi, 85(24), 1683-1687.
451. Yin, M., Cheng, L., Miyoshi, A., Peng, X., Ruan, Z., Shi, H., . . . Imano, A. (1999). [Epidemiological survey on orchard pollinosis]. Lin Chuang Er Bi Yan Hou Ke Za Zhi, 13(11), 513-514.
452. Yin, Z., Ouyang, Y., Dang, B., & Zhang, L. (2023). Pollen grading prediction scale for patients with Artemisia pollen allergy in China: A 3-day moving predictive model. Clin Transl Allergy, 13(7), e12280. doi:10.1002/clt2.12280
453. Yin, Z. X., Zhu, Y., Zhai, X., Zhang, J. L., & Liu, G. (2017). [Clinical symptoms and immunology inspection characteristics of nasal cavity local allergy]. Lin Chuang Er Bi Yan Hou Tou Jing Wai Ke Za Zhi, 31(15), 1146-1151. doi:10.13201/j.issn.1001-1781.2017.15.002
454. Ying, X., Zhang, R., Yu, S., Wu, J., & Wang, H. (2012). Association of interleukin-13 SNP rs1800925 with allergic rhinitis risk: a meta-analysis based on 1,411 cases and 3169 controls. Gene, 506(1), 179-183. doi:10.1016/j.gene.2012.06.066
455. You, X., Liu, Z., Xu, K., Gao, Q., & Cui, Y. (2008). [The investigation of efficacy and safety of Aerius to seasonal allergic rhinitis]. Lin Chuang Er Bi Yan Hou Tou Jing Wai Ke Za Zhi, 22(8), 382-384.
456. Yu, D. D., Liu, Y., Zheng, Z. Y., Zhang, J., Gao, L., Zhang, M., . . . Feng, Y. (2023). [Efficacy observation and compliance analysis of pollen allergen drops in seasonal allergic rhinitis]. Zhonghua Yu Fang Yi Xue Za Zhi, 57(12), 1996-2001. doi:10.3760/cma.j.cn112150-20230711-00002
457. Yu, R. L., Pan, C., Ma, T. T., Wang, X. Y., Shi, H. Y., Zhuang, Y., . . . Wang, X. Y. (2022). Prediction of clinical efficacy of subcutaneous immunotherapy for Artemisia sieversiana pollen allergic rhinitis by serum metabolomics. J Formos Med Assoc, 121(12), 2465-2480. doi:10.1016/j.jfma.2022.05.006
458. Yu, R. L., Wang, J., Wang, X. S., Wang, H. T., & Wang, X. Y. (2023). Management of allergic rhinitis improves clinical outcomes of difficult-to-treat tic disorders or attention-deficit/hyperactivity disorders. Allergol Select, 7, 191-197. doi:10.5414/alx400588e
459. Yu, W. T., Zhou, Y., Tan, H. F., Shi, Q. M., Wang, Y. J., Zhu, Y., . . . Chen, J. J. (2019). [The diagnostic value of eosinophilic and eosinophilic cationic proteins in blood and nasal secretions for allergic rhinitis]. Lin Chuang Er Bi Yan Hou Tou Jing Wai Ke Za Zhi, 33(11), 1027-1030. doi:10.13201/j.issn.1001-1781.2019.11.005
460. Yuan, L., Lu, S., Zhang, H., Wang, J., Hu, Y., Wang, L., & He, S. (2017). [Elevated IL-18, IL-18 binding protein and IL-18 receptor in eosinophil-enriched blood cells in patients with asthma]. Xi Bao Yu Fen Zi Mian Yi Xue Za Zhi, 33(4), 526-530.
461. Yuhao, G., Liangran, H., Lei, Z., Wei, Y., Lixia, H., Wei, L., & Yuyan, F. (2023). Early intervention improves clinical responses to seasonal allergic rhinitis by stimulation in sphenopalatine ganglion (Xinwu acupoint). J Tradit Chin Med, 43(4), 787-794. doi:10.19852/j.cnki.jtcm.20230524.001
462. Zeng, G., Hu, H., Zheng, P., Wu, G., Wei, N., Liang, X., . . . Zhang, X. (2018). The practical benefit of Phadiatop test as the first-line in vitro allergen-specific immunoglobulin E (sIgE) screening of aeroallergens among Chinese asthmatics: a validation study. Ann Transl Med, 6(8), 151. doi:10.21037/atm.2018.04.06
463. Zeng, G., Luo, W., Wu, Z., Li, L., Zheng, P., Huang, H., . . . Liu, Y. (2017). A cross-sectional observational study on allergen-specific IgE positivity in a southeast coastal versus a southwest inland region of China. Sci Rep, 7(1), 9593. doi:10.1038/s41598-017-10109-3
464. Zeng, M., Long, X. B., Cui, Y. H., & Liu, Z. (2011). Comparison of efficacy of mometasone furoate versus clarithromycin in the treatment of chronic rhinosinusitis without nasal polyps in Chinese adults. Am J Rhinol Allergy, 25(6), e203-207. doi:10.2500/ajra.2011.25.3728
465. Zeng, R., Liang, Z. T., Huang, S. Y., Zhang, J. T., Qiao, X. R., Yang, H., & Dong, L. (2022). [Clinical analysis of chronic eosinophilic pneumonia in 9 cases]. Zhonghua Jie He He Hu Xi Za Zhi, 45(4), 368-372. doi:10.3760/cma.j.cn112147-20211213-00881
466. Zhang, B., Li, P., & Fu, P. (2023). Association between Polyunsaturated Fatty Acid Intake and Eczema in Children and Adolescents. Int Arch Allergy Immunol, 184(7), 681-691. doi:10.1159/000529435
467. Zhang, F., Krafft, T., Ye, B., Zhang, F., Zhang, J., Luo, H., . . . Wang, W. (2013). The lag effects and seasonal differences of air pollutants on allergic rhinitis in Beijing. Sci Total Environ, 442, 172-176. doi:10.1016/j.scitotenv.2012.09.039
468. Zhang, F., Krafft, T., Zhang, D., Xu, J., & Wang, W. (2012). The association between daily outpatient visits for allergic rhinitis and pollen levels in Beijing. Sci Total Environ, 417-418, 39-44. doi:10.1016/j.scitotenv.2011.12.021
469. Zhang, H., Zhuang, L., Jin, R., Li, N., & Zhou, G. (2017). [Recombinant platanus pollen allergen 2 (rPla a2) up-regulates the expression of ORMDL3 mediated by GATA3 in NIH3T3 cells]. Xi Bao Yu Fen Zi Mian Yi Xue Za Zhi, 33(1), 27-32.
470. Zhang, J., Chen, H., Zhang, X., Chen, M., & Zhang, Y. M. (2011). [Study on factors related to allergic rhinitis natural course in children]. Zhonghua Er Bi Yan Hou Tou Jing Wai Ke Za Zhi, 46(1), 31-35.
471. Zhang, J., Luo, W., Li, G., Ren, H., Su, J., Sun, J., . . . Sun, B. (2023). Patterns of aeroallergen sensitization in asthma patients identified by latent class analysis: A cross-sectional study in China. Clin Transl Allergy, 13(7), e12271. doi:10.1002/clt2.12271
472. Zhang, J., Wu, L. S., Fan, W., Zhang, X. L., Jia, H. X., Li, Y., . . . Lu, M. Z. (2015). Proteomic analysis and candidate allergenic proteins in Populus deltoides CL. "2KEN8" mature pollen. Front Plant Sci, 6, 548. doi:10.3389/fpls.2015.00548
473. Zhang, L., Cheng, L., & Hong, J. (2013). The clinical use of cetirizine in the treatment of allergic rhinitis. Pharmacology, 92(1-2), 14-25. doi:10.1159/000351843
474. Zhang, L., Han, D., Huang, D., Wu, Y., Dong, Z., Xu, G., . . . Bachert, C. (2009). Prevalence of self-reported allergic rhinitis in eleven major cities in china. Int Arch Allergy Immunol, 149(1), 47-57. doi:10.1159/000176306
475. Zhang, L., Han, D. M., Huang, D., Wang, C. S., Wang, X. D., Zhou, B., & Zhao, Y. (2007). [Impacts of socioeconomic and environmental factors on self-reported prevalence of allergic rhinitis in eleven cities in China]. Zhonghua Er Bi Yan Hou Tou Jing Wai Ke Za Zhi, 42(6), 452-456.
476. Zhang, L., Wei, J. M., & Han, D. M. (2010). [Current state of diagnosis and treatment of allergic rhinitis in China]. Zhonghua Er Bi Yan Hou Tou Jing Wai Ke Za Zhi, 45(5), 420-423.
477. Zhang, L., Xu, G., Wang, X., Liu, S., Li, Y., Wang, S., . . . Han, D. (2009). Mometasone furoate nasal spray reduces symptoms and improves quality of life in Chinese patients with moderate to severe allergic rhinitis: a multicenter open-label study. Acta Otolaryngol, 129(12), 1463-1468. doi:10.3109/00016480902856570
478. Zhang, N., Wu, Y., Zhang, Q., Wei, Z., & Liu, Y. (2022). [Analysis of inhalation allergen of patients with allergic rhinitis in Shenzhen]. Lin Chuang Er Bi Yan Hou Tou Jing Wai Ke Za Zhi, 36(6), 467-472. doi:10.13201/j.issn.2096-7993.2022.06.012
479. Zhang, Q., Xi, G., & Yin, J. (2021). Artemisia sieversiana pollen allergy and immunotherapy in mice. Am J Transl Res, 13(12), 13654-13664.
480. Zhang, X., Chen, H., Liu, S., Wang, X., Guo, B., Liu, J., . . . Liu, F. (2023). Allergen Sensitization in Children in Weifang, China: Differences Between Monosensitization and Polysensitization. J Asthma Allergy, 16, 397-410. doi:10.2147/jaa.S405549
481. Zhang, X., Li, F., Zhang, L., Zhao, Z., & Norback, D. (2014). A longitudinal study of sick building syndrome (SBS) among pupils in relation to SO2, NO2, O3 and PM10 in schools in China. PLoS ONE, 9(11), e112933. doi:10.1371/journal.pone.0112933
482. Zhang, X., & McElwee, K. J. (2020). Allergy promotes alopecia areata in a subset of patients. Exp Dermatol, 29(3), 239-242. doi:10.1111/exd.14027
483. Zhang, X., Zhang, R., Zhang, Y., & Lu, T. (2023). Associations between attention-deficit/hyperactivity disorder and allergic diseases: a two-sample Mendelian randomization study. Front Psychiatry, 14, 1185088. doi:10.3389/fpsyt.2023.1185088
484. Zhang, Y., Hu, W., Chen, D., Ding, M., Wang, T., Wang, Y., . . . Li, C. (2022). An allergenic plant calmodulin from Artemisia pollen primes human DCs leads to Th2 polarization. Front Immunol, 13, 996427. doi:10.3389/fimmu.2022.996427
485. Zhang, Y., Lan, F., & Zhang, L. (2021). Advances and highlights in allergic rhinitis. Allergy, 76(11), 3383-3389. doi:10.1111/all.15044
486. Zhang, Y., Shan, C., Liu, W., Han, Y., Shi, G., Ma, Y., . . . Zhang, L. (2022). Intranasal budesonide for rhinitis during a high airborne pollution period: a randomized controlled trial. Allergy Asthma Clin Immunol, 18(1), 56. doi:10.1186/s13223-022-00686-y
487. Zhang, Y., Song, X., Zhao, Y., Zhang, L., & Bachert, C. (2012). Single nucleotide polymorphisms in thymic stromal lymphopoietin gene are not associated with allergic rhinitis susceptibility in Chinese subjects. BMC Med Genet, 13, 79. doi:10.1186/1471-2350-13-79
488. Zhang, Y., Wang, C. S., & Zhang, L. (2010). [Correlation between FOXP3 gene polymorphisms and allergic rhinitis]. Zhonghua Er Bi Yan Hou Tou Jing Wai Ke Za Zhi, 45(5), 397-400.
489. Zhang, Y., Xi, L., Gao, Y., Huang, Y., Cao, F., Xiong, W., . . . Zhang, L. (2022). Omalizumab is effective in the preseasonal treatment of seasonal allergic rhinitis. Clin Transl Allergy, 12(1), e12094. doi:10.1002/clt2.12094
490. Zhang, Y., Xi, L., Zhao, Y. M., Zhao, L. P., & Zhang, L. (2012). [Association between single-nucleotide polymorphisms in the IRAK-4 gene and allergic rhinitis]. Zhonghua Er Bi Yan Hou Tou Jing Wai Ke Za Zhi, 47(6), 476-480.
491. Zhang, Y. Q. (2009). Clinical experience in acupuncture treatment of allergic rhinitis. J Tradit Chin Med, 29(3), 186-189. doi:10.1016/s0254-6272(09)60062-5
492. Zhang, Z., Zhang, H., Yang, L., Chen, X., Norbäck, D., & Zhang, X. (2022). Associations between outdoor air pollution, ambient temperature and fraction of exhaled nitric oxide (FeNO) in university students in northern China - A panel study. Environ Res, 212(Pt C), 113379. doi:10.1016/j.envres.2022.113379
493. Zhao, C., Yue, F., & Yao, S. (1990). Treatment of allergic rhinitis by medicinal injection at fengmen acupoint. J Tradit Chin Med, 10(4), 264-266.
494. Zhao, L., Chen, J., Wang, Y., Guo, Y., Feng, Y., Wang, X., . . . Gao, Z. (2023). Association in molecular profiles of IgE sensitization to mugwort pollen allergens in Chinese parents and their offspring. Pediatr Allergy Immunol, 34(8), e14005. doi:10.1111/pai.14005
495. Zhao, L., Fu, W., Gao, B., Liu, Y., Wu, S., Chen, Z., . . . van Ree, R. (2020). Variation in IgE binding potencies of seven Artemisia species depending on content of major allergens. Clin Transl Allergy, 10(1), 50. doi:10.1186/s13601-020-00354-7
496. Zhao, Y., Sun, Z., Xiang, L., An, X., Hou, X., Shang, J., . . . Ye, C. (2023). Effects of pollen concentration on allergic rhinitis in children: A retrospective study from Beijing, a Chinese megacity. Environ Res, 229, 115903. doi:10.1016/j.envres.2023.115903
497. Zhao, Y., Zhao, Y., Zhang, Y., & Zhang, L. (2019). HLA-II genes are associated with outcomes of specific immunotherapy for allergic rhinitis. Int Forum Allergy Rhinol, 9(11), 1311-1317. doi:10.1002/alr.22384
498. Zhao, Z., Huang, C., Zhang, X., Xu, F., Kan, H., Song, W., . . . Norback, D. (2013). Fractional exhaled nitric oxide in Chinese children with asthma and allergies--a two-city study. Respir Med, 107(2), 161-171. doi:10.1016/j.rmed.2012.11.001
499. Zhao, Z. H., Elfman, L., Wang, Z. H., Zhang, Z., & Norbäck, D. (2006). A comparative study of asthma, pollen, cat and dog allergy among pupils and allergen levels in schools in Taiyuan city, China, and Uppsala, Sweden. Indoor Air, 16(6), 404-413. doi:10.1111/j.1600-0668.2006.00433.x
500. Zheng, H. S., Zhu, W. J., Liu, C. H., Li, Y. X., Song, X., Han, T. T., . . . Sha, L. (2023). [Clinical characteristics of children with IgE-mediated cow's milk protein allergy]. Zhonghua Yu Fang Yi Xue Za Zhi, 57(8), 1271-1279. doi:10.3760/cma.j.cn112150-20230514-00370
501. Zheng, Y. W., Li, J., Lai, X. X., Zhao, D. Y., Liu, X. F., Lin, X. P., . . . Spangfort, M. D. (2011). Allergen micro-array detection of specific IgE-reactivity in Chinese allergy patients. Chin Med J (Engl), 124(24), 4350-4354.
502. Zhong, Q., Zhan, M., Wang, L., Chen, D., Zhao, N., Wang, J., . . . Zhang, H. (2021). Upregulation of the expression of Toll-like receptor 9 in basophils in patients with allergic rhinitis: An enhanced expression by allergens. Scand J Immunol, 93(3), e13003. doi:10.1111/sji.13003
503. Zhong, Z., Wang, F., Wang, T., Li, L., & Tan, G. (2011). [Aeroallergen spectrum of patients with child allergic rhinitis in Changsha area of China]. Lin Chuang Er Bi Yan Hou Tou Jing Wai Ke Za Zhi, 25(17), 774-776.
504. Zhou, B., Cheng, L., Pan, J., Wang, H., Jin, Y., Zhao, C., . . . Nguyen, D. T. (2023). A Clinical Study to Assess the Efficacy and Safety of MP-AzeFlu Nasal Spray in Comparison to Commercially Available Azelastine Hydrochloride and Fluticasone Propionate Nasal Sprays in Chinese Volunteers with Allergic Rhinitis. Pulm Ther, 9(3), 411-427. doi:10.1007/s41030-023-00238-8
505. Zhou, M., Guan, W. J., Zuo, K. J., Xu, Z. F., Shi, J. B., & Lai, Y. Y. (2019). Powder Cellulose in Allergic Rhinitis Management: Relevance of in vitro Findings to Real-Life Safety - Author's Reply. Int Arch Allergy Immunol, 179(1), 19-20. doi:10.1159/000497116
506. Zhou, S., Wang, X., Lu, S., Yao, C., Zhang, L., Rao, L., . . . Wang, Q. (2021). Characterization of allergenicity of Platanus pollen allergen a 3 (Pla a 3) after exposure to NO(2) and O(3). Environ Pollut, 278, 116913. doi:10.1016/j.envpol.2021.116913
507. Zhou, S., Zhou, C., Wang, X., Luo, P., Lin, A., Cui, Y., & Qiu, Q. (2023). Profiles of immune infiltration in seasonal allergic rhinitis and related genes and pathways. Int Immunopharmacol, 120, 110174. doi:10.1016/j.intimp.2023.110174
508. Zhou, X., Jin, Y., & He, X. (2004). [Correlations between skin allergy caused by common environmental factors and asthma]. Wei Sheng Yan Jiu, 33(2), 236-238.
509. Zhou, Y., Dai, J., Liu, H., & Liu, X. (2022). Tourist risk assessment of pollen allergy in tourism attractions: A case study in the Summer Palace, Beijing, China. Front Public Health, 10, 1030066. doi:10.3389/fpubh.2022.1030066
510. Zhu, B. J., Qian, Z. Q., Yang, H. R., & Li, R. X. (2021). Tripterine: A Potential Anti-Allergic Compound. Curr Pharm Biotechnol, 22(1), 159-167. doi:10.2174/1389201021666200327163322
511. Zhu, C., Farah, J., Choël, M., Gosselin, S., Baroudi, M., Petitprez, D., & Visez, N. (2018). Uptake of ozone and modification of lipids in Betula Pendula pollen. Environ Pollut, 242(Pt A), 880-886. doi:10.1016/j.envpol.2018.07.025
512. Zhu, D. D., Zhu, X. W., Jiang, X. D., & Dong, Z. (2009). Thymic stromal lymphopoietin expression is increased in nasal epithelial cells of patients with mugwort pollen sensitive-seasonal allergic rhinitis. Chin Med J (Engl), 122(19), 2303-2307.
513. Zhu, H. M., Zhang, Y. J., Zhang, W. T., Su, K. M., & Shi, H. B. (2011). [Analysis of allergens characteristic in 4637 patients with allergic rhinitis]. Zhonghua Er Bi Yan Hou Tou Jing Wai Ke Za Zhi, 46(11), 933-936.
514. Zhu, J., Song, J., Liu, Z., Han, J., Luo, H., Liu, Y., . . . Zhong, W. (2016). Association between allergic conditions and risk of prostate cancer: A Prisma-Compliant Systematic Review and Meta-Analysis. Sci Rep, 6, 35682. doi:10.1038/srep35682
515. Zhu, K., Hou, X. L., Huang, H. J., Wang, Y. R., Ren, Y. X., Ni, X., & Xiang, L. (2017). [Distribution characteristics of serum specific IgE for inhaled allergens in children with different airway allergic diseases]. Zhongguo Dang Dai Er Ke Za Zhi, 19(11), 1185-1190. doi:10.7499/j.issn.1008-8830.2017.11.012
516. Zhu, L. P., Tao, Q. L., Lu, M. P., Wang, Y. L., Chen, R. X., Lu, Y., . . . Cheng, L. (2012). [Comparison of clinical characteristics between patients with non-allergic and allergic rhinitis]. Zhonghua Er Bi Yan Hou Tou Jing Wai Ke Za Zhi, 47(7), 559-564.
517. Zhu, L. P., Wang, F., Sun, X. Q., Chen, R. X., Lu, M. P., Yin, M., & Cheng, L. (2010). [Comparison of risk factors between patients with non-allergic rhinitis and allergic rhinitis]. Zhonghua Er Bi Yan Hou Tou Jing Wai Ke Za Zhi, 45(12), 993-998.
518. Zhu, R., Li, W., Wang, Z., Chen, H., Zhang, W., & Liu, G. (2008). [A survey of airborne pollen in Wuhan and its relationship to pollinosis]. Lin Chuang Er Bi Yan Hou Tou Jing Wai Ke Za Zhi, 22(14), 647-650.
519. Zhu, Y., Liu, Y., Zhu, X., Wang, Z., & Wang, M. (2020). Upregulation of miR-155 regulates group 2 innate lymphoid cells by targeting c-maf in allergic rhinitis. Eur J Pharmacol, 887, 173564. doi:10.1016/j.ejphar.2020.173564
520. Zhuang, Y., Ma, T., Kang, Z., Siqin, B., Yan, W., Bai, Y., . . . Wang, X. (2021). [Epidemiological survey of allergic rhinitis in steppe area of Xilingol League, Inner Mongolian of China]. Lin Chuang Er Bi Yan Hou Tou Jing Wai Ke Za Zhi, 35(11), 1004-1008. doi:10.13201/j.issn.2096-7993.2021.11.009
521. Zhuang, Y., Sun, X. M., Wang, X. Y., Shi, H. Y., Zhang, Z. G., & Wang, Q. (2010). [The influence of ambient air pollutants on outpatient visits for allergic disease and pollinosis]. Zhonghua Yu Fang Yi Xue Za Zhi, 44(12), 1121-1127.
522. Zuo, Z., Wang, J., Zhang, H., Zheng, W., Zhang, Z., & He, S. (2017). [Increased expressions of substance P and neurokinin/tachykinin receptor 1 in eosinophils of patients with psoriasis]. Xi Bao Yu Fen Zi Mian Yi Xue Za Zhi, 33(7), 966-970.
